# Targeting SLK protects against cerebral ischemia–reperfusion injury by regulating USP8-mediated HIF-1α stabilization and RhoA/ROCK activation

**DOI:** 10.1186/s11658-026-00988-8

**Published:** 2026-07-01

**Authors:** Yuetao Wen, Zhiyu Xiong, Zhiyuan Wang, Ya He, You Wang, Yuan Tao, Liping Huang, Chen Gong, Shuyu Jiang, Guo Du, Yangmei Chen, Tao Xu

**Affiliations:** 1 Department of Neurology, The Second Affiliated Hospital of Chongqing Medical University, Chongqing, 400010 China; 2 Department of Neurosurgery, Chongqing University Jiangjin Hospital, Chongqing, 402260 China; 3 Department of Neurology, The First Affiliated Hospital of Chongqing Medical University, Chongqing, 400042 China; 4 Department of Physical Examination Center, Chongqing University Jiangjin Hospital, Chongqing, 402260 China

**Keywords:** Acute ischemic stroke, SLK, USP8, HIF-1α/RhoA/ROCK pathway, Ubiquitination

## Abstract

**Introduction:**

Acute ischemic stroke (AIS) represents a major global contributor to mortality and chronic disability, with few effective therapeutic targets available. Identifying druggable genes associated with AIS is therefore critical for developing novel interventions.

**Methods:**

A comprehensive approach combining Mendelian randomization, multi-omics integration, and machine learning was employed to identify candidate druggable genes. Causal relationships were confirmed through genetic colocalization, and candidate genes were further validated in a retrospective clinical cohort comprising 60 patients who experienced AIS and 30 healthy controls. Functional and mechanistic investigations were performed using a male mouse middle cerebral artery occlusion/reperfusion (MCAO/R) model, oxygen–glucose deprivation/reperfusion (OGD/R) in HT22 cells, and 293 T cells.

**Results:**

SLK emerged as a genetically causal AIS gene and a central component of the optimal predictive model. Plasma SLK levels were elevated in patients who experienced AIS, demonstrating strong diagnostic and prognostic value and serving as an independent predictor of unfavorable 3-month outcomes after adjusting for age, sex, and baseline National Institutes of Health Stroke Scale (NIHSS) score. Knockdown of SLK conferred neuroprotection both in vivo and in vitro. SLK interacted with and phosphorylated the deubiquitinase USP8, increasing its activity and suppressing K48-linked polyubiquitination and degradation of HIF-1α. HIF-1α was stabilized following activation of the RhoA/ROCK signaling pathway, aggravating ischemic injury, whereas USP8 overexpression mitigated the neuroprotective effects of SLK knockdown.

**Conclusions:**

These findings identify SLK as a neuron-specific proischemic factor and a potential therapeutic target, elucidate the SLK–USP8–HIF-1α–RhoA/ROCK pathway as a key mechanistic pathway, and highlight plasma SLK as a promising diagnostic and prognostic biomarker with translational relevance.

**Graphical abstract:**

**Figure:**
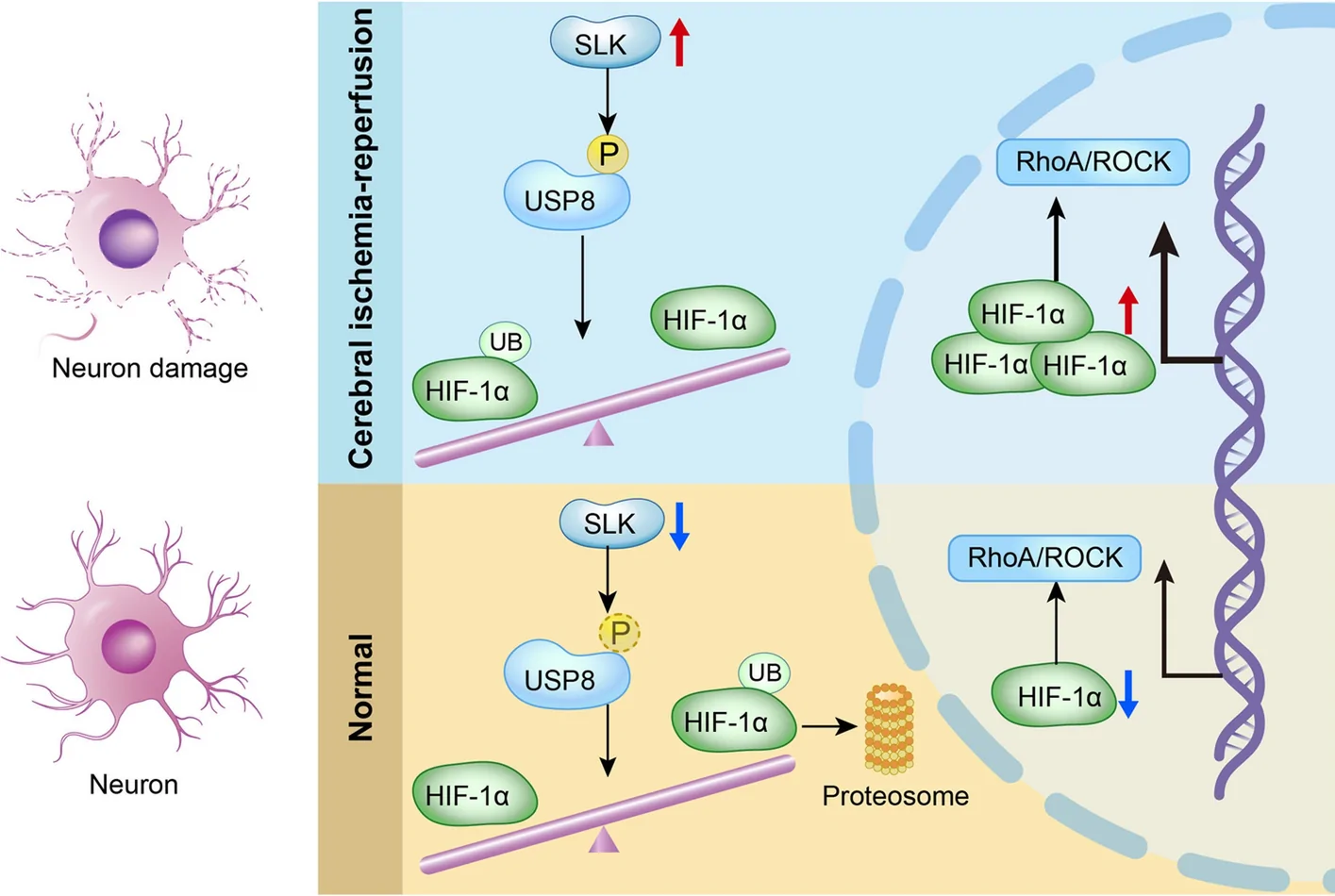

**Supplementary Information:**

The online version contains supplementary material available at https://doi.org/10.1186/s11658-026-00988-8.

## Introduction

Globally, stroke ranks as one of the most significant health challenges, being the second most common cause of death and the third most common cause of disability. The majority of stroke events are ischemic in nature, specifically classified as acute ischemic stroke (AIS) [1, 2]. AIS results from cerebral arterial occlusion, which impairs oxygen and nutrient delivery to brain tissue, causing ischemia and neuronal injury. Although timely restoration of cerebral blood flow is crucial for salvaging ischemic brain regions, reperfusion can itself initiate a chain of pathophysiological events, including apoptosis, necrosis, oxidative stress, excessive inflammation, dysregulated autophagy, intracellular calcium overload, blood–brain barrier disruption, extracellular matrix remodeling, and aberrant angiogenesis [3, 4]. These processes collectively exacerbate brain damage, leading to infarct expansion, cerebral edema, hemorrhagic transformation, and progressive neuronal loss, a syndrome referred to as cerebral ischemia–reperfusion injury (CIRI). Therapeutic options for CIRI remain limited, highlighting the need for detailed investigation of its mechanisms to uncover potential novel targets and strategies for clinical intervention.

Accurate identification of potential drug candidates for specific diseases, combined with systematic evaluation of their regulatory effects on disease progression, constitutes a fundamental component of the drug development process. Druggable genes encode proteins with structural properties that enable specific interactions with drugs, typically belonging to families such as ion channels, G protein-coupled receptors, and kinases. These proteins can engage with small-molecule compounds, therapeutic antibodies, or other biotherapeutics to modulate downstream signaling pathways or biological processes, ultimately yielding clinical benefits [5]. While approximately 4500 genes in the human genome are classified as druggable, only a few hundred of their encoded proteins are currently targeted by approved therapeutic agents, leaving a significant proportion unexplored or insufficiently characterized [6]. In the context of CIRI, limited research has focused on identifying druggable genes that could serve as treatment targets, and the precise molecular mechanisms through which these genes influence CIRI pathogenesis are yet to be fully defined.

Randomized controlled trials (RCTs) are considered the most rigorous method for assessing treatment effectiveness and safety. However, their execution is often constrained by practical limitations, including long study durations, high financial costs, and ethical considerations. In this context, human genetic data provide a robust framework for prioritizing drug targets by harnessing the random assortment of genotypes and the inherent resilience of genetic variants to environmental confounding, improving the probability of success in drug discovery. This approach is supported by Mendelian randomization (MR) and multi-omics analyses [7, 8]. MR employs genetic variants, typically single nucleotide polymorphisms (SNPs), as instrumental variables to determine causal linkage between risk factors and patient outcomes. The method exploits the random allocation of genes during meiosis, mimicking key principles underlying RCTs [9, 10]. Following the redefinition of the druggable genome by Finan et al., which integrated genome-wide association study (GWAS) data to systematically link genetic variants with potential drug targets, numerous studies have since employed genetic approaches to identify and validate disease-specific targets [5]. For instance, Duan et al*.* used MR analysis of the druggable genome to validate TBK1 as a prioritized target for drug repurposing in amyotrophic lateral sclerosis [11]. In addition to MR, multi-omics analyses provide a complementary approach for the identification of disease biomarkers and elucidation of relevant signaling pathways, therefore facilitating drug discovery [12].

In this study, cis-expression quantitative trait loci (cis-eQTL) data of the druggable genome were first obtained, and MR analysis with AIS GWAS data as the outcome variable was performed to detect druggable genes potentially linked to AIS. Integration of colocalization and multi-omics analyses highlighted STE20-like kinase (SLK), a member of the druggable genome, as a promising therapeutic target for AIS. Mechanistic investigations revealed that SLK activates the Ras homolog family member A (RhoA)/Rho-associated coiled-coil protein kinase (ROCK) axis by phosphorylating ubiquitin-specific protease 8 (USP8); therefore, elevating the deubiquitination and stabilization of hypoxia-inducible factor-1α (HIF-1α). The aim of this study is to identify and validate druggable genes associated with AIS, with a specific focus on establishing SLK as a therapeutic target in CIRI, and to elucidate the molecular mechanism by which SLK regulates ischemic injury through the USP8–HIF-1α–RhoA/ROCK signaling pathway.

## Materials and methods

### MR and colocalization

cis-eQTL data for blood samples corresponding to 2534 druggable genes (from the 4479 candidates reported by Finan et al.) were obtained from the eQTLGen database (www.eqtlgen.org) [5, 13]. Outcome data were retrieved from the GWAS Catalog (https://gwas.mrcieu.ac.uk; accession: ebi-a-GCST90018864), comprising 22,664 ischemic stroke cases and 472,192 controls of European ancestry. Another outcome data were retrieved from the R12 version of the Finnish database (https://r12.finngen.fi/) with the accession name “Stroke, excluding SAH.” Cis-eQTLs associated with druggable genes were used as IVs in MR, implemented using “TwoSampleMR” in R (version 0.5.7). IVs were selected according to three strict criteria: (1) significant association with the exposure (*P* < 5 × 10^−8^); (2) low linkage disequilibrium (LD), determined using the 1000 Genomes European reference panel with an r^2^ threshold < 0.1 and a clumping window of 10,000 kb; and (3) exclusion of weak instruments with F-statistics < 20 to minimize weak instrument bias. MR analyses were conducted using five complementary approaches: inverse variance-weighted (IVW), simple mode, weighted mode, weighted median, and MR Egger, with IVW designated as the primary method. Colocalization analysis was undertaken in “coloc” in R (version 5.2.3) according to established protocols [14]. A posterior probability of hypothesis 4 (PPH4) > 0.80 was regarded as robust evidence for colocalization, and only genes showing colocalization with ischemic stroke were subjected to further investigation.

### Preprocessing of bulk RNA

Microarray datasets related to AIS, specifically GSE16561, GSE22255, and GSE58294, were obtained from the gene expression omnibus (GEO) database. GSE16561 included 24 healthy control subjects and 39 patients experiencing AIS, GSE22255 comprised 20 healthy control subjects and 20 patients experiencing AIS, and GSE58294 included 23 healthy control subjects and 69 patients experiencing AIS. The GSE16561 and GSE22255 datasets were integrated via the “sva” package to alleviate batch effects. To assess batch correction, gene expression boxplots were created using “reshape2” and “ggplot2,” comparing data distributions before and after correction. DEGs in the training dataset were screened using “limma,” with a statistical threshold set as *p*-value < 0.05.

### Establishment and validation of diagnostic prediction model

A total of 113 diagnostic prediction models were developed using 12 machine learning algorithms, including stepwise generalized linear model (Stepglm), lasso regression, random forest (RF), generalized linear model boosting (glmBoost), support vector machines (SVM), ridge regression, elastic net regression (Enet), gradient boosting machines (GBM), eXtreme gradient boosting (XGBoost), partial least squares regression for generalized linear model (plsRglm), linear discriminant analysis (LDA), and naive Bayes classifiers (NaiveBayes), to identify potential biomarkers for AIS, following the methodology of a previous study [15]. The combined dataset from GSE16561 and GSE22255 functioned as the training set, while GSE58294 was used as an independent validation set. Model performance in distinguishing healthy controls from patients experiencing AIS was evaluated using area under the curve (AUC), with an AUC of 1 indicating perfect discrimination, and 0.5 indicating performance equivalent to random classification.

### Establishment of a nomogram

Nomogram analysis was performed using the top 10 candidate genes with the R package “rms” to predict AIS onset. Predictive accuracy of the nomogram was assessed through a calibration curve, while decision curves and clinical impact curves were generated to evaluate its clinical applicability.

### Unsupervised consensus clustering

Consensus clustering without supervision was applied to the expression profiles of candidate genes with the “ConsensusClusterPlus” package [16]. The analysis involved 1000 bootstrap resamplings, employing the “pam” algorithm and Euclidean distance. Cluster numbers (k) were tested from 2 to 10, and the optimal *k* was calculated from the cumulative distribution function (CDF) curve. The proportion of ambiguously clustered pairs (PAC) was further used to refine the selection of the optimal *k*, visualized using the “ggplot2” package.

### Functional enrichment and PPI analysis

Gene Ontology (GO) analyses were conducted to assess gene functions, using a threshold of *p* < 0.05. Protein–protein interaction (PPI) networks were then created using STRING [17]. The resulting PPI networks were imported into Cytoscape (version 3.10.3) to identify key nodes and visualize molecular interaction networks.

### Patients and SLK detection

This investigation was designed as a single-center retrospective observational investigation employing a prospectively curated stroke registry. We enrolled 60 patients experiencing AIS and 30 healthy controls admitted to the Second Affiliated Hospital of Chongqing Medical University from July 2024 to September 2025. Eligibility criteria comprised: (1) age > 18 years; (2) a confirmed diagnosis of AIS due to large vessel occlusion (LVO), verified by digital subtraction angiography (DSA) within 24 h of symptom onset; and (3) receipt of endovascular therapy within 24 h of the estimated time of LVO onset. Exclusion criteria included loss to follow-up and insufficient clinical data. An age-matched cohort of healthy individuals without a previous history of stroke was recruited as the control group. All participants underwent cranial magnetic resonance imaging (MRI), including diffusion-weighted imaging and magnetic resonance angiography, to rule out asymptomatic cerebral infarction and arterial stenosis. Venous blood samples were obtained within 24 h of symptom onset and from healthy control subjects at enrollment. Samples were collected in ethylenediaminetetraacetic acid (EDTA) anticoagulant Vacutainer tubes and processed within 1 h by centrifugation at 1280 *g* for the duration of 10 min at a temperature of 4 °C to separate plasma, which was preserved at −80 °C. Plasma SLK concentrations were measured using an ELISA kit (Jiangsu Meibiao Biotechnology, catalog no. MB-00874A) as directed. The study was approved by the Human Research Ethics Committee of the Second Affiliated Hospital of Chongqing Medical University, Chongqing, China, and all procedures complied with the Declaration of Helsinki. Written informed consent was obtained from each participant or an authorized legal representative before inclusion in the study.

### Animals

Sex-related differences in brain oxidative stress, mitochondrial function, and inflammatory responses have been well documented [18, 19]. To minimize experimental variability associated with sex hormone fluctuations, adult male C57BL/6 J mice were purchased from the Experimental Animal Center at Chongqing Medical University. The mice were between 8 and 10 weeks of age at the time of the experiments, with body weights ranging from 20 to 25 g. Following procurement, all animals were maintained in a standardized laboratory environment to ensure consistent experimental conditions, with a temperature of 22 °C (±1 °C), relative humidity between 50 and 60%, and a regular 12-h light and 12-h dark photoperiod to maintain their natural circadian rhythms. Throughout the study period, animals had unrestricted food and water. All procedures involving animals were performed in strict compliance with institutional guidelines and ethical standards. The Animal Care and Use Committee of Chongqing Medical University granted ethical approval for the experimental protocol. The experimental protocol received approval from the Animal Care and Use Committee of Chongqing Medical University.

### Lentivirus administration

Recombinant lentiviral vectors encoding sh-SLK or sh-con were generated and packaged by GenePharma Co., Ltd. (Shanghai, China). All lentiviral constructs incorporated the H1 promoter, a green fluorescent protein reporter, and a puromycin resistance gene. The shRNA target sequences were: sh-SLK (#1), GTCTATAAGGCCCAGAATAAA; sh-SLK (#2), GTCCCTGGGTATTACTTTAAT; sh-SLK (#3), ACCTTAGATGGAGACATTAAA; and the nontargeting control sh-con, TTCTCCGAACGTGTCACGT. Stereotaxic intracortical administration of sh-SLK or sh-con lentiviral vectors was carried out in mice. Injections were directed to the cerebral cortex using the following coordinates relative to the bregma: 1.2 mm anterior, 1.2 mm lateral from the midline, and 3.0 mm ventral from the skull surface. Each mouse received a total of 9 μL of purified lentiviral suspension delivered at 0.5 μL/min. Following injection completion, the needle was maintained in position for 10 min before being slowly removed to avoid cerebrospinal fluid reflux and viral vector escape. After a 3-week interval post-lentiviral delivery, we evaluated transduction success through immunofluorescence staining for GFP and western blot analysis for SLK protein levels. The sh-SLK construct demonstrating the highest knockdown efficiency was chosen for further experiments.

### Middle cerebral artery occlusion/reperfusion (MCAO/R) model and animal groups

Mice were anesthetized with 1% pentobarbital sodium (*i.p.*, 100 mg/kg). A longitudinal cervical incision (~1.5 cm) was made along the anterior midline to expose the external carotid artery (ECA), right common carotid artery (CCA), and internal carotid artery (ICA). The proximal CCA and distal ECA were ligated, after which a nylon monofilament (0.21 mm diameter) was retrogradely introduced into the ICA via the ECA, followed by advancement and occlusion of the origin of the middle cerebral artery (MCA), leading to focal cerebral ischemia for 60 min. Reperfusion was initiated by withdrawing the filament and releasing the vascular occlusion. Mice in the sham group were subjected to identical anesthesia along with surgical procedures, with the exception that no filament was introduced into the ICA or progressed toward the MCA territory. Mice were randomly divided into four groups (*n* = 6 per group): sham, MCAO/R, MCAO/R + sh-con, and MCAO/R + sh-SLK. Mice in the MCAO/R + sh-con and MCAO/R + sh-SLK groups were injected with the corresponding lentivirus and then subjected to MCAO/R surgery. Mice in the sham group received the same surgical exposure of the CCA region without undergoing MCA occlusion or reperfusion.

### 2,3,5-Triphenyltetrazolium chloride (TTC) staining

At 24 h post-MCAO/R surgery, animals were euthanized, and brains were immediately extracted. Each brain was cut into four serial coronal sections, each 2-mm thick, to enable systematic evaluation of infarct distribution. The brain sections were then immersed in TTC solution (Beyotime Biotechnology, catalog no. C0652) and kept at 37 °C under dark conditions for 15–30 min. This staining method allows viable tissue with active mitochondrial enzymes to appear red, while infarcted regions lacking enzyme activity remain white.

Following staining, digital images of each slice were captured and analyzed using ImageJ software (NIH, USA). To account for cerebral edema following ischemia, infarct volume was calculated with edema correction using the following formula:

$$\mathrm{Infarct}\ \mathrm{volume} = \frac{(\text{contralateral hemisphere area - ipsilateral non-infarcted area})}{(2 \times \text{contralateral hemisphere area})} \times 100\%$$

### Neurobehavioral assessment

Neurological deficits were evaluated 24-h following surgery using the modified neurological severity score (mNSS). The mNSS is an 18-point composite scale assessing motor function, reflexes, and balance, with higher scores indicating greater neurological impairment.

### Cell culture

The mouse hippocampal neuronal cell line HT22 (Procell, catalog no. CL-0697) and HEK293T cell line (Procell, catalog no. CL-0005) were maintained in Dulbecco’s modified Eagle medium (DMEM) (catalog no. 11965092, Thermo Fisher Scientific) with high glucose, 10% FBS (catalog no. 10099141C, Thermo Fisher Scientific), and 1% penicillin/streptomycin (catalog no. 15140122, Thermo Fisher Scientific) at 37 °C with 5% CO_2_ incubator.

### Lentivirus transfection

HT22 cells (2 × 10^5^/well) were inoculated in cell culture grade six-well plates to generate lines stably expressing sh-con or sh-SLK. Lentiviral vectors were added to the medium at a multiplicity of infection (MOI) of 40, along with 5 µg/mL polybrene (GenePharma) to elevate transduction efficiency. After 24 h, stable transfectants were selected by treatment with 5 µg/mL puromycin (Biosharp, catalog no. BL528A) for 3 consecutive days. Surviving cells were collected for use in further experiments.

### Oxygen–glucose deprivation/reperfusion (OGD/R) model

Normal HT22 cells and their sh-con or sh-SLK stably transfected counterparts were subjected to OGD/R to model in vitro I/R injury, following a previously described protocol with minor modifications [20]. Briefly, culture media were replaced with sugar-free DMEM (catalog no. 11966025, Thermo Fisher Scientific), and cells were maintained under hypoxic conditions (1% O_2_, 5% CO_2_, 95% N_2_) for 2 h to induce OGD. Reperfusion was simulated by returning the cells to normoglycemic DMEM in a standard incubator for 24 h.

### Plasmid transfection

All plasmids used in this study were constructed on the pcDNA3.1 backbone and obtained from QEgene Biotechnology Co., Ltd. (Shanghai, China). These included Myc-tagged SLK, His-tagged USP8, His-tagged USP8 mutants (S716A, S716D, C786A), Flag-tagged HIF1α, HA-tagged ubiquitin wild-type (WT), HA-tagged ubiquitin chain-type-specific plasmids (K6, K11, K27, K29, K33, K48, K63), and HA-tagged ubiquitin lysine-to-arginine mutant (K48R). Plasmid transfection was performed using PEI (catalog no. HY-K2014, MCE) according to the manufacturer’s instructions.

### Co-immunoprecipitation (Co-IP)

Cortical tissues and cells were lysed in lysis buffer (Beyotime, catalog no. P0013J) containing a protease inhibitor cocktail (Med Chem Express, catalog no. HY-K0010) and spun at 12,000 × *g* for the duration of 15 min. Protein levels were assessed using a BCA Protein Assay Kit (catalog no. P0010, Beyotime). Equivalent volumes of lysate were treated overnight at 4 °C with antibodies: rabbit IgG (catalog no. 30000-0-AP, Proteintech), mouse IgG (catalog no. B900620, Proteintech), rabbit anti-SLK (Proteintech, catalog no. 19743-1-AP), rabbit anti-USP8 (catalog no. 27791-1-AP, Proteintech), rabbit anti-HIF1α (catalog no. 20960-1-AP, Proteintech), mouse anti-HIF1α (catalog no. 66730-1-IG, Proteintech), mouse anti-Myc (catalog no. 60003-2-IG, Proteintech), mouse anti-His (catalog no. 66005-1-IG, Proteintech), or mouse anti-Flag (catalog no. 66008-4-IG, Proteintech). This was followed by mixing with protein A/G magnetic beads (catalog no. HY-K0202, Med Chem Express) for 2 h, three PBS washes, and boiling at 100 °C for 10 min before western blotting.

### Western blotting

Lysis buffer (Beyotime, catalog no. P0013J) with protease inhibitors (Med Chem Express, catalog no. HY-K0010) was used to extract the total protein, followed by assessment of protein levels using BCA assays as above. Protein samples of equal concentration were resolved on sodium dodecyl sulfate polyacrylamide gel electrophoresis (SDS-PAGE) and subsequently electroblotted onto polyvinylidene fluoride (PVDF) membranes. After blocking with Tris-buffered saline solution containing 5% non-fat milk, the blots were treated with appropriate primary antibodies through overnight incubation at 4 °C. The antibodies were against SLK (19743-1-AP, Proteintech, 1:1000), USP8 (27791-1-AP, Proteintech, 1:1000), HIF1α (20960-1-AP, Proteintech, 1:1000), Bax (50599-2-Ig, Proteintech, 1:1000), Bcl-2 (26593-1-AP, Proteintech, 1:1000), Cleaved Caspase-3 (10971, CST, 1:1000), IL-1β (26048-1-AP, Proteintech, 1:1000), IL-6 (26404-1-AP, Proteintech, 1:1000), TNFα (17590-1-AP, Proteintech, 1:1000), RhoA (10749-1-AP, Proteintech, 1:1000), ROCK1 (21850-1-AP, Proteintech, 1:1000), ROCK2 (21645-1-AP, Proteintech, 1:1000), Ubiquitin (10201-2-AP, Proteintech, 1:1000), Phosphoserine (ab9332, Abcam, 1:1000), Phosphothreonine (9386, CST, 1:1000), Myc (16286-1-AP, Proteintech, 1:1000), His (10001-0-AP, Proteintech, 1:1000), Flag (20543-1-AP, Proteintech,1:1000), HA (51064-2-AP, Proteintech, 1:1000), and β-actin (20536-1-AP, Proteintech, 1:2000). Membranes were then incubated with HRP-linked goat anti-rabbit IgG (RGAR001, Proteintech, 1:2000) or goat anti-mouse IgG (RGAM001, Proteintech, 1:2000) for 1 h at room temperature and visualized using enhanced chemiluminescence (ECL) reagent.

### Immunoprecipitation coupled with mass spectrometry (IP-MS)

Total proteins were extracted from OGD/R-treated HT22 cells using the lysis buffer. The IP complex, comprising SLK antibody, protein A/G magnetic beads, and associated target proteins, was prepared as described previously. Peptides derived from the IP complex were separated using a nanoElute^TM^ system and analyzed on a timsTOF Pro^TM^ mass spectrometer (both Bruker Daltonics, Bremen, Germany) operated in Parallel Accumulation-Serial Fragmentation mode to increase peptide identification. Raw MS data were analyzed using MaxQuant software (version 1.6.14) using the following parameters: trypsin as the protease allowing up to 2 missed cleavages, the UniProt Mus musculus database (custom index: uniprot_Mus_musculus_87491_20250111) as the reference, carbamidomethylation of cysteine (C) as a fixed modification, oxidation of methionine (M) as a variable modification, peptide mass tolerance of 20 ppm, fragment mass tolerance of 0.1 Da, protein and peptide FDR set at 0.01 (1%), and intensity-based absolute quantification (iBAQ) quantification enabled (set to TRUE).

### Immunofluorescence staining

Paraffin-embedded mouse brain sections and cell climbing glass coverslips from HT22 and HEK293T cells were labeled using the Flexible Coralite Antibody Labeling Kits following the manufacturer’s instructions (catalog nos. KFA501, KFA502, KFA503, Proteintech). Primary antibodies included: SLK, USP8, HIF1α, Cleaved Caspase-3, Ubiquitin, Myc, His, Flag, NeuN (Proteintech, catalog no. 26975-1-AP), and GFAP (Proteintech, catalog no. 16825-1-AP). Labeled sections were visualized using a Leica confocal microscope (TCS SP8 X).

### EdU staining

EdU assays were utilized as directed to assess the effect of SLK knockdown on HT22 cell proliferation under OGD/R conditions (Beyotime, catalog no. C0075S). Imaging was performed using a Leica confocal microscope.

### Reactive oxygen species (ROS) staining

Intracellular ROS levels in brain tissues and HT22 cells were measured using a ROS detection kit (Elabscience, catalog no. E-BC-F005) as directed, with imaging using a Leica confocal microscope.

### Detection of superoxide dismutase (SOD), catalase (CAT) and malondialdehyde (MDA)

SOD and CAT activities and MDA contents of brain tissues and HT22 cells were estimated using the Total SOD Assay Kit with WST-8 (catalog no. S0101, Beyotime), CAT Assay Kit (Beyotime, catalog no. S0051), and Lipid Peroxidation MDA Assay Kit (catalog no. S0131, Beyotime), respectively, as directed.

### Statistical analysis

Data were analyzed using R software (version 4.5.0) in combination with GraphPad Prism (version 10.4.2). Normally distributed continuous variables are reported as mean ± SD, while skewed data are given as median ± interquartile range (IQR). Categorical variables are shown as frequencies and percentages. Two independent groups of normally distributed data were compared using *t*-tests, whereas the Mann–Whitney *U* test was utilized for data that did not follow a normal distribution. Three or more groups of normally distributed data were evaluated using one-way analysis of variance (ANOVA) followed by Tukey’s post-hoc test, while non-normally distributed data were compared using Kruskal–Wallis *H* tests, followed by Dunn’s post-hoc analysis. Throughout all statistical evaluations, a two-tailed significance threshold of *p* < 0.05 was applied to determine statistical significance.

## Results

### Identification of AIS-related druggable genes via multi-omics analyses

**Fig. 1:**
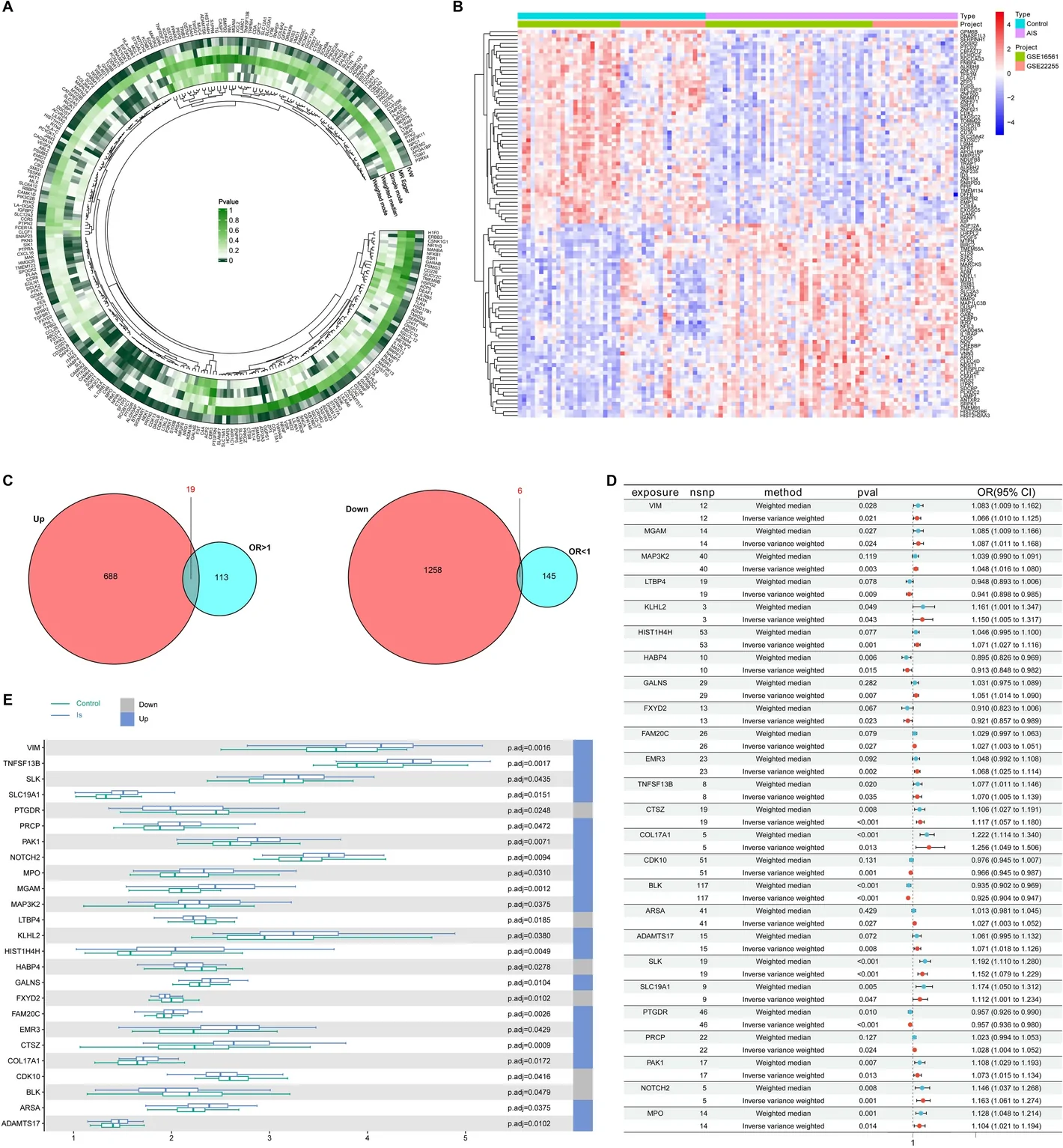
Identification of AIS-related druggable genes via multi-omics analyses. **A** Circular heatmap of MR results for candidate genes associated with AIS. **B** Heatmap of gene expression patterns between patients experiencing AIS and controls in merged datasets. **C** Venn diagrams based on MR results and differential analysis. **D** Forest plot of causal effect parameters for candidate druggable genes. **E** Differential expression of candidate druggable genes between AIS and control groups

Causal associations between 2888 blood-derived druggable genes and AIS were systematically assessed, revealing 283 genes with statistically significant causal links to AIS (Fig. 1A, Supplementary Table S1). After batch correction and integration of the GSE16561 and GSE22255 datasets, gene expression distributions across the two cohorts were well-aligned, confirming effective removal of technical batch effects (Supplementary Fig. 1). Differential expression analysis identified 1971 genes showing significant expression changes, including 707 upregulated and 1264 downregulated genes (Fig. 1B, Supplementary Table S2). By integrating MR and differential expression results, 19 genes were classified as AIS-promoting, based on upregulated expression and OR > 1, whereas six genes were considered AIS-inhibitory, characterized by downregulated expression and OR < 1 (Fig. 1C–E).

### Construction and validation of a druggable gene-based prognostic model for AIS

**Fig. 2:**
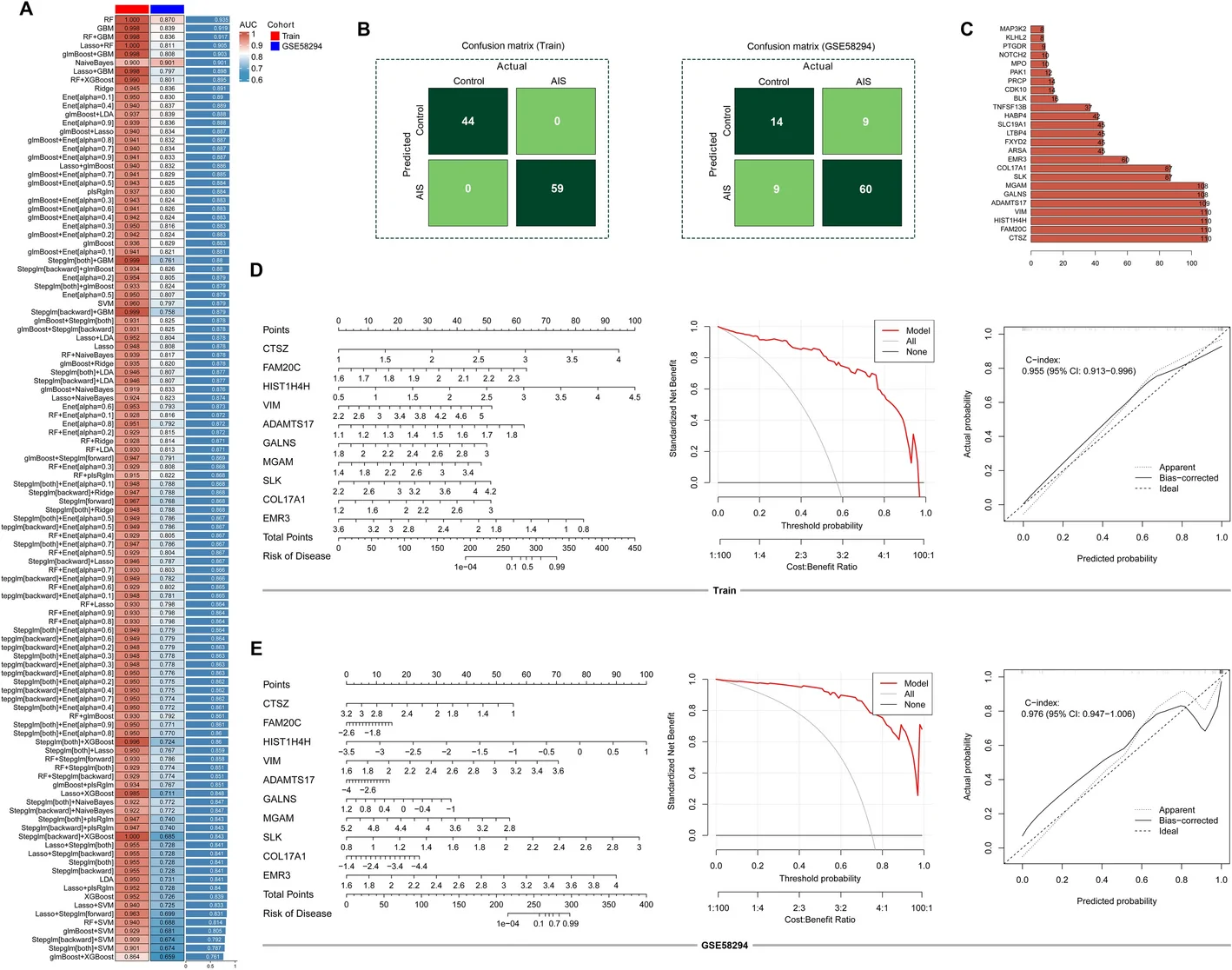
Construction and validation of a druggable gene-based prognostic model for AIS. **A** Performance metrics of 113 machine learning algorithms. **B** Confusion matrices for training set and GSE82244 cohort classification. **C** Feature importance ranking of candidate druggable genes. **D** Training cohort nomogram, decision curve, and C-index plot. **E** GSE82244 cohort nomogram, decision curve, and C-index plot

The predictive performance of 113 machine learning algorithms was initially evaluated to screen 25 candidate druggable genes, with RF identified as the optimal model due to its superior predictive efficacy (Fig. 2A, Supplementary Table S3). In the training cohort comprising 44 controls and 59 AIS cases, RF achieved near-perfect classification with no misclassifications and demonstrated robust generalization in the external GSE82244 cohort of 14 controls and 60 AIS cases, yielding only nine misclassifications (Fig. 2B). Feature recurrence analysis across all 113 algorithms quantified the frequency of candidate genes, confirming the consistent contribution of core genes to model performance (Fig. 2C, Supplementary Table S4). Using the top 10 genes with the most significant recurrence frequency, nomograms were constructed for both the training and test cohorts to evaluate model robustness. Decision curve analysis and a C-index of 0.955 (95% CI, 0.930–0.980) confirmed the clinical utility of the training cohort nomogram (Fig. 2D), while a C-index of 0.976 (95% CI, 0.947–1.006) validated reproducibility in the test cohort (Fig. 2E). Consensus clustering of the 25 druggable genes stratified samples into subgroups, with a consensus matrix (*k* = 2) illustrating cluster stability (Supplementary Fig. 2A) and principal component analysis (PCA) confirming distinct separation between cluster A and cluster B (Supplementary Fig. 2B). Comparative analysis of candidate gene expression across clusters revealed clear subgroup-specific trends (Supplementary Fig. 2C). Genes including *GALNS, SLK, NOTCH2, MAP3K2, EMR3, KHL2, MGAM*, and *TNFSF13B* were significantly upregulated in cluster B relative to cluster A, whereas *LTBP4* and *EXYD2* were significantly downregulated.

### Genetic colocalization and clinical biomarker validation of SLK in AIS

**Fig. 3:**
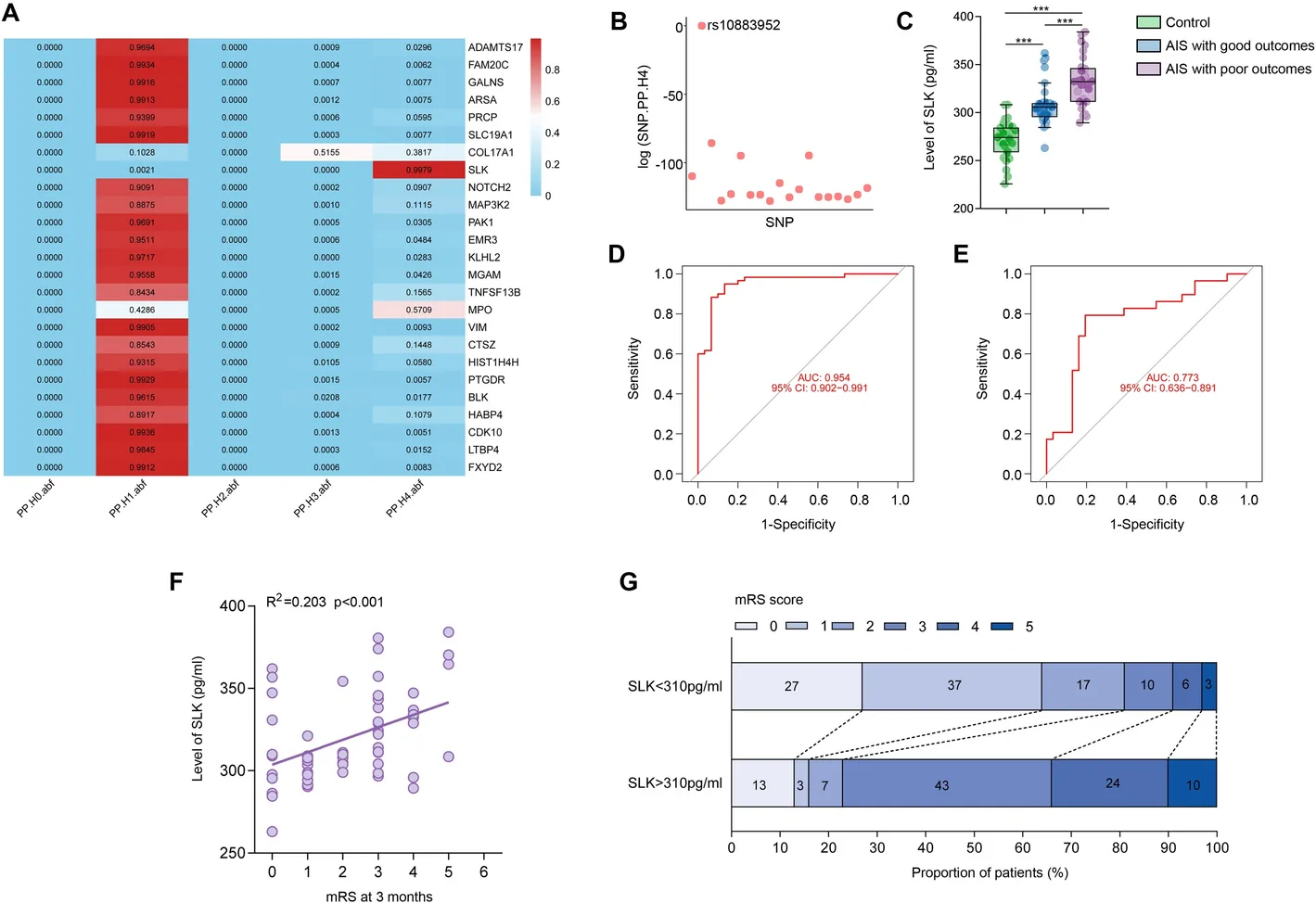
Genetic colocalization and clinical biomarker validation of SLK in AIS. **A** Bayesian colocalization of 25 druggable genes and AIS susceptibility. **B** Lead colocalized SNP rs10883952 for SLK and AIS pathogenesis. **C** Plasma SLK levels in controls (*n* = 30), AIS cases with good (*n* = 29) and poor prognosis (*n* = 31). **D** ROC curve for AIS diagnosis via plasma SLK. **E** ROC curve for poor AIS prognosis via plasma SLK. **F** Correlation between plasma SLK levels and 90-day mRS scores in individuals with AIS. **G** Distribution of 3-month mRS scores in individuals with AIS stratified by plasma SLK cut-off (310 pg/ml). ***p < 0.001

Bayesian colocalization analysis of 25 druggable genes revealed strong colocalization between SLK and AIS (PP.H4 = 0.9979), indicating that SLK is a genetically causal gene for AIS susceptibility (Fig. 3A). rs10883952 was identified as the lead colocalized SNP showing the strongest causal association with AIS pathogenesis (Fig. 3B). Independent MR analysis, using SLK as exposure and a Finnish AIS cohort as the outcome, confirmed a consistent causal relationship between SLK and AIS risk (Supplementary Fig. 3A–D). A retrospective observational study including 30 healthy controls and 60 AIS cases was conducted, with baseline clinical characteristics summarized in Supplementary Table S5. AIS cases were grouped on the basis of 3-month functional outcomes into favorable prognosis (mRS 0–2) and unfavorable prognosis (mRS 3–6) subgroups. Consistent with multi-omics findings, ELISA quantification demonstrated significantly elevated plasma SLK levels in AIS cases, particularly in those with poor prognosis, compared with healthy controls and patients with favorable outcomes (Fig. 3C). Plasma SLK showed strong discriminatory performance for distinguishing individuals with AIS from healthy controls, with an AUC of 0.954 (95% CI: 0.902–0.991; Fig. 3D). For predicting poor functional outcomes among AIS cases, SLK was associated with an AUC of 0.773 (95% CI: 0.636–0.891; Fig. 3E). A marked positive link was found between plasma SLK levels and 3-month mRS scores (R^2^ = 0.203, *P* < 0.001; Fig. 3F). Multivariate logistic regression, adjusted for age, sex, and baseline National Institutes of Health Stroke Scale (NIHSS) score, confirmed that elevated plasma SLK levels (per 1 pg/mL increment) were an independent predictor of poor 3-month outcomes (OR = 1.044, 95% CI: 1.011–1.089, *p* = 0.002). Using a threshold of 310 pg/mL to stratify AIS cases, individuals with elevated SLK levels (> 310 pg/mL, *n* = 30) displayed significantly worse functional outcomes compared with those with lower SLK levels (< 310 pg/mL, *n* = 30), as represented by ordinal comparison of 3-month mRS distributions (*p* < 0.001; Fig. 3G).

### SLK knockdown attenuates I/R injury in animal and cellular models

**Fig. 4:**
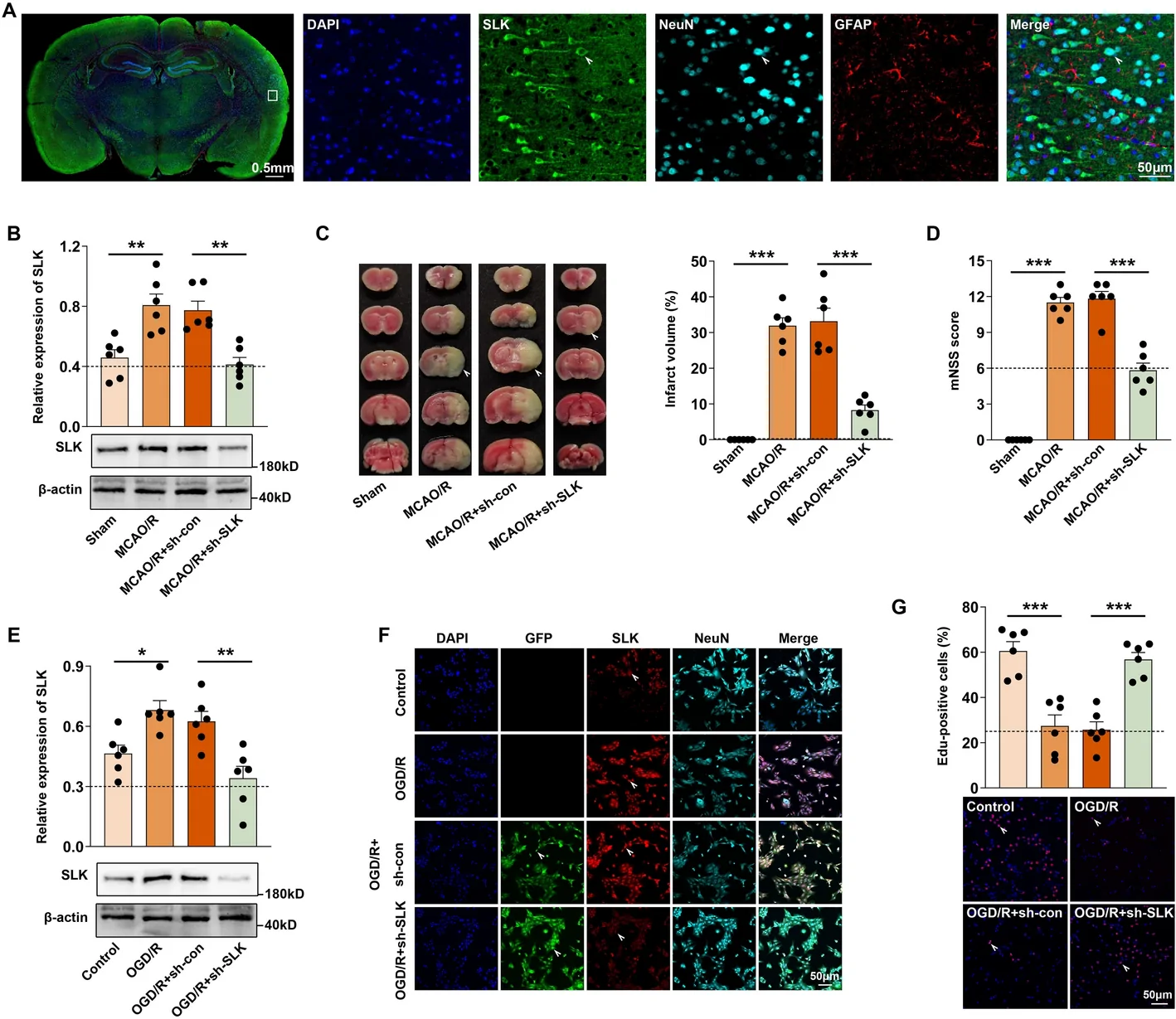
SLK knockdown attenuates I/R injury in animal and cellular models. **A** Whole-brain SLK fluorescence (left) and colocalization with neurons in ischemic cortex. **B** SLK protein expression in MCAO/R brain tissues. **C** Cerebral infarct in MCAO/R mice. **D** mNSS score in MCAO/R mice. SLK protein expression in OGD/R cells detected by western blotting **(E)** and immunofluorescence staining **(F)**. **G** EdU staining of HT22 cells under OGD/R treatment. *n* = 6/per group, *p < 0.05, **p < 0.01, ***p < 0.001

For the investigation of the functional role of SLK in AIS, its cellular localization was examined in a mouse model of MCAO/R using immunofluorescence. SLK was predominantly colocalized with the NeuN^+^, associated with neurons, rather than GFAP^+^, expressed on astrocytes, in the ischemic cortex (Fig. 4A), indicating neuron-enriched expression in ischemic tissue. This neuronal specificity suggested that SLK’s contribution to AIS pathogenesis is primarily mediated via neurons, justifying later targeted knockdown in neuronal populations for functional studies.

Three recombinant lentiviral constructs carrying short hairpin RNAs (shRNA) targeting SLK (sh-SLK) and a corresponding scramble control (sh-con) were evaluated for neuronal transfection. Immunofluorescence staining confirmed efficient transduction in neuronal cells (Supplementary Fig. 4A), and western blot analysis demonstrated that all three sh-SLK constructs reduced SLK protein levels relative to sh-con, with sh-SLK (#2) achieving the highest silencing efficiency (Supplementary Fig. 4B). This construct was selected for all the following animal experiments. SLK protein levels were substantially raised in the MCAO/R mice relative to the sham controls, and this ischemia-induced increase was partially reversed by sh-SLK-mediated knockdown (Fig. 4B). Functional evaluation in MCAO/R mice revealed that SLK knockdown reduced cerebral infarct volume (Fig. 4C) and improved neurological outcomes (Fig. 4D). Consistent with in vivo findings, sh-SLK (#2) achieved efficient SLK silencing in HT22 neuronal cells (Supplementary Fig. 4C). Neuronal cells exposed to OGD/R showed significant upregulation of SLK, which was effectively attenuated by sh-SLK treatment (Fig. 4E, F). SLK knockdown significantly improved neuronal viability under OGD/R-induced ischemic conditions (Fig. 4G). These results identify SLK as a neuron-specific proischemic factor and demonstrate that its targeted knockdown is neuroprotective in both animal and cellular models of AIS.

### SLK knockdown mitigates I/R-induced ROS production, oxidative stress, apoptosis, and neuroinflammation in animal and cellular models

**Fig. 5:**
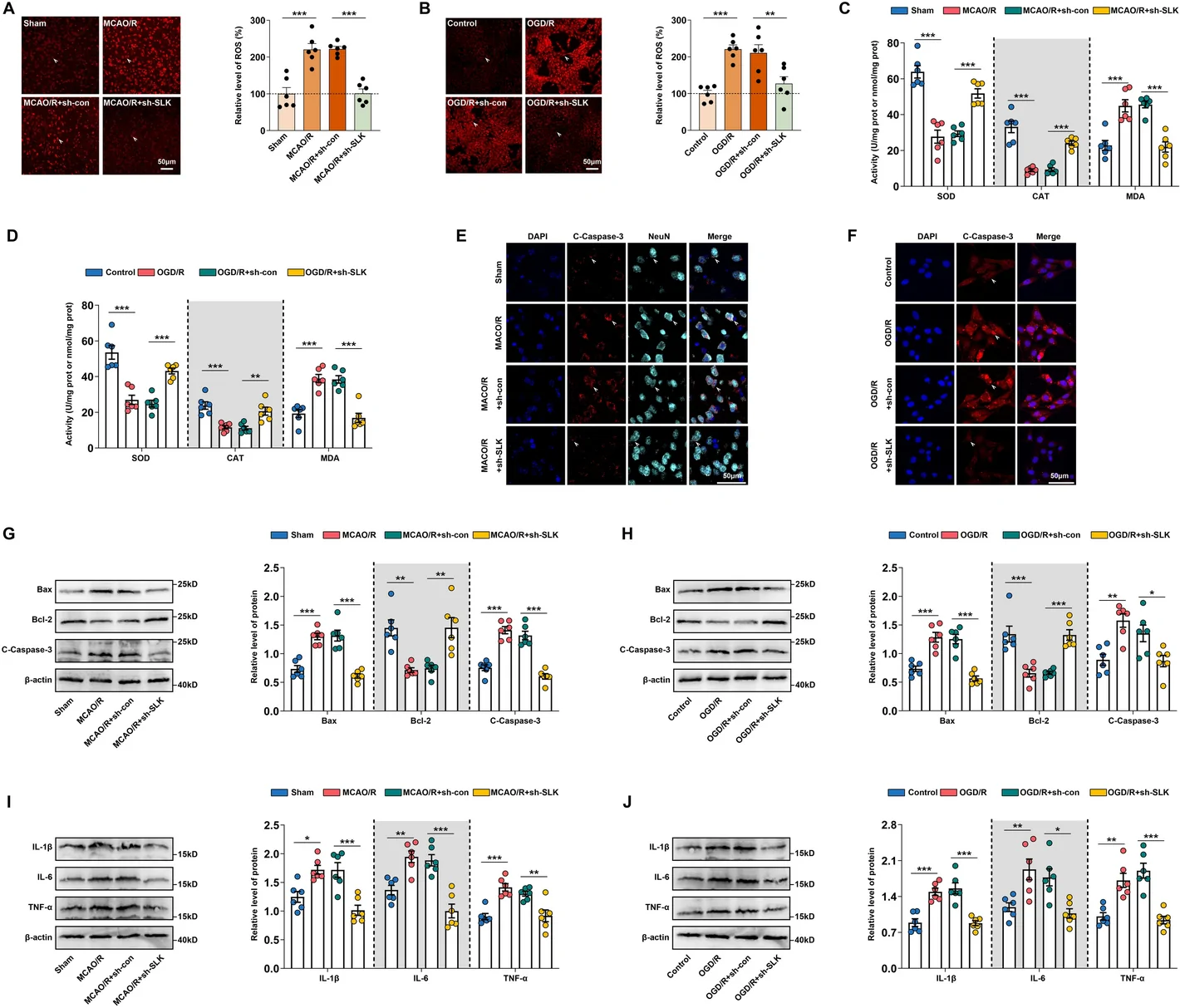
SLK knockdown mitigates I/R-induced ROS production, oxidative stress, apoptosis, and neuroinflammation in animal and cellular models. **A** ROS accumulation detected by fluorescence assay in MCAO/R mouse brain tissues. **B** ROS production detected by fluorescence assay in OGD/R-treated neuronal cells. **C** SOD activity, CAT activity and MDA levels measured in MCAO/R mouse brain tissues. **D** SOD activity, CAT activity and MDA content measured in OGD/R-treated neuronal cells. **E** Cleaved caspase-3 expression assessed by immunofluorescence staining in MCAO/R mouse brain tissues. **F** Cleaved caspase-3 expression assessed by immunofluorescence staining in OGD/R-treated neuronal cells. **G** Protein expression of Bax, cleaved caspase-3, and Bcl-2 determined by western blotting in MCAO/R mouse brain tissues. **H** Protein expression of Bax, cleaved caspase-3 and Bcl-2 determined by western blotting in OGD/R-treated neuronal cells. **I** IL-1β, IL-6 and TNF-α levels quantified in MCAO/R mouse brain tissues. **J** IL-1β, IL-6 and TNF-α levels quantified in OGD/R-treated neuronal cells. *n* = 6/per group, *p < 0.05, **p < 0.01, ***p < 0.001

To elucidate the pathways underlying SLK-mediated ischemic injury, key pathological processes, including ROS accumulation, oxidative stress, neuronal apoptosis, and neuroinflammation, were assessed in both animal and cellular models. In MCAO/R mouse brain tissues, SLK knockdown reduced ROS accumulation (Fig. 5A) and restored antioxidant defenses, as indicated by increased CAT and SOD activities and decreased MDA levels (Fig. 5C). SLK silencing in OGD/R-treated neuronal cells mitigated ROS overproduction (Fig. 5B) and normalized SOD and CAT activity as well as MDA content (Fig. 5D). Apoptotic signaling was next examined. Immunofluorescence revealed that SLK knockdown decreased cleaved caspase-3 expression in both the brains of MCAO/R mice (Fig. 5E) and OGD/R cells (Fig. 5F). Western blotting confirmed reductions in the levels of cleaved caspase-3 and proapoptotic Bax, together with increases in those of the anti-apoptotic Bcl-2, in both animal (Fig. 5G) and cellular (Fig. 5H) models. Proinflammatory cytokine expression was also assessed, demonstrating that SLK knockdown substantially lowered the levels of interleukin (IL)-6, IL-1β, and tumor necrosis factor (TNF)-α in MCAO/R brain tissue (Fig. 5I) and OGD/R neuronal cells (Fig. 5J). These results indicate that SLK knockdown mitigates ischemic injury by suppressing ROS production, oxidative stress, neuronal apoptosis, and neuroinflammation in both animal and cellular models.

### SLK regulates ischemic injury via the HIF-1α–RhoA/ROCK axis

**Fig. 6:**
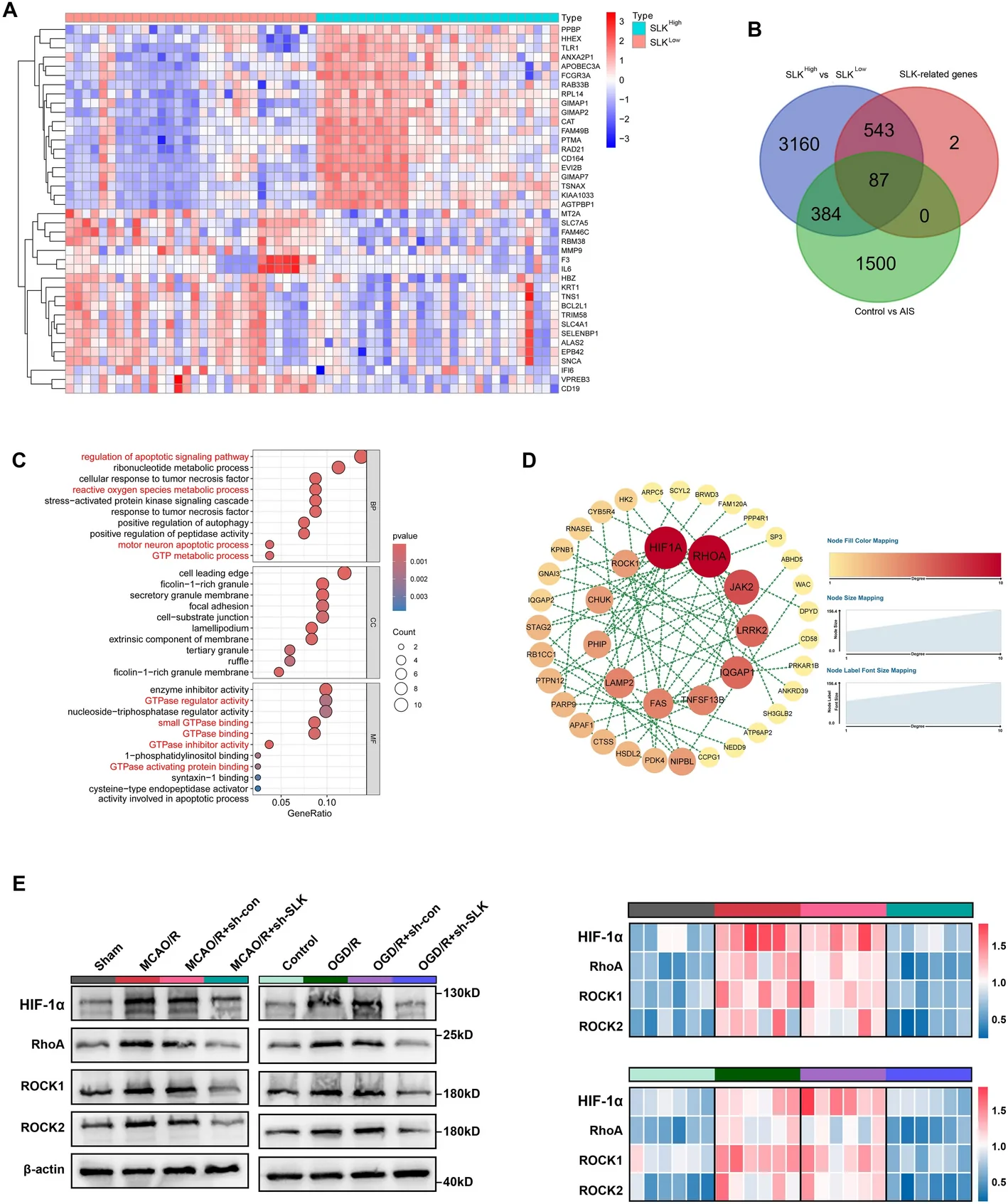
SLK regulates ischemic injury via the HIF-1α–RhoA/ROCK axis. **A** Heatmap showing 4174 differentially expressed genes in AIS cases with high or low SLK expression in the training cohort. **B** Venn diagram showing the overlapping genes among SLK subgroup signature genes, AIS versus control differentially expressed genes and SLK-correlated genes. **C** Functional enrichment analysis of the 87 overlapping genes. **D** PPI network analysis. **E** Protein expression levels of HIF-1α, RhoA, ROCK1 and ROCK2 detected by western blotting in MCAO/R mouse brain tissues and OGD/R-treated neuronal cells with SLK knockdown or control treatment

To identify SLK-associated molecular pathways in AIS, patients in the training cohort were stratified into high- and low-SLK expression subgroups. Differential gene expression analysis between these subgroups identified 4174 genes showing distinct transcriptional profiles, which were defined as the SLK subgroup signatures (Fig. 6A, Supplementary Table S6). Two complementary gene sets were also generated for integrative analyses: the first comprised 1971 DEGs between AIS cases and controls in the training cohort (Supplementary Table 2), and the second included 632 SLK-correlated genes, defined by a correlation coefficient |*r*|> 0.5 and *p* < 0.01 (Supplementary Table 7). A total of 87 genes were shared among the SLK subgroup signatures, AIS versus control DEGs, and SLK-correlated genes (Fig. 6B,, Supplementary Table S8). Functional enrichment analysis of these overlapping genes identified pathways integral to ischemic injury, including apoptotic signaling, GTPase activity, and ROS metabolism (Fig. 6C, Supplementary Table S9). PPI network analysis further identified *HIF-1α* and *RhoA* as central hub genes, with ROCK1 and ROCK2 serving as key downstream effectors within this SLK-associated network (Fig. 6D). HIF-1α is a well-characterized mediator of I/R injury and emerged as a principal SLK-associated hub gene in our analysis. RhoA, a canonical member of the Rho GTPase subfamily within the Ras superfamily, transduces signals by its ability to cycle between active GTP-bound and inactive GDP-bound states, regulating multiple downstream cellular processes relevant to ischemic pathology. Activation of RhoA by HIF-1α triggers the downstream ROCK1/ROCK2 signaling cascade, which regulates ROS production, oxidative stress, neuronal apoptosis, and neuroinflammation. This pathway provides a mechanistic explanation for the protective effects of SLK knockdown on these key pathological processes in I/R injury. In vitro and in vivo validation demonstrated that ischemia-induced SLK upregulation in MCAO/R mouse brains and OGD/R neurons was accompanied by increased protein levels of HIF-1α, RhoA, ROCK1, and ROCK2. SLK knockdown significantly reduced the expression of these proteins (Fig. 6E). These findings establish that SLK knockdown mitigates I/R injury by suppressing the HIF-1α–RhoA/ROCK signaling axis, thus mediating neuroprotection through modulation of ROS production, oxidative stress, neuronal apoptosis, and neuroinflammation.

### SLK stabilizes HIF-1α via inhibiting K48-linked polyubiquitination-dependent proteasomal degradation (not phosphorylation)

**Fig. 7:**
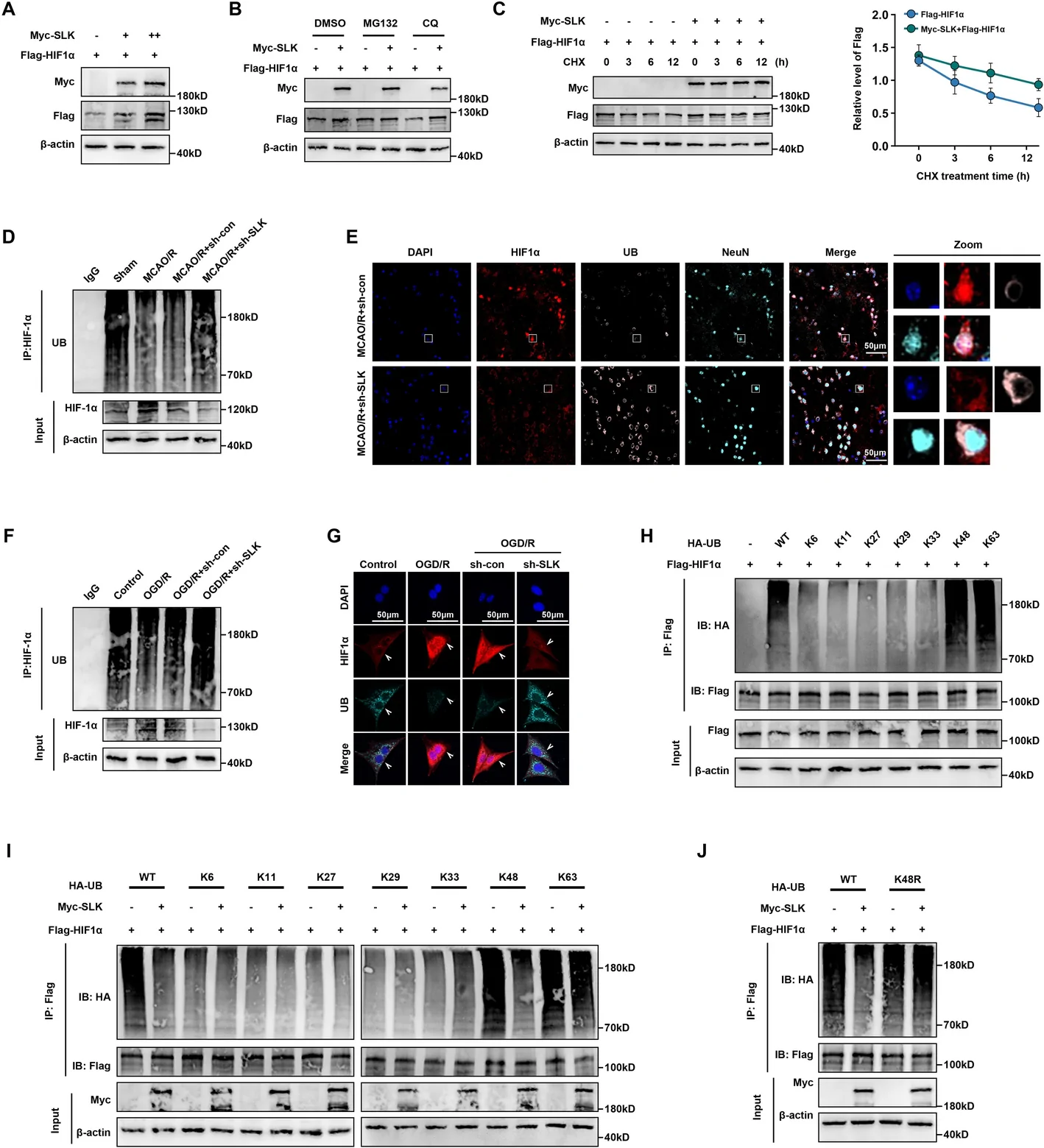
SLK stabilizes HIF-1α via inhibiting K48-linked polyubiquitination-dependent proteasomal degradation.** A** Western blot analysis of HIF-1α protein levels in 293 T cells with exogenous SLK transfection. **B** Western blot analysis of HIF-1α protein levels in 293 T cells with exogenous SLK transfection following MG132 (10 mM) or chloroquine (10 μM) treatment. **C** Cycloheximide (100 μg/mL) chase assay for HIF-1α protein stability in 293 T cells with exogenous SLK transfection. **D** HIF-1α ubiquitination assay in brain tissues from SLK-knockdown mice after MCAO/R injury. **E** Immunofluorescence staining for HIF-1α and ubiquitin in MCAO/R brain tissues of SLK-knockdown mice. **F** HIF-1α ubiquitination assay in SLK-knockdown HT22 cells following OGD/R stimulation. **G** Immunofluorescence staining for HIF-1α and ubiquitin in SLK-knockdown HT22 cells after OGD/R stimulation. **H-J** HIF-1α ubiquitination assays with exogenous ubiquitin mutant transfection in 293 T cells

To elucidate how SLK modulates HIF-1α protein abundance, it was examined whether SLK, as a serine/threonine kinase, affects HIF-1α phosphorylation. Western blot analysis of MCAO/R mouse brains and OGD/R neurons indicated that SLK knockdown did not alter serine or threonine phosphorylation of HIF-1α (Supplementary Fig. 5), excluding phosphorylation as the regulatory mechanism. The positive regulatory relationship between SLK and HIF-1α was further validated via exogenous overexpression in 293 T cells. Ectopic expression of SLK significantly increased HIF-1α protein levels (Fig. 7A), consistent with findings from the ischemic model and supporting the conclusion that SLK increases HIF-1α expression via a phosphorylation-independent mechanism. The protein degradation pathways underlying SLK-mediated stabilization of HIF-1α were then examined in 293 T cells. The ability of SLK overexpression to increase HIF-1α protein levels persisted following treatment with chloroquine (CQ), a lysosomal inhibitor, but was completely diminished by MG132, a selective proteasomal inhibitor (Fig. 7B). Cycloheximide (CHX) chase assays further confirmed that SLK overexpression significantly prolonged the half-life of HIF-1α in 293 T cells (Fig. 7C), establishing that SLK stabilizes HIF-1α via the proteasomal pathway rather than lysosomal degradation. Ubiquitination assays in MCAO/R mouse brain tissues revealed that SLK knockdown markedly increased HIF-1α polyubiquitination (Fig. 7D), which was validated by immunofluorescence showing increased colocalization of HIF-1α with ubiquitin in ischemic brain tissues (Fig. 7E). Consistent results were observed in OGD/R-treated HT22 cells, where SLK knockdown promoted HIF-1α polyubiquitination and increased HIF-1α–ubiquitin colocalization (Fig. 7F, G).

To identify the specific ubiquitination type, ubiquitination assays with ubiquitin mutants were performed in 293 T cells. SLK knockdown selectively improved K48-linked polyubiquitination of HIF-1α (Fig. 7H–J), a conserved modification targeting proteins for proteasomal degradation. These findings show that SLK stabilizes HIF-1α in a phosphorylation-independent manner by suppressing K48-linked polyubiquitination, preventing proteasomal degradation, and increasing intracellular HIF-1α levels to drive I/R injury progression.

### SLK interacts with the deubiquitinase USP8, and USP8 directly binds HIF-1α

**Fig. 8:**
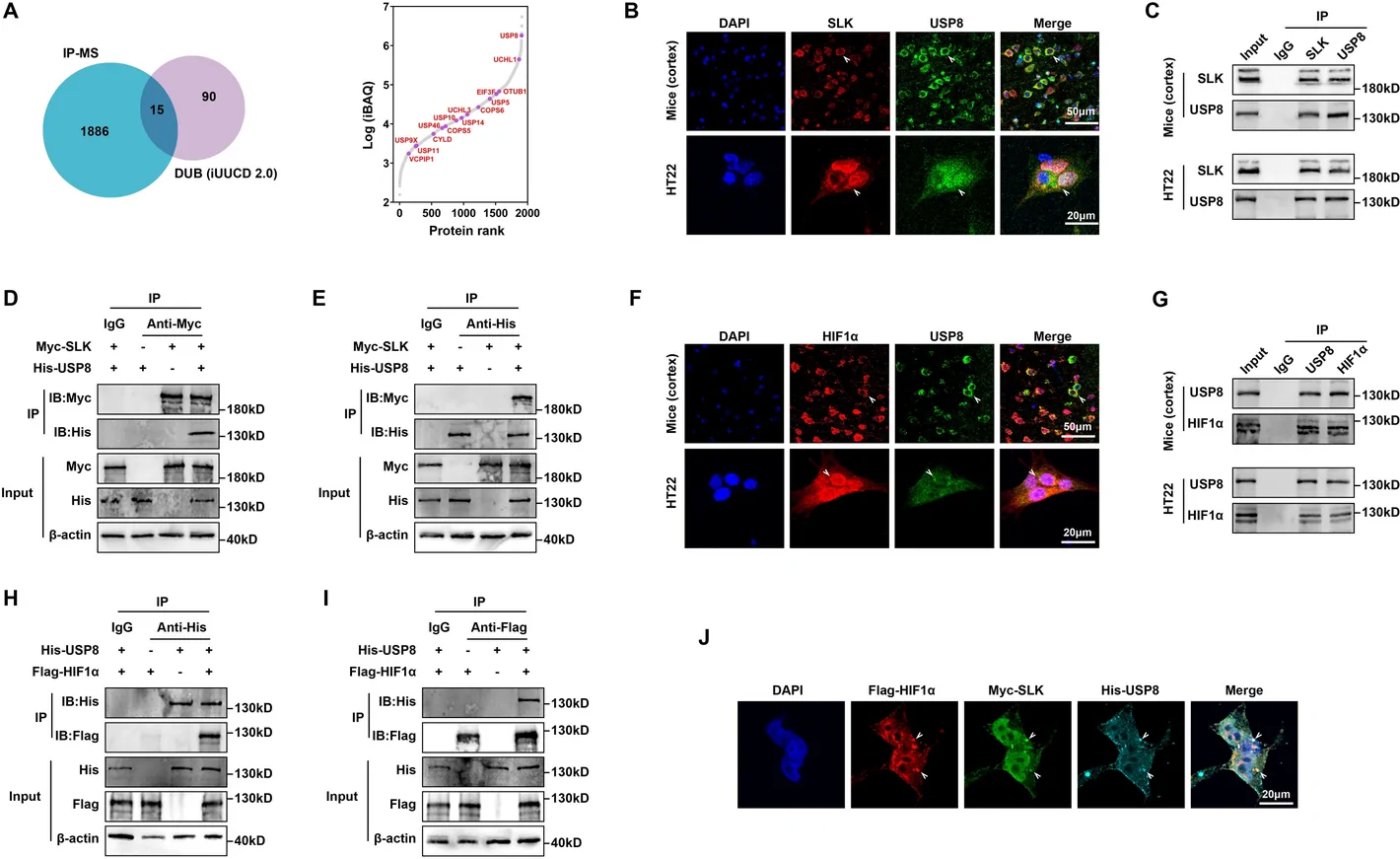
SLK interacts with the deubiquitinase USP8, and USP8 directly binds HIF-1α. **A** IP-MS analysis of SLK-interacting proteins in mouse brain tissues (left), and iBAQ abundance ranking of identified DUBs among SLK-binding proteins (right). **B** Immunofluorescence staining of SLK and USP8 in mouse cerebral cortex tissues and HT22 cells. **C** Endogenous Co-IP analysis of the interaction between SLK and USP8 in mouse cerebral cortex tissues and HT22 cells. **D** Exogenous Co-IP analysis of the interaction between Myc-tagged SLK and His-tagged USP8 in 293 T cells (IP: anti-Myc; IB: anti-His). **E** Exogenous Co-IP analysis of the interaction between Myc-tagged SLK and His-tagged USP8 in 293 T cells (IP: anti-His; IB: anti-Myc). **F** Immunofluorescence staining of USP8 and HIF-1α in mouse cerebral cortex tissues and HT22 cells. **G** Endogenous Co-IP analysis of the interaction between USP8 and HIF-1α in mouse cerebral cortex tissues and HT22 cells. **H** Exogenous Co-IP analysis of the interaction between His-tagged USP8 and Flag-tagged HIF-1α in 293 T cells (IP: anti-His; IB: anti-Flag). **I** Exogenous Co-IP analysis of the interaction between His-tagged USP8 and Flag-tagged HIF-1α in 293 T cells (IP: anti-Flag; IB: anti-His). **J** Triple immunofluorescence staining showed the clocalization of SLK, USP8, and HIF-1α in cells transfected with SLK, USP8, and HIF-1α plasmids

To elucidate the mechanism by which SLK inhibits HIF-1α ubiquitination, SLK-interacting deubiquitinases (DUBs) were screened using IP-MS in cortical tissues from MCAO/R mice. Among 1901 coprecipitated proteins, 15 were annotated as DUBs in the IUCD 2.0 database, with USP8 showing the highest iBAQ value, identifying it as the primary candidate DUB mediating SLK interaction (Fig. 8A, Supplementary Table S10). Immunofluorescence staining demonstrated strong colocalization of SLK and USP8 in mouse cortical tissues and HT22 cells (Fig. 8B), confirming their spatial proximity and potential for physical interaction. Endogenous Co-IP assays in both mouse cortical tissues and HT22 cells further verified reciprocal binding between SLK and USP8 (Fig. 8C), indicating a specific interaction in both animal and cellular models. Exogenous Co-IP experiments in 293 T cells following cotransfection with Myc-tagged SLK and His-tagged USP8 confirmed a direct physical interaction between the two proteins (Fig. 8D, E). These findings prompted further investigation into the functional relationship between USP8 and HIF-1α. Immunofluorescence staining indicated colocalization of USP8 and HIF-1α in mouse cortical tissues and HT22 cells (Fig. 8F), providing initial evidence of their potential interaction. Endogenous Co-IP assays confirmed reciprocal binding between USP8 and HIF-1α in these samples (Fig. 8G), while exogenous Co-IP in 293 T cells after cotransfection with Flag-tagged HIF-1α and His-tagged USP8 further verified a direct physical interaction (Fig. 8H, I). Triple immunofluorescence staining in 293 T cells coexpressing Myc-tagged SLK, His-tagged USP8, and Flag-tagged HIF-1α demonstrated colocalization of all three proteins within the same subcellular compartment (Fig. 8J). These results establish that SLK interacts with USP8, USP8 directly binds HIF-1α, and all three proteins colocalize intracellularly, providing a mechanistic framework to investigate SLK-mediated modulation of HIF-1α ubiquitination via USP8.

### SLK phosphorylates USP8 to facilitate USP8-mediated HIF-1α K48-linked deubiquitination

**Fig. 9:**
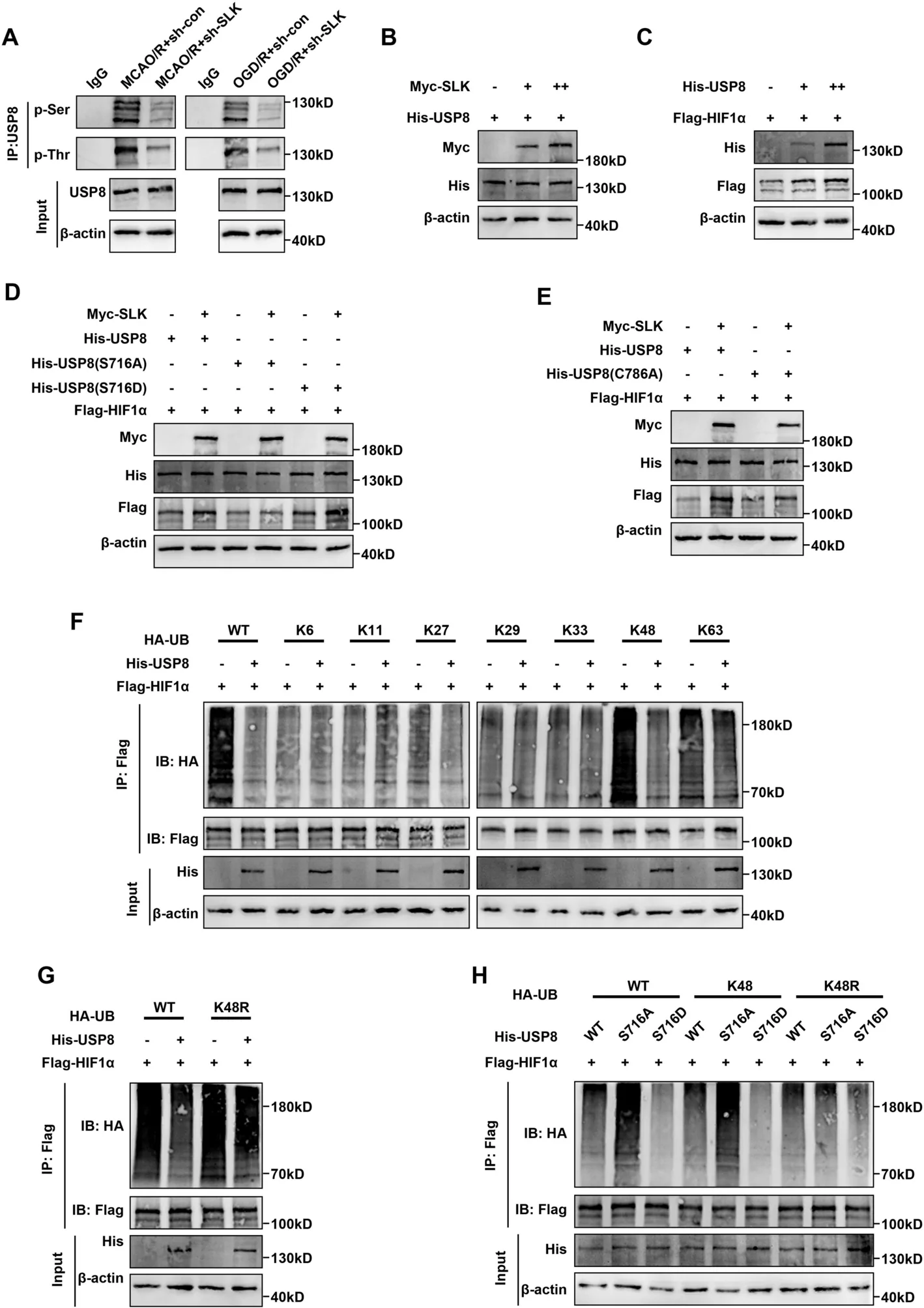
SLK phosphorylates USP8 to facilitate USP8-mediated HIF-1α K48-linked deubiquitination. **A** Immunoprecipitation and immunoblotting analysis of phosphorylated USP8 (p-Ser/p-Thr) and total USP8 in cerebral cortices of MCAO/R mice and OGD/R-treated HT22 cells with SLK knockdown or control treatment. **B** Immunoblotting analysis of USP8 in 293 T cells transfected with increasing doses of Myc-SLK and a fixed dose of His-USP8. **C** Immunoblotting analysis of Flag-HIF-1α protein levels in 293 T cells transfected with increasing doses of His-USP8 and a fixed dose of Flag-HIF-1α. **D** Immunoblotting analysis of Flag-HIF-1α protein levels in 293 T cells cotransfected with Myc-SLK, Flag-HIF-1α, and USP8 (WT/S716A/S716D). **E** Immunoblotting analysis of Flag-HIF-1α protein levels in 293 T cells cotransfected with Myc-SLK, Flag-HIF-1α, and USP8 (WT/C786A). **F** Ubiquitination assays showing the effect of USP8 overexpression on HIF-1α ubiquitination conjugated with distinct ubiquitin chain linkages (WT, K6, K11, K27, K29, K33, K48, K63). **G** Ubiquitination assays showing the effect of USP8 overexpression on HIF-1α ubiquitination conjugated with wild-type ubiquitin or K48R mutant. **H** Ubiquitination assays showing the effects of USP8 WT, S716A and S716D mutants on HIF-1α ubiquitination in the presence of K48 or K48R ubiquitin

To elucidate the mechanism by which SLK regulates HIF-1α ubiquitination through USP8, mechanistic assays were performed. USP8 phosphorylation was first assessed in the cerebral cortices of MCAO/R mouse models and in OGD/R HT22 cells. SLK knockdown markedly reduced USP8 phosphorylation without affecting total USP8 protein levels (Fig. 9A), indicating that SLK selectively promotes USP8 activation through phosphorylation. Exogenous overexpression assays in 293 T cells showed that increasing amounts of Myc-SLK did not alter the protein levels of cotransfected His-USP8 (Fig. 9B), whereas transfection with increasing doses of His-USP8 elevated HIF-1α protein levels in a dose-dependent manner (Fig. 9C). To determine whether SLK-mediated HIF-1α upregulation depends on USP8 phosphorylation and deubiquitinase activity, phosphorylation-mimetic and catalytic USP8 mutants were generated. Immunoblot analysis demonstrated that SLK strongly increased HIF-1α protein levels in the presence of phospho-mimetic USP8 (S716D) but failed to regulate HIF-1α expression with the dephosphorylation-mimetic USP8 (S716A) (Fig. 9D) or the catalytically inactive USP8 mutant (C786A) (Fig. 9E). These results reveal that SLK-induced HIF-1α upregulation requires USP8 phosphorylation and its catalytic activity. The ubiquitin linkage specificity of USP8 toward HIF-1α was further characterized using ubiquitin mutants in which individual lysine residues were substituted. Ubiquitination assays demonstrated that USP8 overexpression selectively reduced K48-linked polyubiquitination of HIF-1α, while K6-, K11-, K27-, K29-, K33-, and K63 linkages were unaffected (Fig. 9F). This selectivity was supported by the loss of USP8-dependent HIF-1α deubiquitination when the K48R ubiquitin mutant was used (Fig. 9G). Under conditions permitting K48-linked ubiquitination, the phospho-mimetic USP8 mutant (S716D) significantly decreased K48-linked polyubiquitination of HIF-1α, whereas the dephosphorylation-mimetic mutant (S716A) enhanced K48-linked polyubiquitination. By comparison, wild-type USP8 and its phosphorylation variants did not influence HIF-1α ubiquitination in the presence of K48R ubiquitin (Fig. 9H). These findings indicate that SLK-dependent phosphorylation of USP8 specifically inhibits K48-linked polyubiquitination of HIF-1α.

### USP8 overexpression reverses the neuroprotective effects of SLK knockdown in OGD/R-treated HT22 cells

**Fig. 10:**
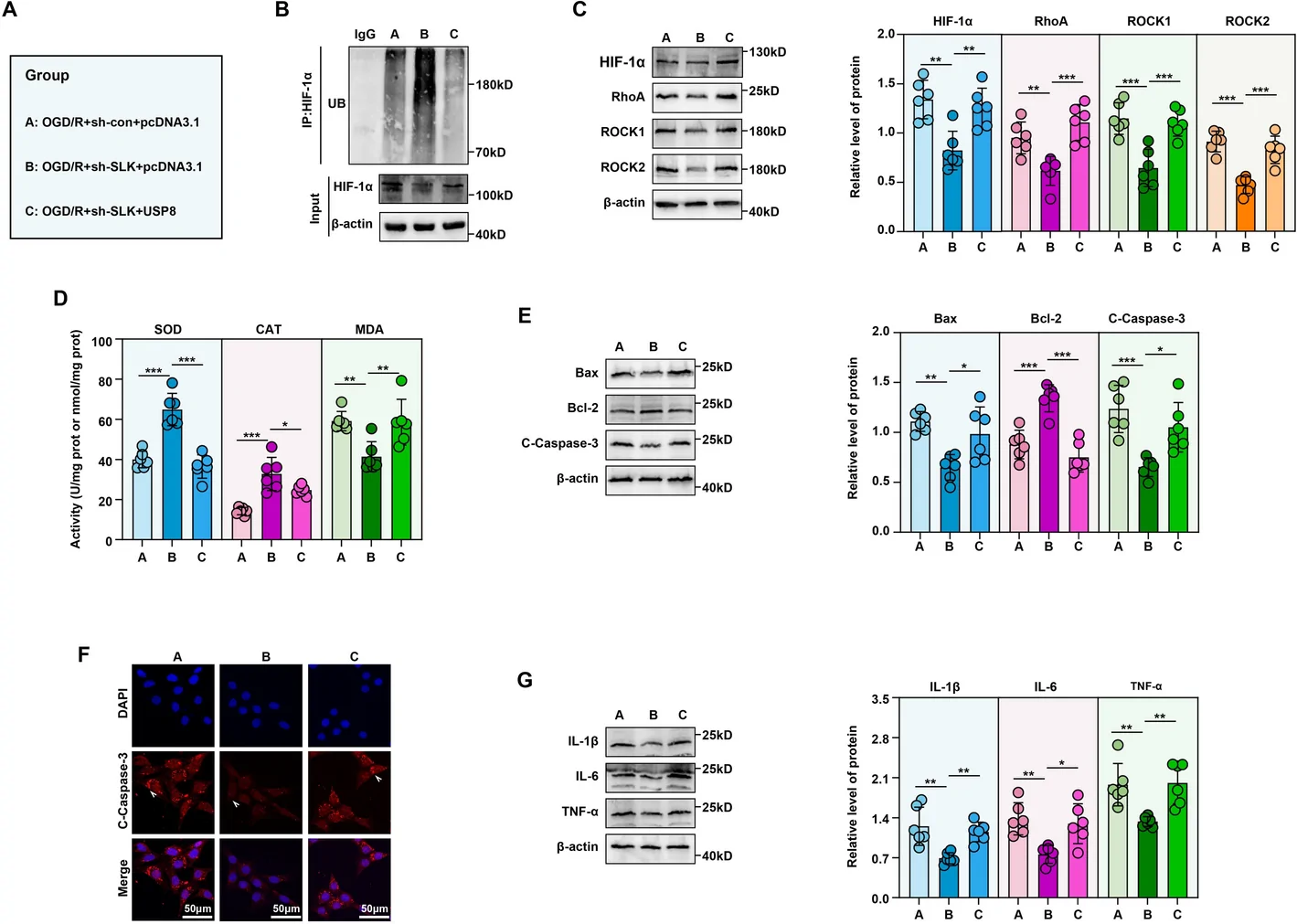
USP8 overexpression reverses the neuroprotective effects of SLK knockdown in OGD/R-treated HT22 cells. **A** Schematic diagram of the experimental grouping and design for USP8 overexpression rescue assays in SLK-knockdown HT22 cells under OGD/R conditions. **B** Detection of HIF-1α ubiquitination levels in each group by ubiquitination assay. **C** Immunoblotting analysis of HIF-1α, RhoA, ROCK1, and ROCK2 protein levels in each group. **D** Detection of SOD, CAT, and MDA levels to assess oxidative stress in each group. **E** Immunoblotting analysis of apoptotic-related proteins Bax, cleaved-caspase-3 and Bcl-2 in each group. **F** Fluorescence staining analysis of cleaved-caspase-3 fluorescence intensity in each group. **G** Immunoblotting analysis of proinflammatory cytokines IL-1β, IL-6 and TNF-α protein levels in each group. *n* = 6/per group, *P < 0.05, **P < 0.01, ***P < 0.001

To define the contribution of USP8 to SLK knockdown-mediated neuroprotection, USP8 overexpression assays were performed in OGD/R HT22 cells (Fig. 10A). SLK knockdown markedly reduced HIF-1α ubiquitination, whereas USP8 overexpression restored the suppressed ubiquitination of HIF-1α (Fig. 10B). Immunoblotting showed that SLK knockdown reduced the levels of HIF-1α, RhoA, ROCK1, and ROCK2 proteins, and these changes were reversed upon USP8 overexpression (Fig. 10C). Assessment of oxidative stress revealed that SLK knockdown enhanced SOD and CAT activities and reduced MDA levels, while USP8 overexpression attenuated these antioxidant responses (Fig. 10D). SLK knockdown reduced the levels of cleaved-caspase-3 and Bax, while raising those of Bcl-2, whereas USP8 overexpression counteracted these effects (Fig. 10E). Moreover, cleaved-caspase-3 fluorescence staining demonstrated reduced apoptotic signaling following SLK knockdown, which was reversed by USP8 overexpression (Fig. 10F). The impact of USP8 overexpression on inflammatory signaling was also evaluated. SLK knockdown markedly lowered the levels of IL-1β, IL-6, and TNF-α, while USP8 overexpression restored them (Fig. 10G). These results indicate that SLK knockdown confers neuroprotection in OGD/R-treated HT22 cells, and this protective phenotype is reversed by USP8 overexpression.

## Discussion

AIS continues to be a leading contributor to mortality and chronic neurological disability globally, with I/R injury representing the central pathological process driving secondary neuronal damage and unfavorable clinical outcomes [21, 22]. Although recanalization therapies have improved cerebral blood flow restoration, effective therapeutic targets and reliable biomarkers remain limited. In this study, MR analysis was applied to identify genes with causal associations to AIS risk. Integration of these results with differential expression analysis from merged AIS transcriptomic datasets yielded 25 druggable genes showing both genetic causality and aberrant expression in AIS, among which SLK was prioritized through Bayesian colocalization analysis. Genetic findings were further validated in a clinical cohort, where plasma SLK levels were elevated in AIS cases and correlated with poor 90-day outcomes. These data identify SLK as a causal, druggable biomarker linking genetic risk to clinical prognosis.

I/R injury involves a complex pathological cascade characterized by excessive ROS generation, oxidative stress, mitochondrial dysfunction, inflammatory responses, and activation of multiple cell death pathways [3, 23, 24]. These interrelated processes reinforce one another, ultimately resulting in irreversible neuronal damage and persistent neurological deficits [25, 26]. Our functional studies in MCAO/R mice and OGD/R cells showed that SLK knockdown reduced infarct size and alleviated oxidative stress, apoptosis, and neuroinflammation. These findings are consistent with previously reported proinflammatory and proapoptotic roles of SLK in other pathological contexts [27–29]. Notably, SLK was first identified as a stress kinase involved in renal I/R injury approximately two decades ago, where it promotes cell death through p38 and JNK1-dependent signaling pathways [27, 30, 31]. SLK has also been shown to stimulate the disassembly of actin stress fibers and focal adhesions and induce apoptosis [32]. These established pathways collectively support the proapoptotic and proischemic function of SLK. Our present findings extend the pathological role of SLK to cerebral I/R injury, demonstrating that SLK knockdown confers neuroprotection by attenuating oxidative stress, apoptosis, and neuroinflammation.

To clarify the molecular basis of SLK-driven neuronal injury, we analyzed SLK-correlated genes in AIS cases. Clustering and enrichment revealed enrichment in ROS metabolism and apoptosis, consistent with our functional findings. PPI network analysis highlighted HIF-1α and RhoA as key nodes among SLK-correlated genes, and following enrichment analysis indicated significant involvement of the GTPase signaling pathway, a regulatory pathway central to cell survival, cytoskeletal dynamics, and inflammatory signaling [33–35]. These results defined the HIF-1α–RhoA/ROCK axis as a major SLK downstream effector. As a key regulator of hypoxic adaptation, HIF-1α exerts context-dependent effects in cerebral ischemia [36, 37]. During the acute phase, aberrant HIF-1α stabilization promotes ROS accumulation, inflammation, and neuronal apoptosis [37]. Accordingly, pharmacological inhibition of excessive HIF‑1α activation during acute stroke, using HIF-1α inhibitors such as 2-methoxyestradiol [38] and YC1 [39], has been shown to preserve blood‑brain barrier integrity, reduce infarct volume, and alleviate brain edema. Conversely, HIF-1α can be targeted for organ protection through induction of its protective target genes, including adenosine receptors, under ischemic or hypoxic conditions [40–43]. Consistent with this concept, some HIF-1α activators, such as folic acid [44] and vadadustat [45, 46], have been investigated for the treatment of ischemia or hypoxia-related diseases.

RhoA, a small GTP-binding protein identified as an SLK-associated gene through PPI analysis, shifts between active (GTP-bound) and inactive (GDP-bound) states [47, 48]. Under hypoxic stress, HIF-1α functions upstream to facilitate RhoA activation by promoting GDP–GTP exchange [49, 50]. Upon activation, RhoA directly engages and stimulates its downstream effector ROCK, a serine/threonine kinase comprising two isoforms, ROCK1 and ROCK2 [34]. Activation of the RhoA/ROCK signaling cascade aggravates cerebral I/R injury by modulating actin cytoskeletal dynamics, facilitating neuronal apoptosis, and amplifying microglial activation and neuroinflammatory responses, establishing this pathway as a well-recognized therapeutic target in AIS [34, 51–53]. Consistent with this framework, in this study, SLK knockdown markedly reduced the levels of HIF-1α, RhoA, ROCK1, and ROCK2 proteins in both animal and cellular I/R models, supporting a direct regulatory relationship between SLK and this deleterious signaling pathway. These data introduce SLK as a previously unrecognized upstream kinase regulator of the HIF-1α–RhoA/ROCK pathway in cerebral ischemia. Furthermore, emerging evidence indicates that HIF‑1α may contribute to ischemic brain injury partly by modulating NLRP3 inflammasome activity and downstream pyroptotic signaling [54]. Pyroptosis is recognized as a proinflammatory form of regulated cell death that exacerbates neuronal loss and neuroinflammation during cerebral I/R injury. Given that SLK knockdown significantly reduced HIF‑1α accumulation in our models, it is plausible that the SLK–USP8–HIF‑1α pathway might indirectly influence neuroinflammatory and cell death processes by intersecting with NLRP3‑related signaling. This potential crosstalk expands our understanding of the neuroprotective mechanisms mediated by SLK inhibition. Notably, our study was designed to explore the acute pathogenic mechanism underlying neuronal damage during cerebral I/R injury. Accordingly, the SLK–USP8–HIF‑1α–RhoA/ROCK pathway identified herein mainly mediates acute phase injury, indicating that targeting this axis primarily intervenes in the acute pathological process of ischemic stroke. Further investigations will be needed to explore its potential roles in the chronic phase and long‑term functional recovery.

Post-translational modifications are critical determinants of HIF-1α stability, activity, and expression. Modifications such as phosphorylation, ubiquitination, acetylation, and methylation extensively regulate HIF-1α subunits, influencing their enzymatic activity, subcellular localization, stability, and protein–protein interactions [55]. Although SLK is a kinase, our results show it does not phosphorylate HIF-1α, indicating an alternative regulatory mechanism. Given that the ubiquitin–proteasome system governs HIF-1α turnover [56, 57], the role of SLK in regulating HIF-1α stability through UPS-dependent proteasomal degradation was examined in this study. SLK knockdown increased HIF-1α degradation by increasing K48-linked polyubiquitination, uncovering an oxygen-independent regulatory mechanism distinct from canonical pathways such as miRNA-mediated repression during cerebral I/R injury [58–60]. Ubiquitination is dynamically regulated by deubiquitinases (DUBs), with over 100 human DUBs grouped into six families, including ubiquitin-specific proteases, motif interacting with novel DUBs, proteases from ovarian tumors and Machado–Joseph disease, ubiquitin C-terminal hydrolases, and zinc finger USPs [61]. Owing to the complexity and tight regulation of ubiquitination–deubiquitination dynamics, HIF-1α is subject to selective deubiquitination by distinct DUBs across various pathological contexts [62]. Previous studies have reported that USP14 stabilizes HIF-1α via deubiquitination in hepatocellular carcinoma [63], while UCHL1 has been shown to promote metastasis by functioning as a deubiquitinating enzyme for HIF-1α [64]. In the present study, USP8 appears to act as a key mediator of SLK-driven HIF-1α stabilization, promoting K48-linked deubiquitination and enhancing HIF-1α protein stability, which is consistent with previous findings that USP8 mediates HIF-1α deubiquitination under normoxic conditions [65].

Protein phosphorylation represents a central mechanism controlling DUB activity [66, 67]. As a serine/threonine kinase, SLK interacts with and phosphorylates USP8, enhancing its catalytic activity. Functional assays confirmed that SLK-mediated HIF-1α stabilization requires both USP8 phosphorylation at S716 and intact deubiquitinating function. USP8 overexpression abolished the neuroprotective effects of SLK knockdown, restored HIF-1α accumulation, and reactivated RhoA/ROCK signaling. These findings define a coherent regulatory cascade in which SLK phosphorylates USP8, enabling USP8-mediated K48-linked deubiquitination and stabilization of HIF-1α. Stabilized HIF-1α then activates the RhoA/ROCK pathway, promoting oxidative stress, apoptosis, and neuroinflammation, aggravating cerebral I/R injury.

This work demonstrates several key strengths. An integrative multi-omics framework was employed to systematically identify causal and druggable genes associated with AIS, providing a strong genetic and transcriptomic basis for prioritizing SLK. Clinical evidence was tightly aligned with functional validation using both MCAO/R model mice and OGD/R-treated HT22 cells, enabling a coherent linkage between genetic risk, clinical manifestations, and molecular mechanisms. Mechanistic analyses were conducted in depth, encompassing identification of the central HIF-1α–RhoA/ROCK axis and identification of a previously uncharacterized SLK–USP8–dependent post-translational regulatory mechanism. Consistent outcomes observed across independent experimental systems further support the reproducibility of the data and reinforce the overall reliability of the conclusions.

Although the study represents several strengths, certain limitations warrant consideration. First, our gene screening, animal experiments, and mechanistic studies were conducted using young adult mice and public genomic/transcriptomic databases that do not fully account for the critical effects of aging and age-related comorbidities. As emphasized in previous studies, aging is the primary nonmodifiable risk factor for cerebral ischemia, and comorbidities significantly worsen stroke outcomes; most preclinical stroke models overlook these factors [68], which may limit the direct translational potential of our findings to elderly, comorbid clinical populations. Second, the clinical validation relied on a single patient cohort, which may restrict the external applicability of the diagnostic and prognostic relevance of plasma SLK. Validation across multi-center, longitudinal studies incorporating larger and more heterogeneous AIS populations is required to strengthen the robustness of these observations. Third, the functional investigations were mainly performed in neuronal models, whereas cerebral I/R injury involves complex interactions among multiple cell types, including endothelial cells, pericytes, microglia, astrocytes, and oligodendrocytes. Further cell-type-resolved studies are needed to clarify the specific role of SLK in the ischemic brain microenvironment. Fourth, shRNA-mediated knockdown was used to silence SLK in our study; conditional knockout models would help to further elucidate the cell-specific and temporal roles of SLK in the pathogenesis of AIS. Notably, SLK has been previously reported to induce apoptosis via JNK1 signaling and regulate cytoskeletal dynamics through ROCK activation [27]. Our study identifies a novel SLK–USP8–HIF-1α cascade in cerebral I/R injury; however, we did not examine whether JNK1 is also involved in SLK-mediated neuronal injury. Further work is needed to clarify the potential interplay between these pathways.

## Conclusions

An integrative framework combining multi-omics analyses, clinical validation, and experimental approaches identifies SLK as a causal, druggable, and clinically informative biomarker for AIS. Mechanistic evidence identifies a previously unrecognized signaling cascade in which SLK phosphorylates USP8, enabling K48-linked deubiquitination and stabilization of HIF-1α, activating the RhoA/ROCK pathway to promote oxidative stress, apoptosis, and neuroinflammatory responses during cerebral I/R injury. The SLK–USP8–HIF-1α–RhoA/ROCK pathway is therefore defined as a key regulator of ischemia-induced neuronal damage. These findings advance current understanding of AIS pathophysiology and highlight translational opportunities for improving diagnostic accuracy, prognostic stratification, and therapeutic intervention strategies.

## Supplementary Information

Additional file 1.

Additional file 2.

Additional file 3.

Additional file 4.

Additional file 5.

Additional file 6.

Additional file 7.

## Data Availability

The cis-eQTL data for blood samples corresponding to druggable genes were extracted from the eQTLGen database (www.eqtlgen.org). AIS genetic association cohort were acquired from the GWAS Catalog (https://gwas.mrcieu.ac.uk) with the accession number ebi-a-GCST90018864 and the R12 version of the Finnish database (https://r12.finngen.fi/) with the accession name “Stroke, excluding SAH.” The public RNA-seq dataset used in this study is available in the GEO database under the accession code GSE16561, GSE22255, and GSE58294. The IP-MS data are available within the Supplementary Tables. All other data are available from the corresponding authors upon request.

## Abbreviations

AIS: Acute ischemic stroke
CIRI: Cerebral ischemia–reperfusion injury
RCTs: Randomized controlled trials
MR: Mendelian randomization
SNPs: Single nucleotide polymorphisms
GWAS: Genome-wide association study
Cis-eQTL: Cis-expression quantitative trait loci
SLK: STE20-like kinase
RhoA: Ras homolog family member A
ROCK: Rho-associated coiled-coil protein kinase
USP8: Ubiquitin-specific protease 8
HIF-1α: Hypoxia-inducible factor-1α
LD: Low linkage disequilibrium
IVW: Inverse variance-weighted
PPH4: Posterior probability of hypothesis 4
Stepglm: Stepwise generalized linear model
RF: Random forest
GlmBoost: Generalized linear model boosting
SVW: Support vector machines
Enet: Elastic net regression
GBM: Gradient boosting machines
XGBoost: EXtreme gradient boosting
PlsRglm: Partial least squares regression for generalized linear model
LDA: Linear discriminant analysis
NaiveBayes: Naive Bayes classifiers
CDF: Cumulative distribution function
PAC: Proportion of ambiguously clustered pairs
GO: Gene ontology
PPI: Protein–protein interaction
DSA: Digital subtraction angiography
MCAO/R: Middle cerebral artery occlusion/reperfusion
ECA: External carotid artery
CCA: Common carotid artery
ICA: Internal carotid artery
MCA: Middle cerebral artery
TTC: 2,3,5-Triphenyltetrazolium chloride
mNSS: Modified neurological severity score
OGD/R: Oxygen–glucose deprivation/reperfusion
Co-IP: Co-immunoprecipitation
IP-MS: Immunoprecipitation coupled with mass spectrometry
iBAQ: Intensity-based absolute quantification
ROS: Reactive oxygen species
SOD: Superoxide dismutase
CAT: Catalase
MDA: Malondialdehyde
IQR: Interquartile range
DUBs: Deubiquitinases
