# Supplementary material for: Targeting SLK protects against cerebral ischemia–reperfusion injury by regulating USP8-mediated HIF-1α stabilization and RhoA/ROCK activation
Source: Cell Mol Biol Lett. 2026 Jul 1;31:154. doi: 10.1186/s11658-026-00988-8 (PMC13625398; doi:10.1186/s11658-026-00988-8)
Supplement: Supplementary file 1 [file 11658_2026_988_MOESM7_ESM.pdf]

Figure 4

Figure 4B

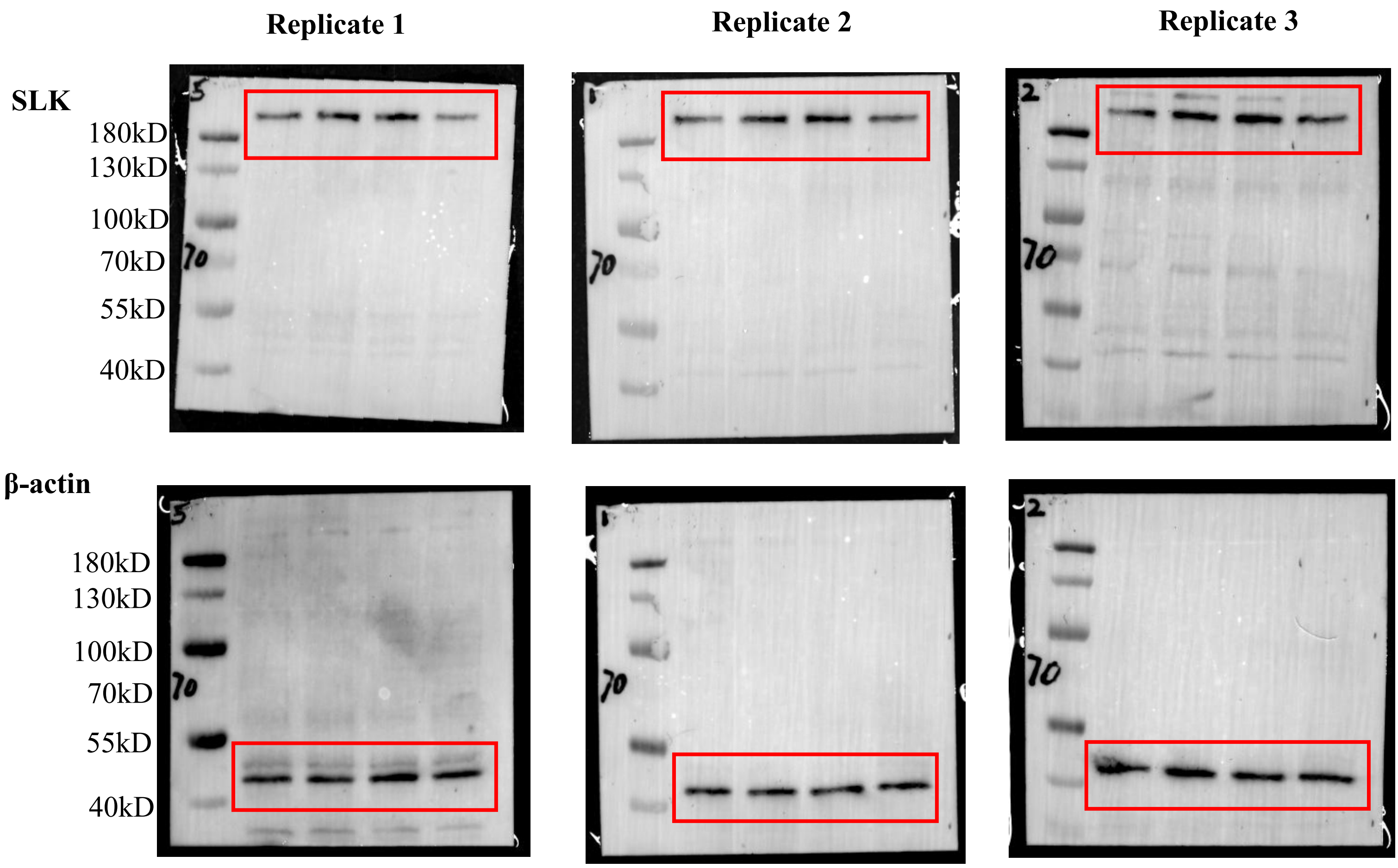

Figure 4E

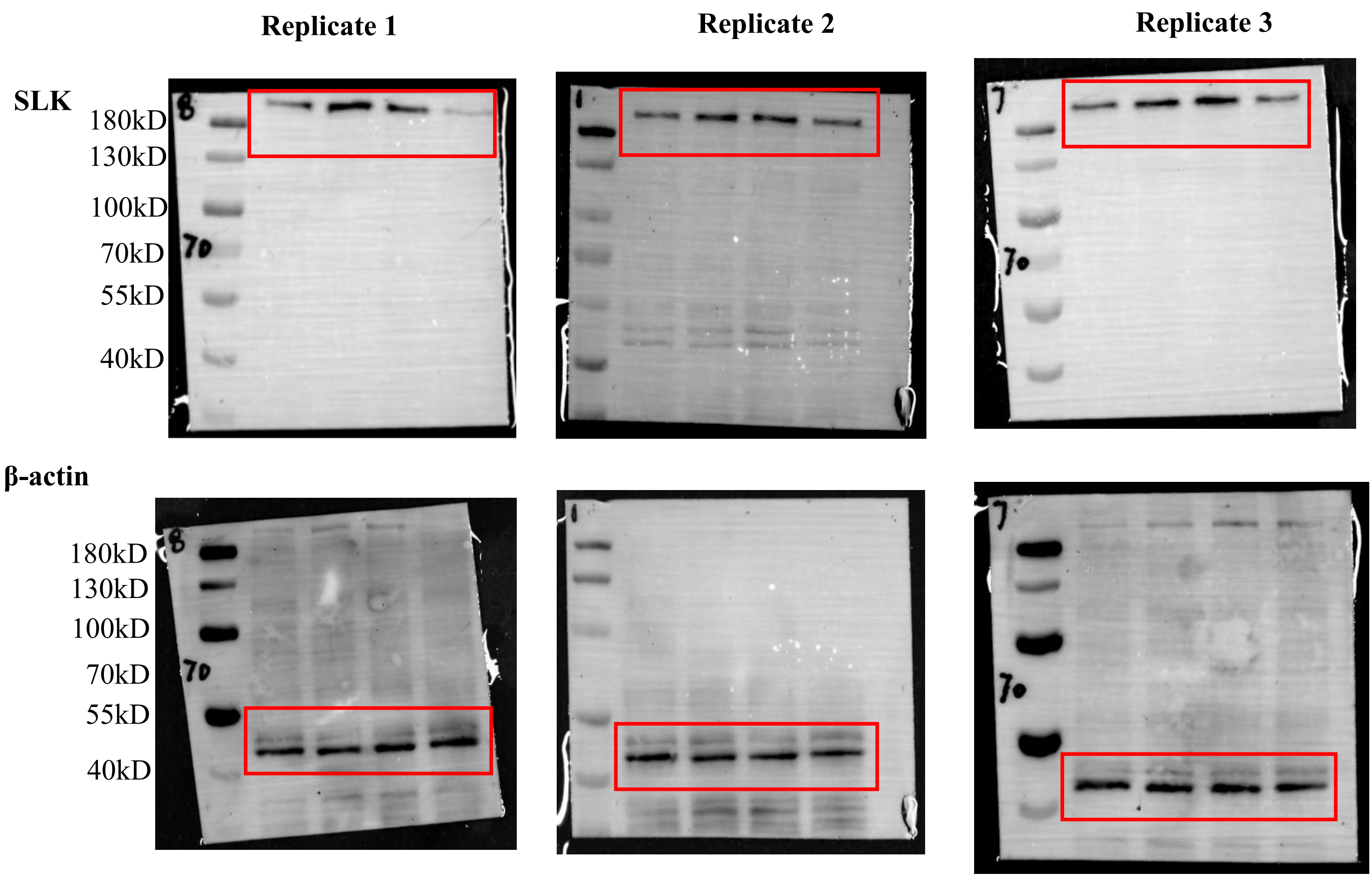

Replicate 1
Replicate 2
Replicate 3
SLK
180kD
130kD
100kD
70kD
55kD
40kD
β-actin
180kD
130kD
100kD
70kD
55kD
40kD

Figure 5

Figure 5G

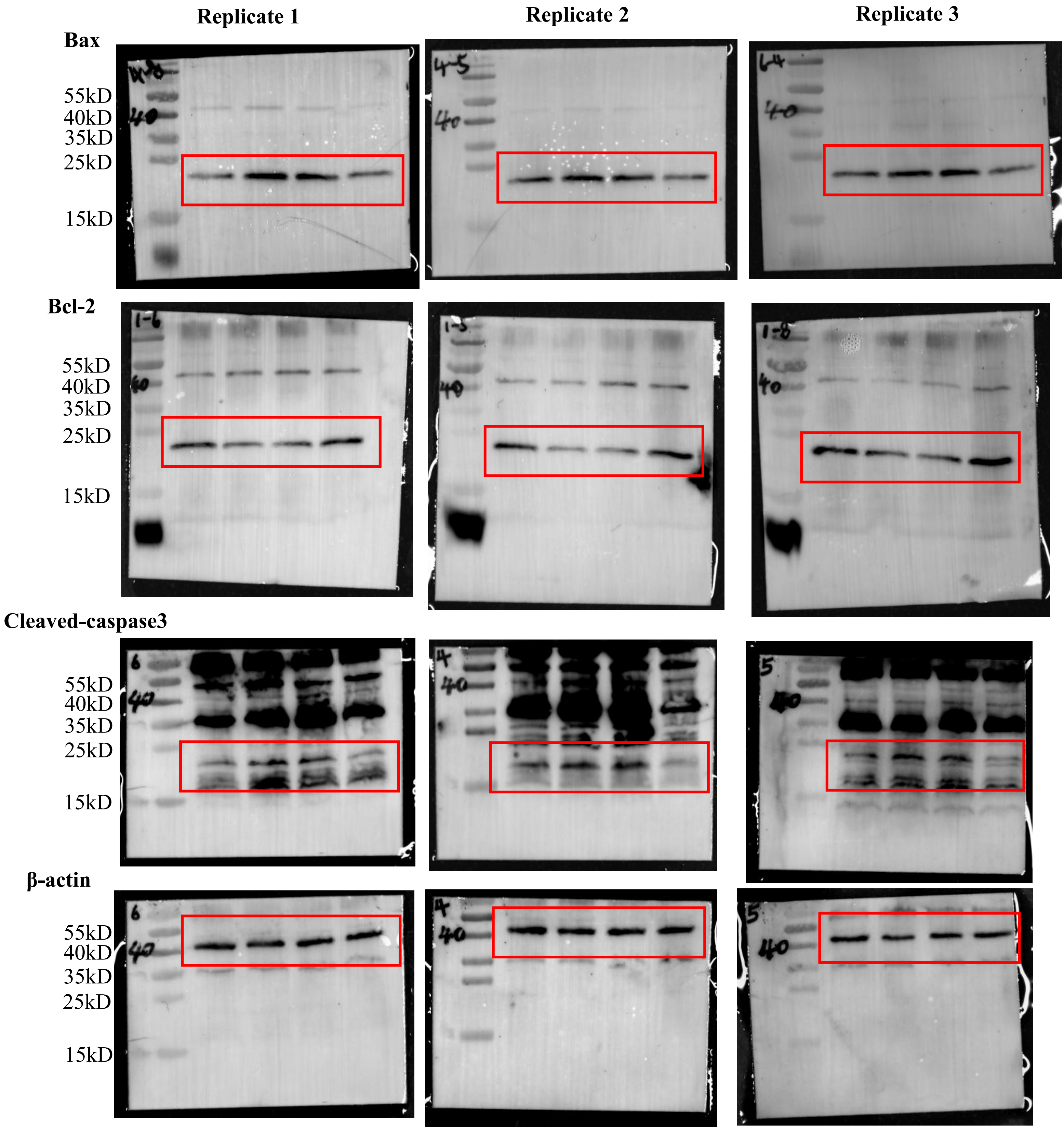

Figure 5H

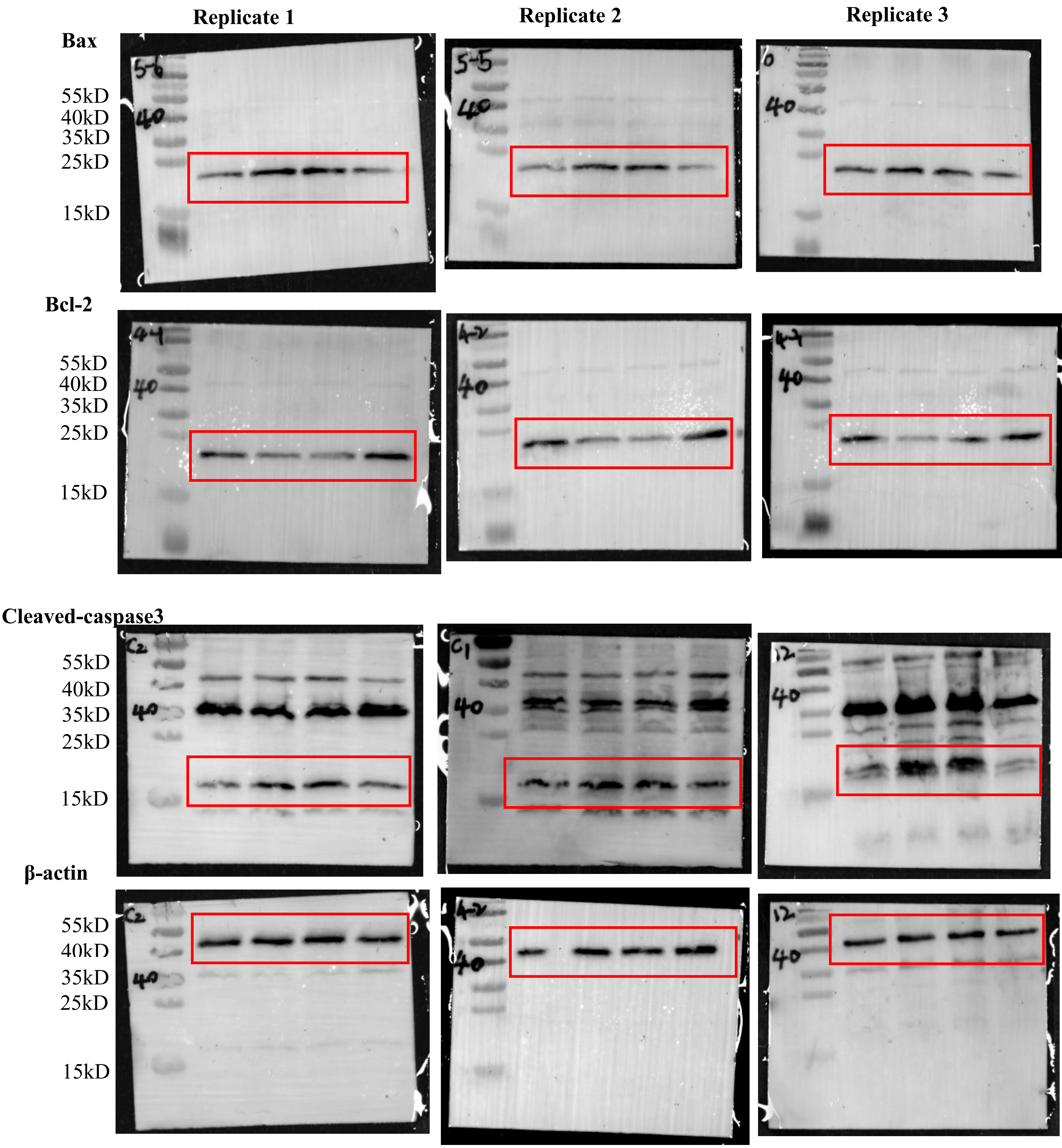

Replicate 1
Replicate 2
Replicate 3
Bax
55kD
40kD
35kD
25kD
15kD
Bcl-2
55kD
40kD
35kD
25kD
15kD
Cleaved-caspase3
55kD
40kD
35kD
25kD
15kD
β-actin
55kD
40kD
35kD
25kD
15kD

Figure 5I

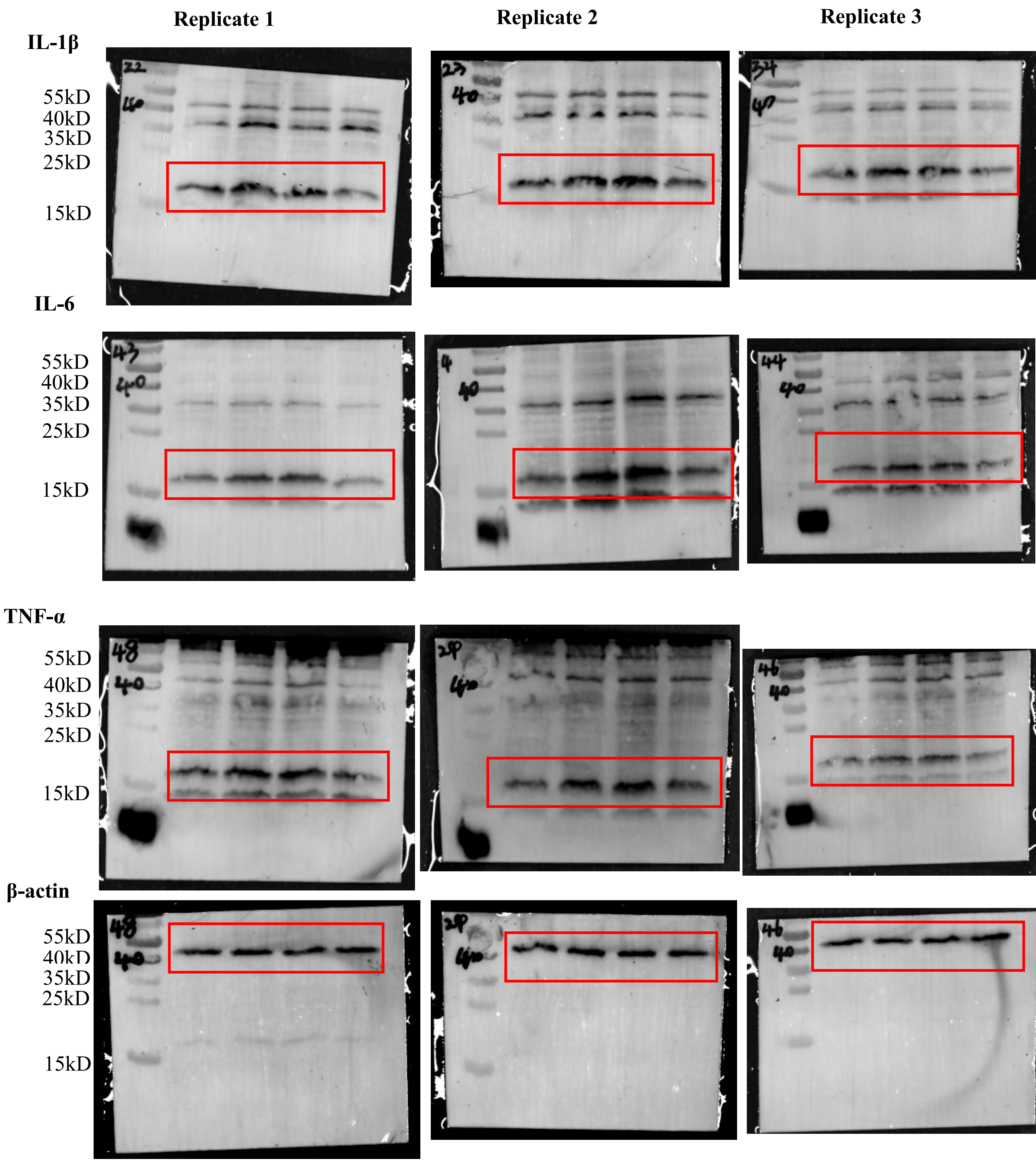

Replicate 1
Replicate 2
Replicate 3
IL-1β
55kD
40kD
35kD
25kD
15kD
IL-6
55kD
40kD
35kD
25kD
15kD
TNF-α
55kD
40kD
35kD
25kD
15kD
β-actin
55kD
40kD
35kD
25kD
15kD

Figure 5J

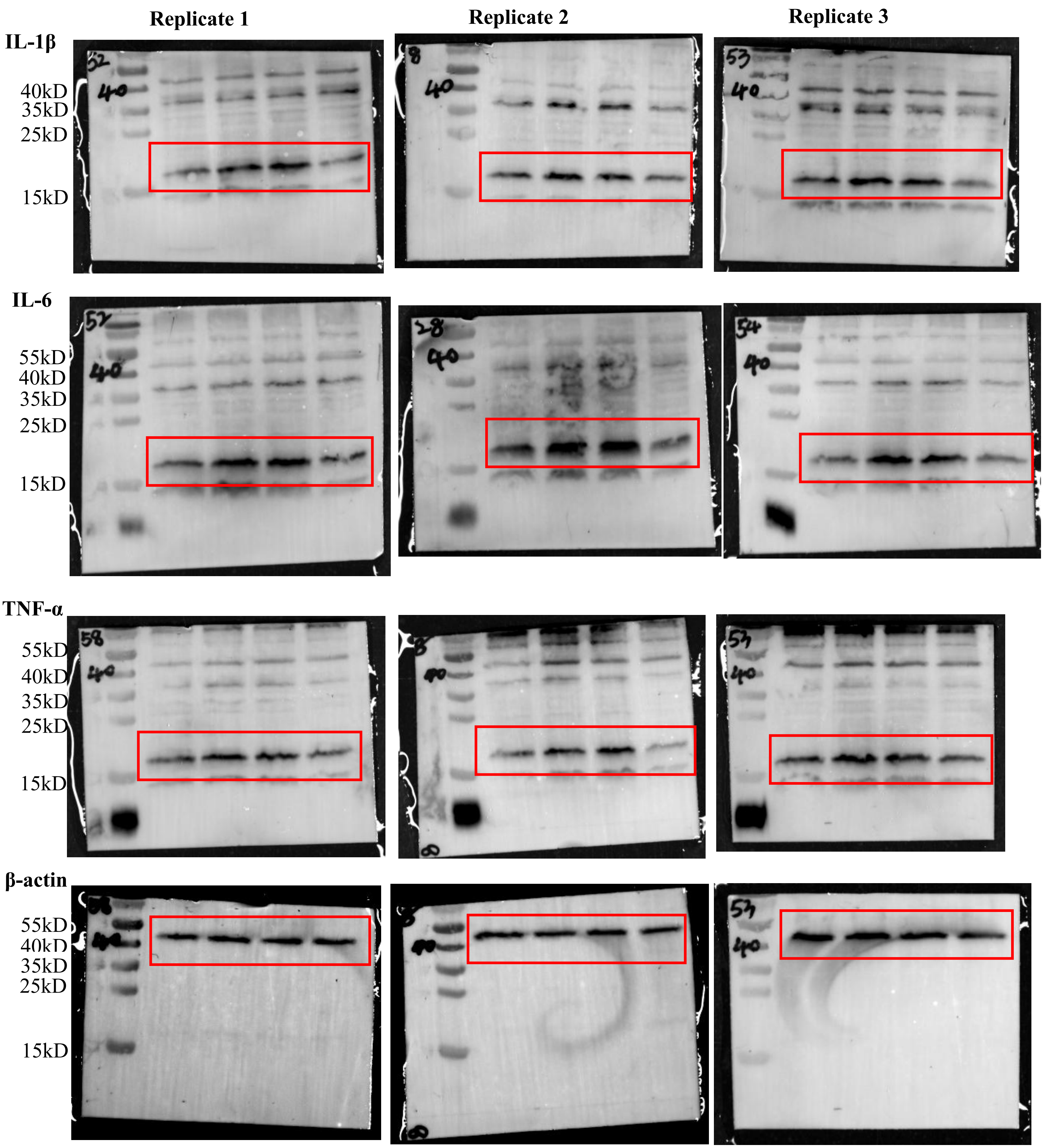
Replicate 1
Replicate 2
Replicate 3
IL-1β
40kD
35kD
25kD
15kD
IL-6
55kD
40kD
35kD
25kD
15kD
TNF-α
55kD
40kD
35kD
25kD
15kD
β-actin
55kD
40kD
35kD
25kD
15kD

Figure 6

Figure 6E (left)

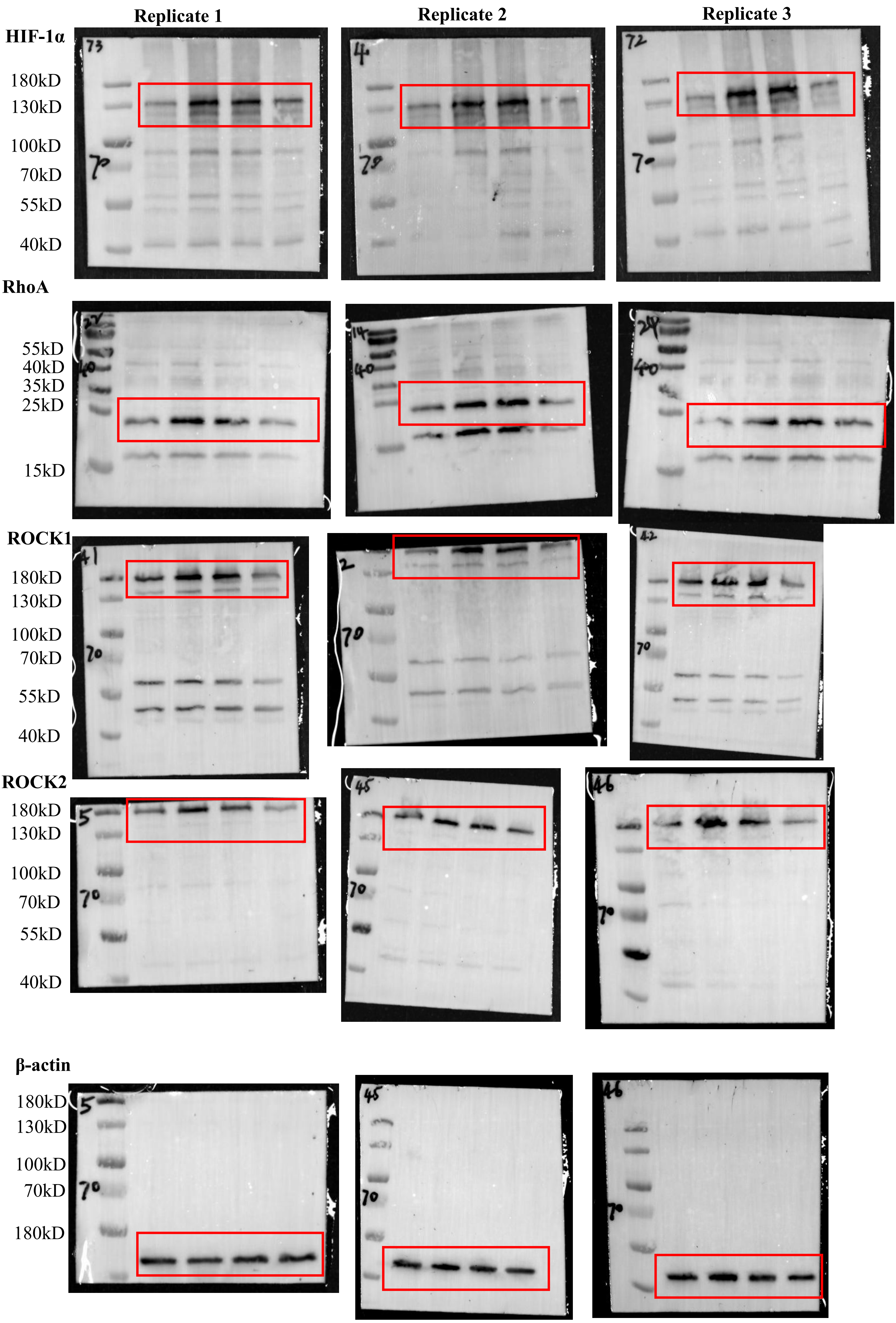

Figure 6E (right)

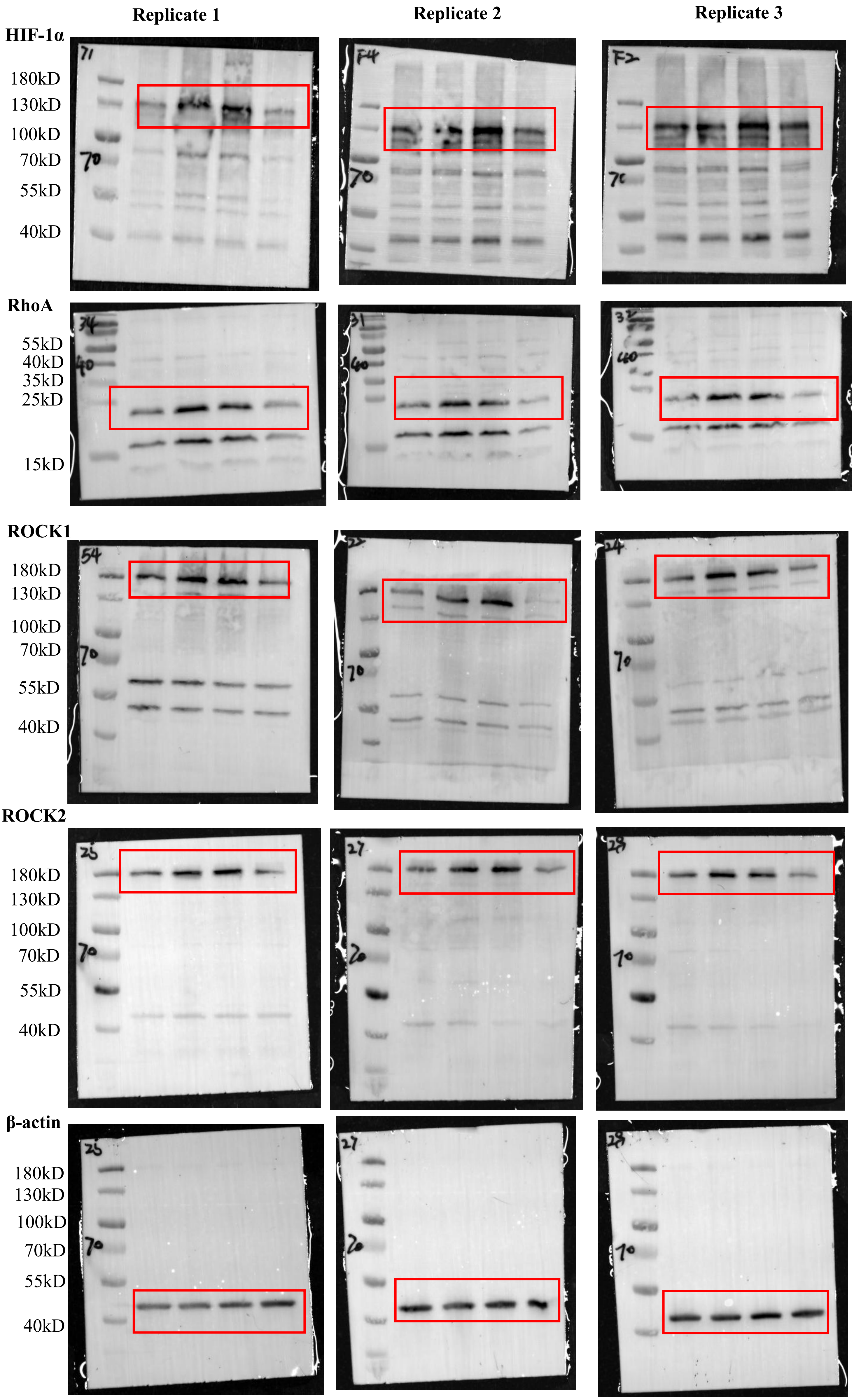

Figure 7

Figure 7A

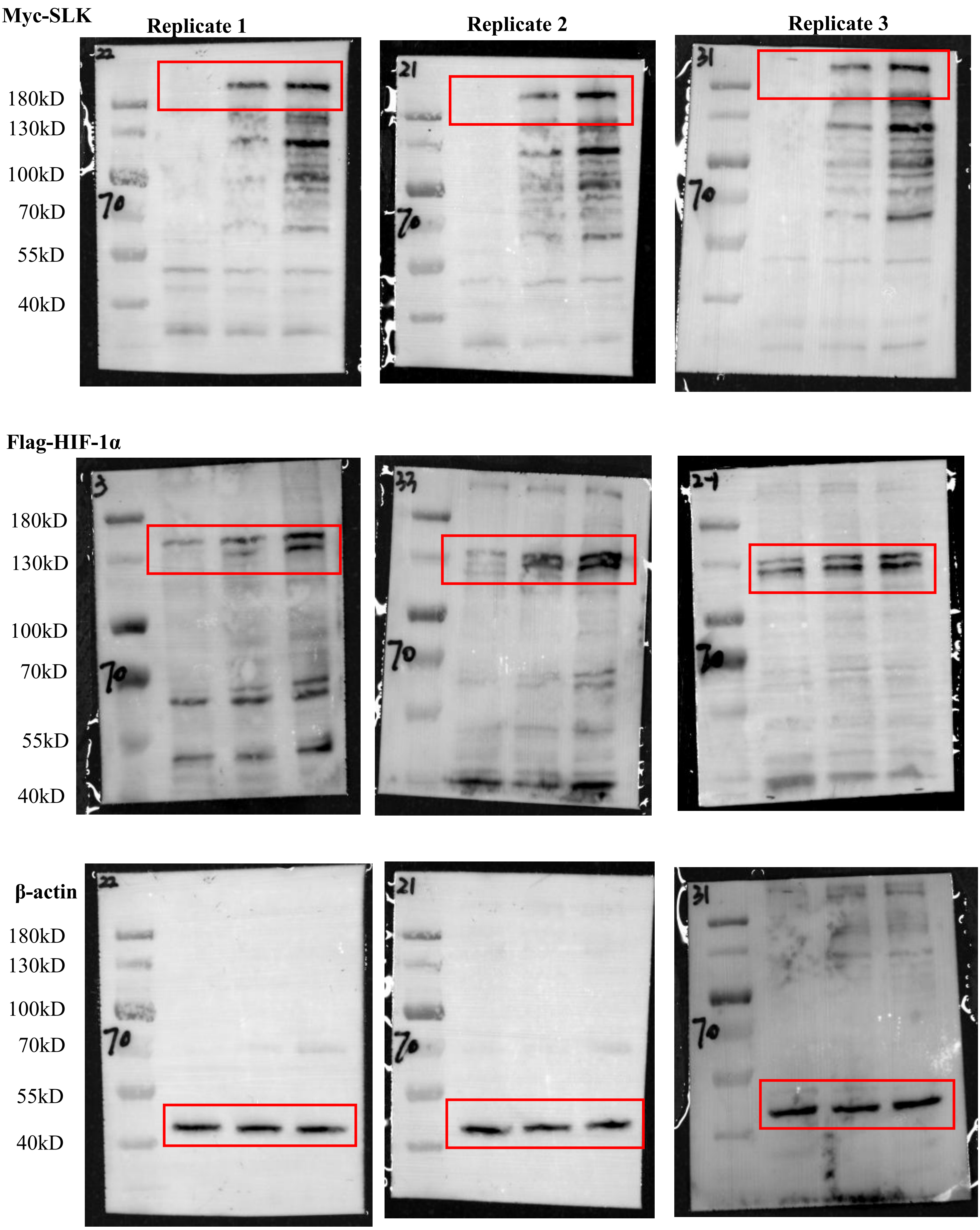

Figure 7B

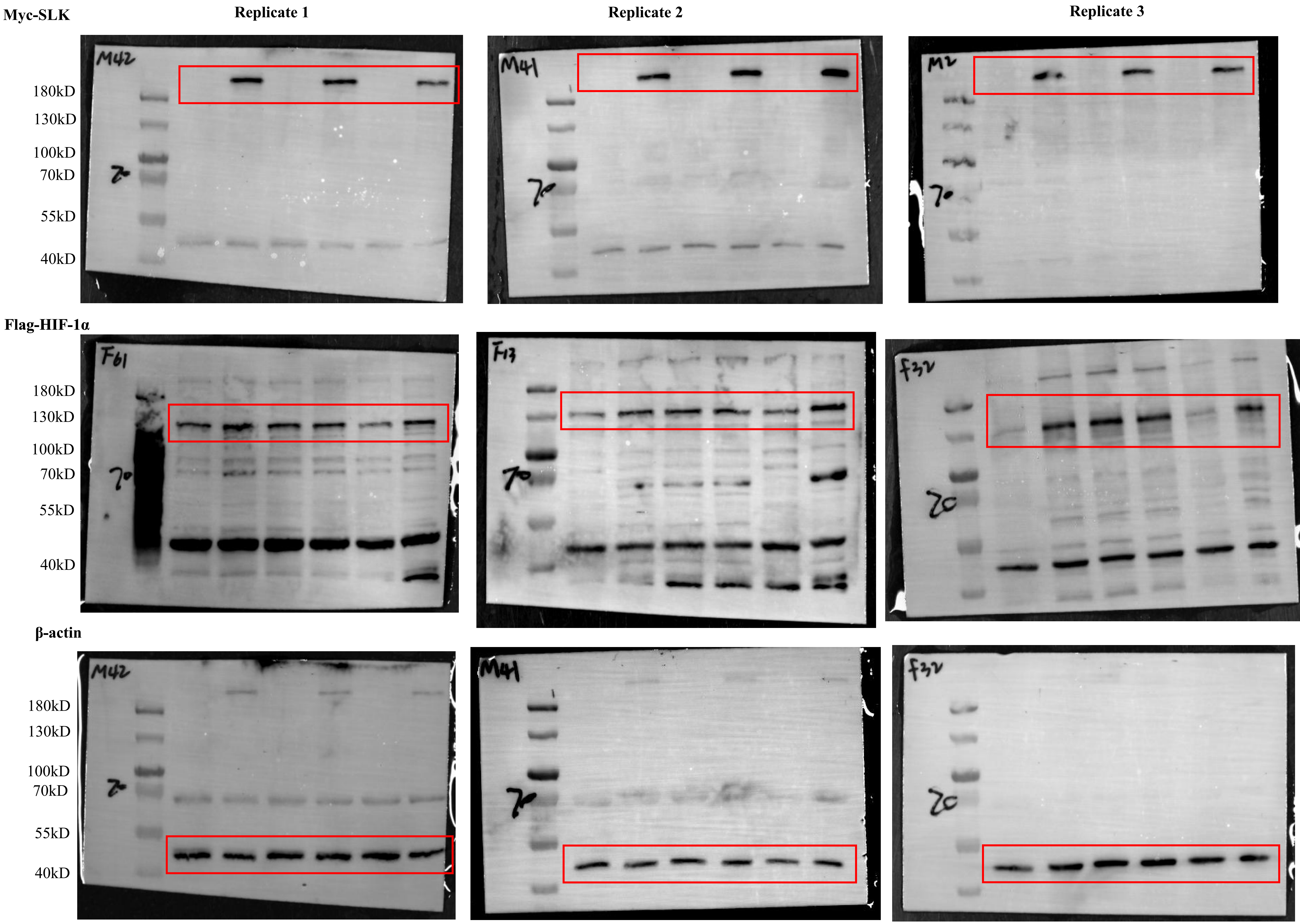
Myc-SLK
Replicate 1
Replicate 2
Replicate 3
180kD
130kD
100kD
70kD
55kD
40kD
Flag-HIF-1α
180kD
130kD
100kD
70kD
55kD
40kD
β-actin
180kD
130kD
100kD
70kD
55kD
40kD

Figure 7C

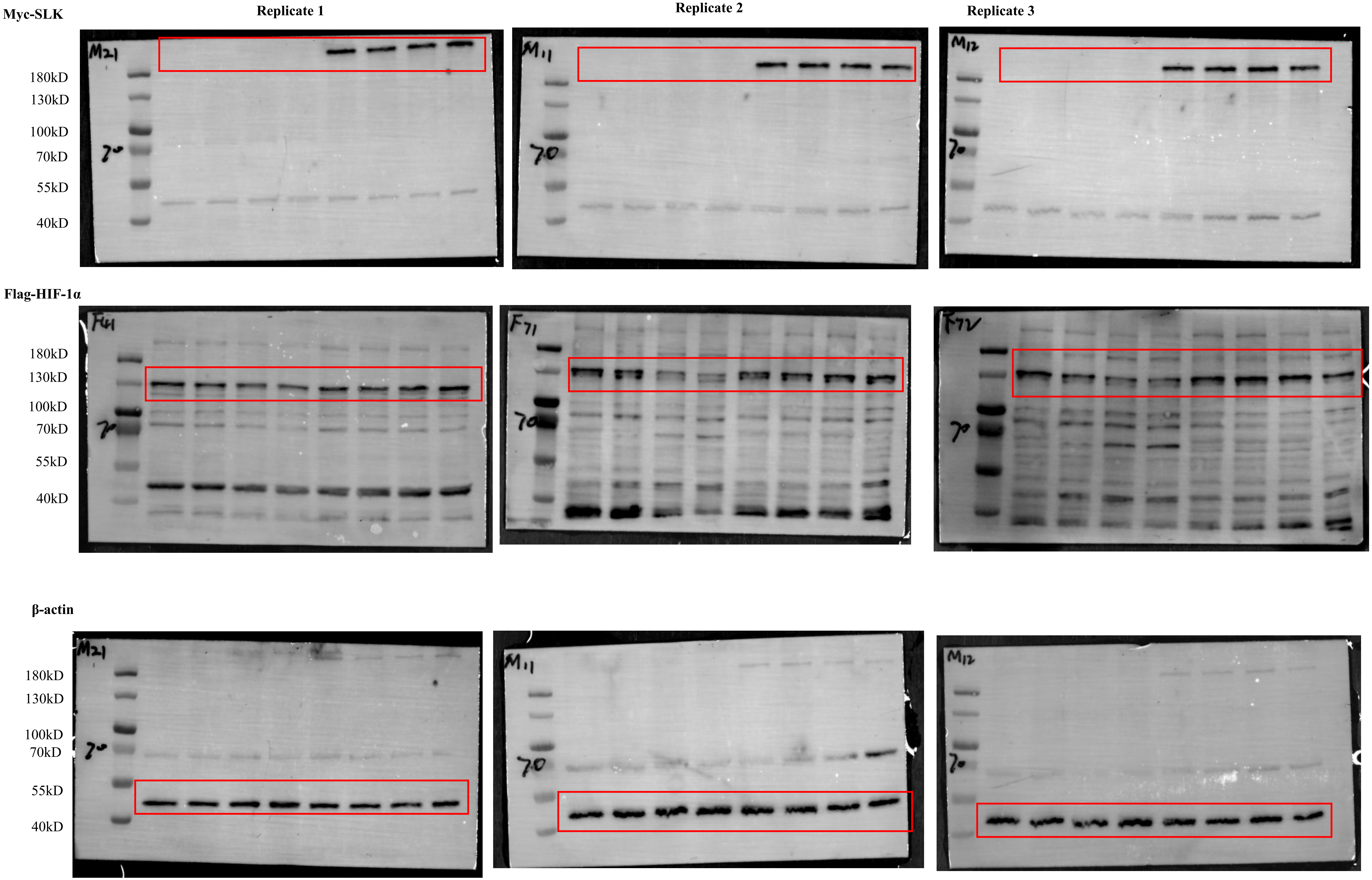
Myc-SLK
Replicate 1
Replicate 2
Replicate 3
180kD
130kD
100kD
70kD
55kD
40kD
Flag-HIF-1α
180kD
130kD
100kD
70kD
55kD
40kD
β-actin
180kD
130kD
100kD
70kD
55kD
40kD

Figure 7D

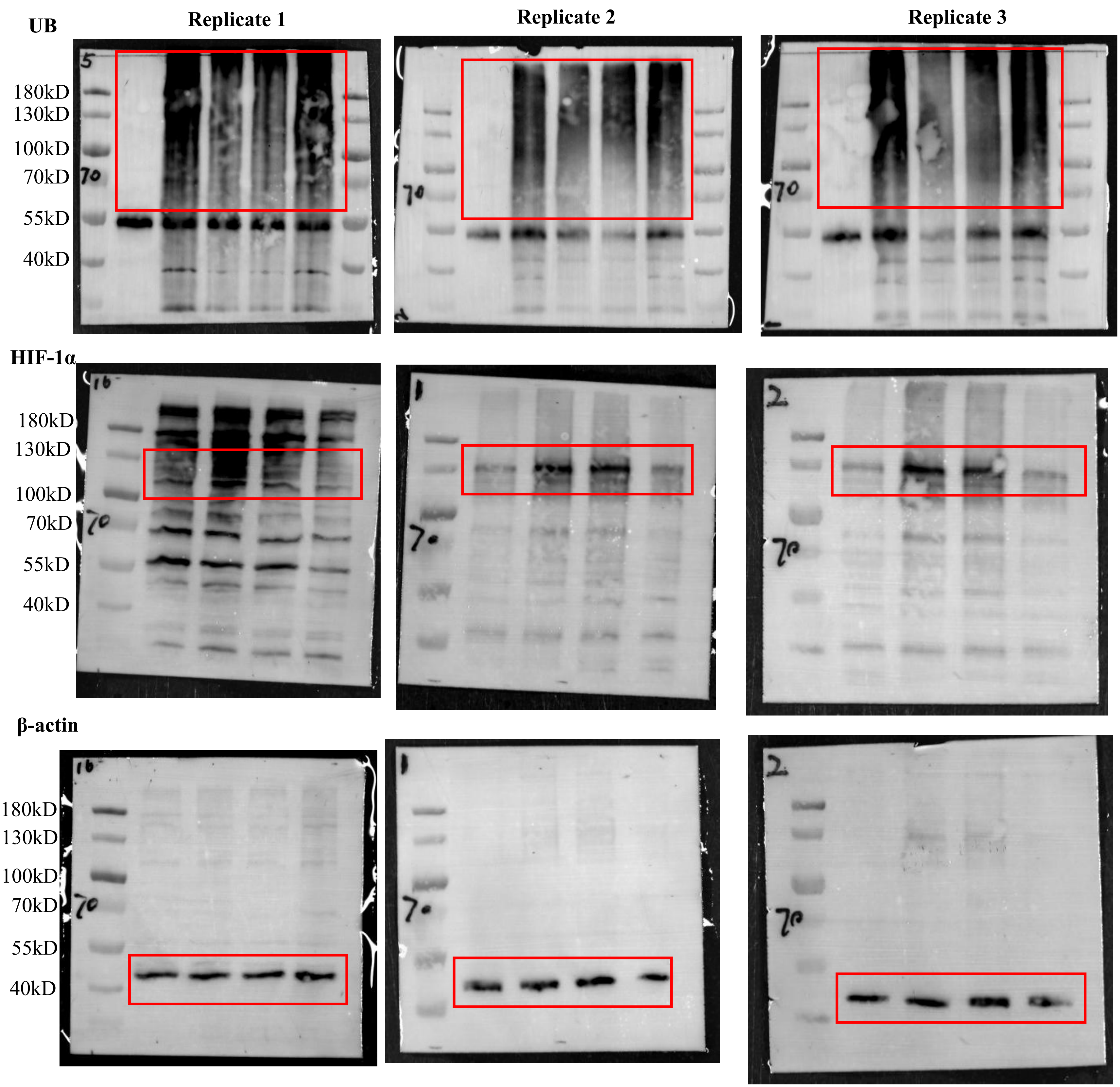
UB
Replicate 1
Replicate 2
Replicate 3
HIF-1α
β-actin
180kD
130kD
100kD
70kD
55kD
40kD

Figure 7F

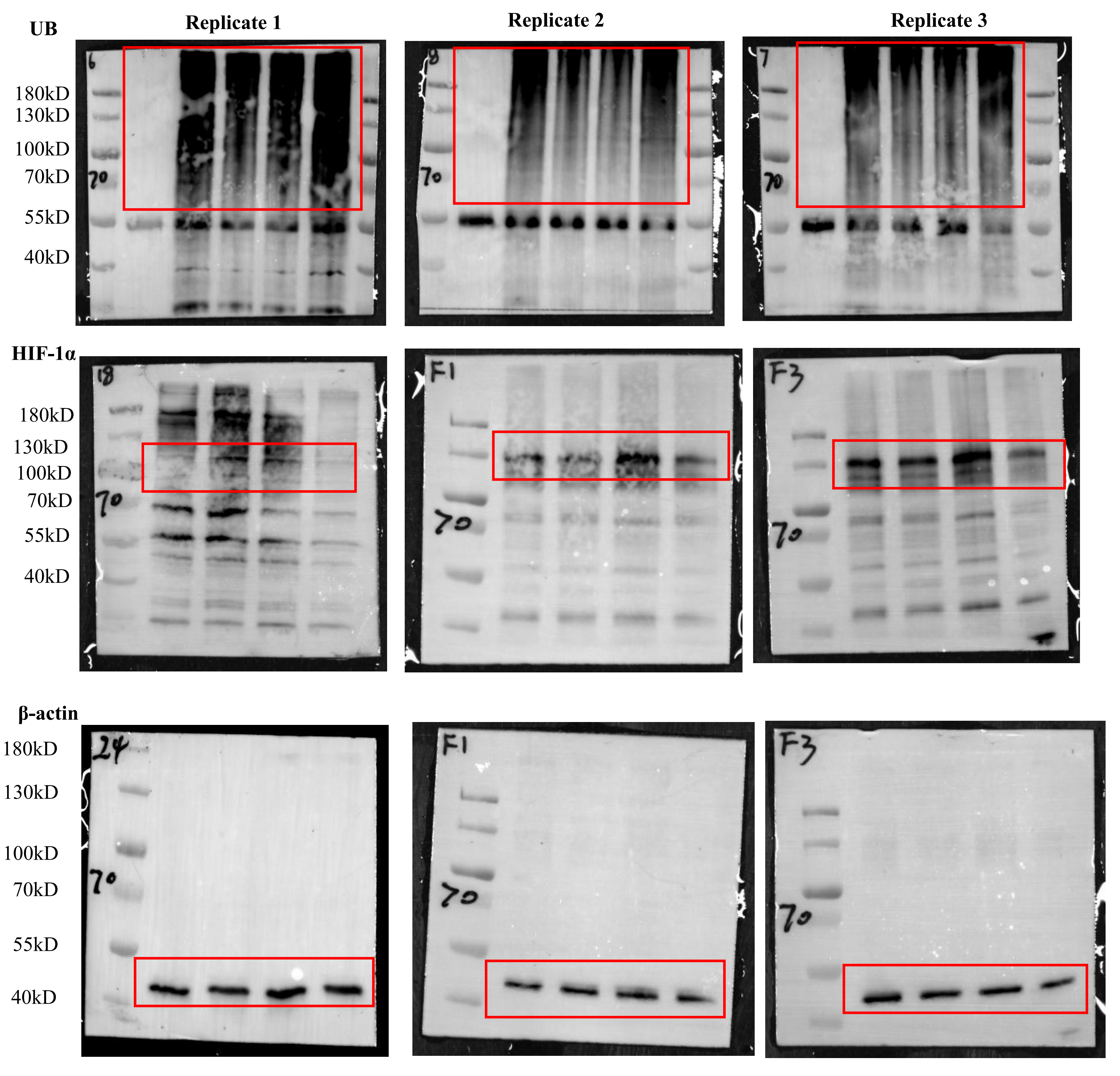
UB
Replicate 1
Replicate 2
Replicate 3
180kD
130kD
100kD
70kD
55kD
40kD
HIF-1α
180kD
130kD
100kD
70kD
55kD
40kD
β-actin
180kD
130kD
100kD
70kD
55kD
40kD

Figure 7H

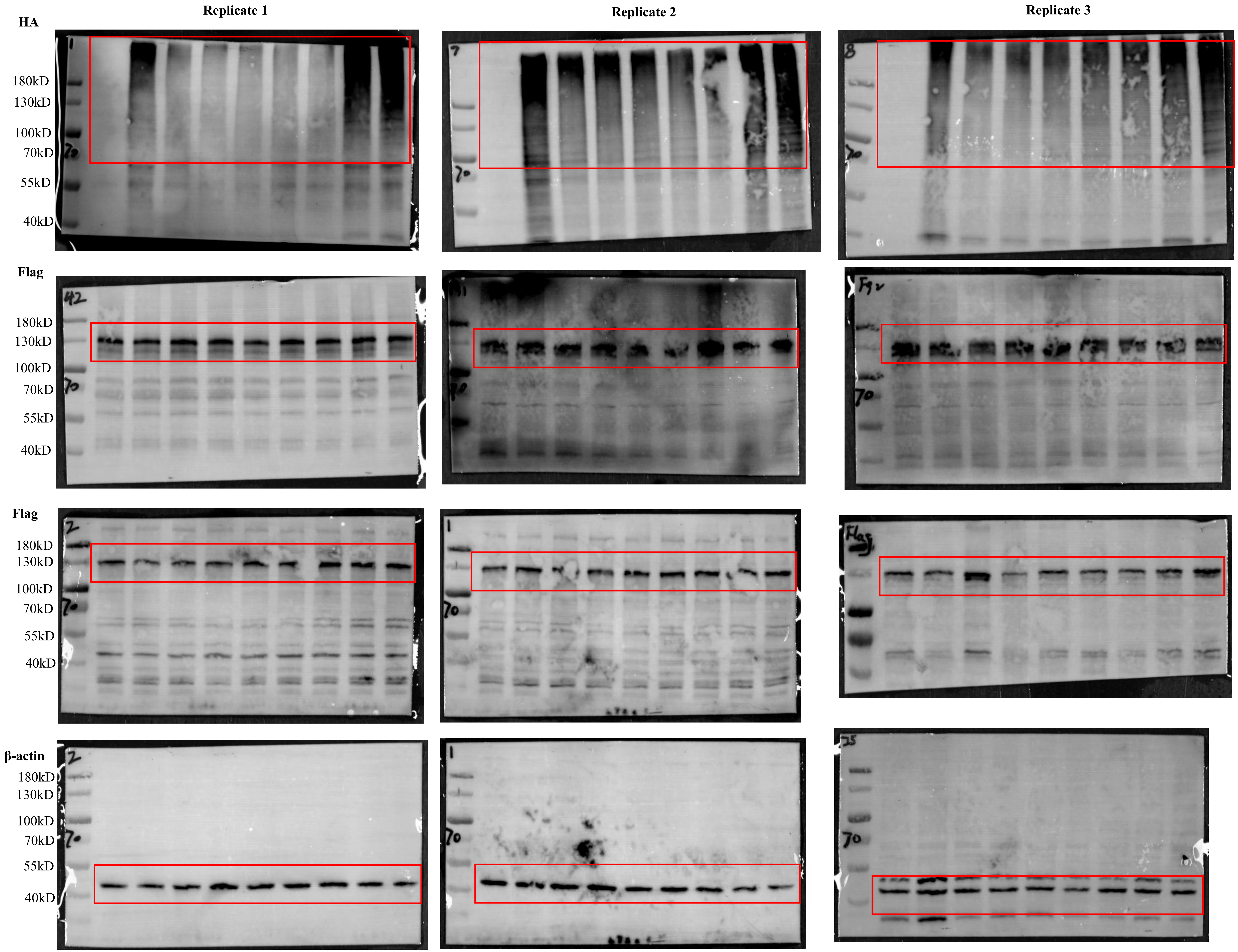
Replicate 1
Replicate 2
Replicate 3
HA
180kD
130kD
100kD
70kD
55kD
40kD
Flag
180kD
130kD
100kD
70kD
55kD
40kD
Flag
180kD
130kD
100kD
70kD
55kD
40kD
β-actin
180kD
130kD
100kD
70kD
55kD
40kD

Figure 7I (left)

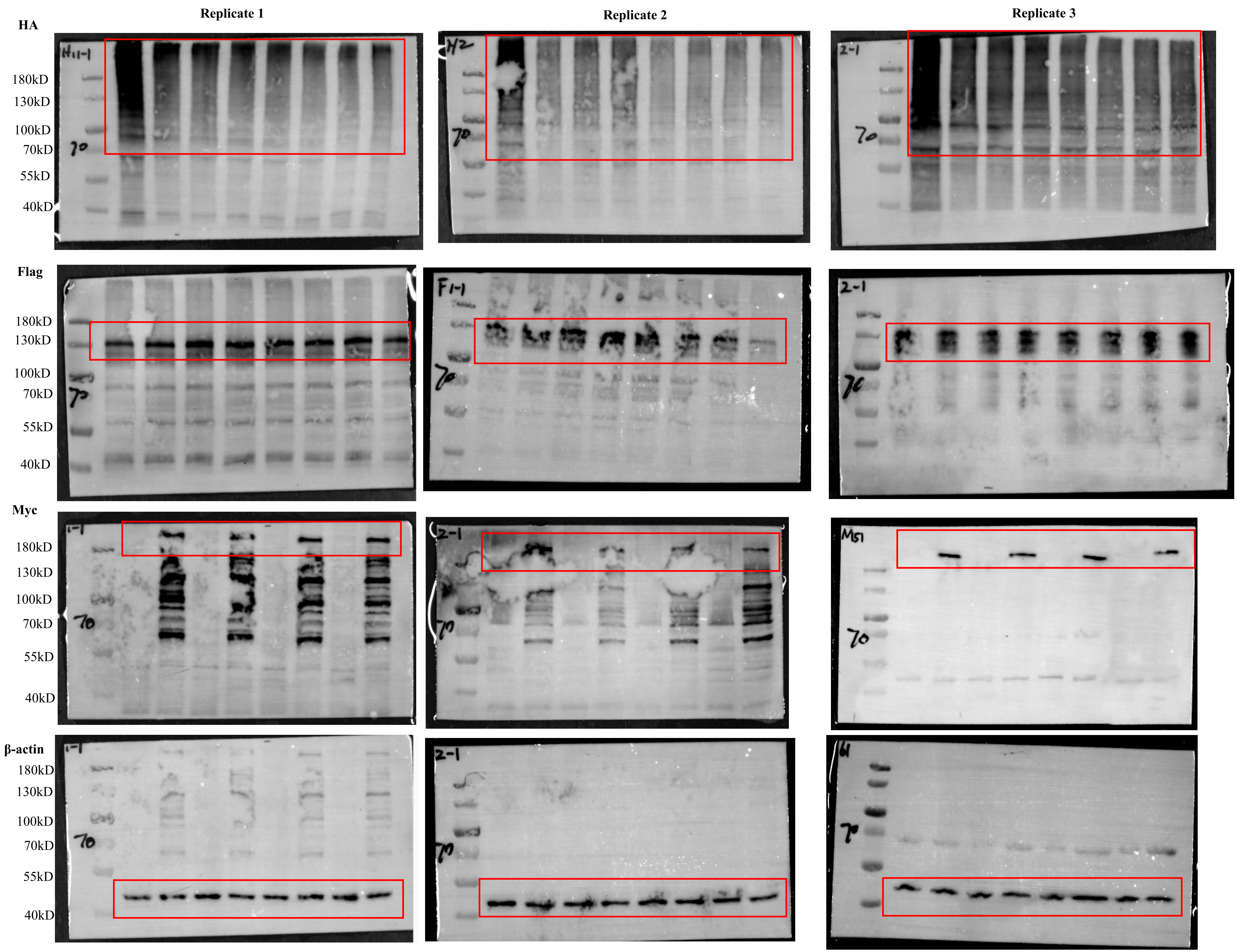

Figure 7I (right)

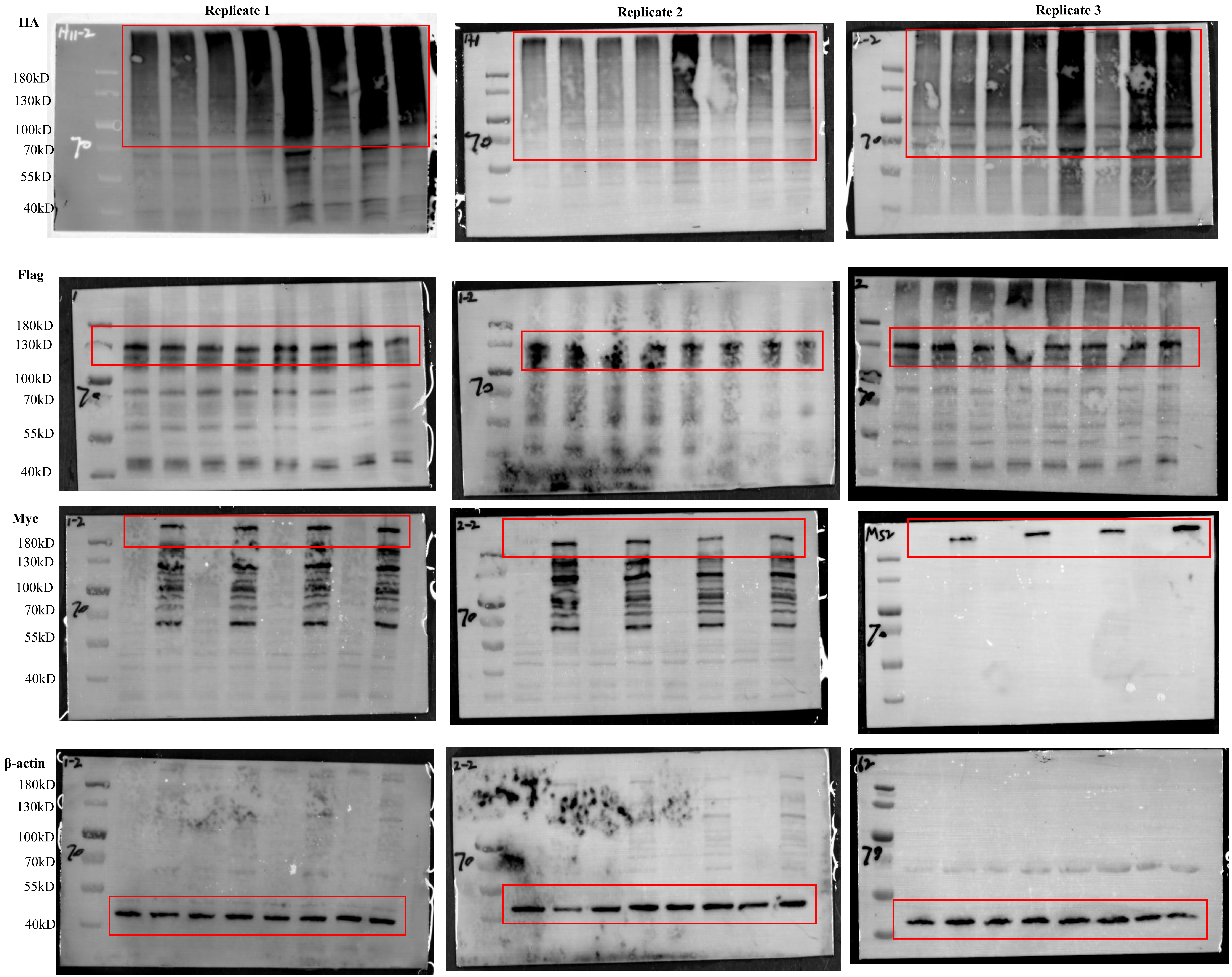

Figure 7J

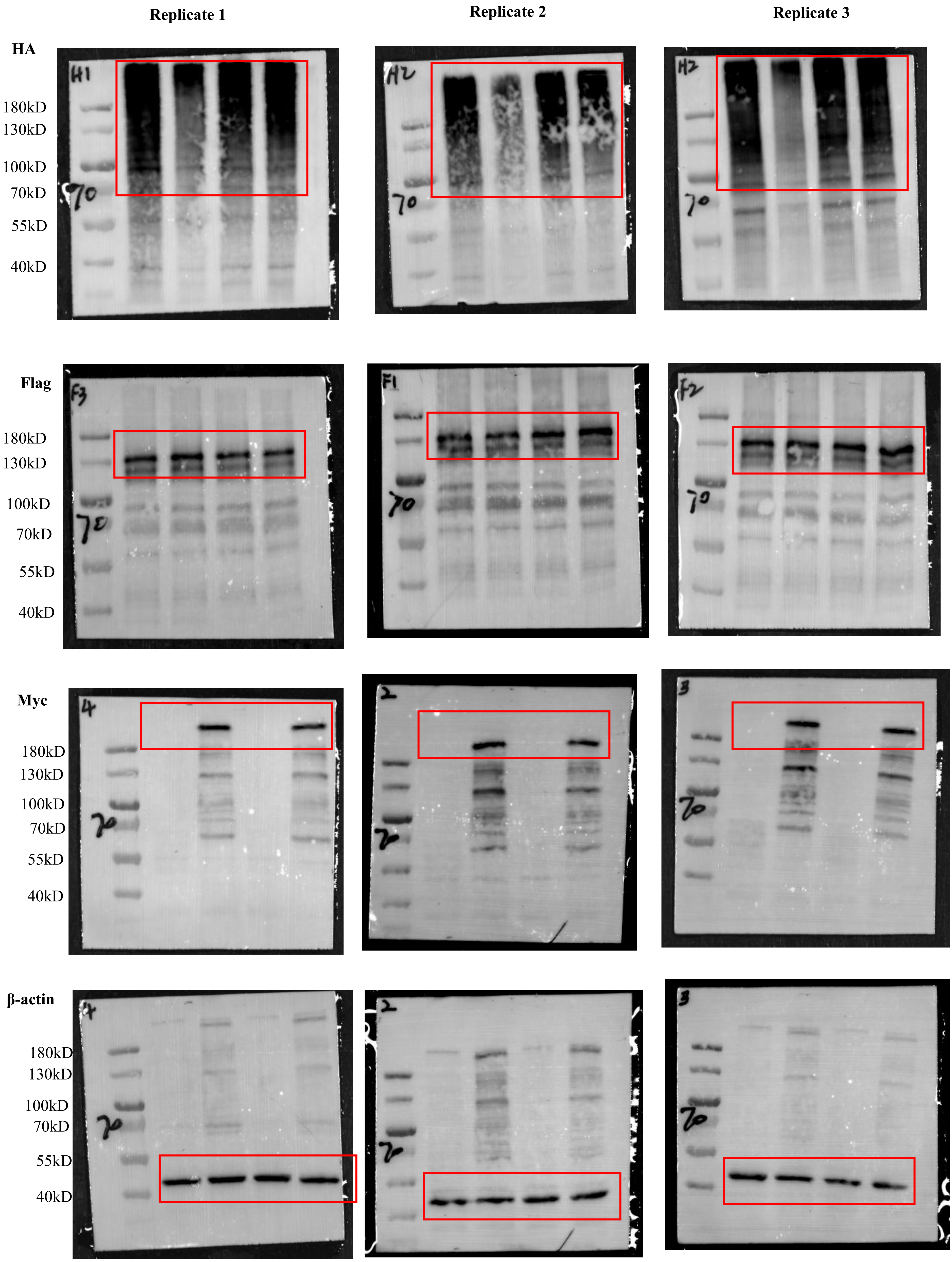
Replicate 1
Replicate 2
Replicate 3
HA
180kD
130kD
100kD
70kD
55kD
40kD
Flag
180kD
130kD
100kD
70kD
55kD
40kD
Myc
180kD
130kD
100kD
70kD
55kD
40kD
β-actin
180kD
130kD
100kD
70kD
55kD
40kD

Figure 8

Figure 8C

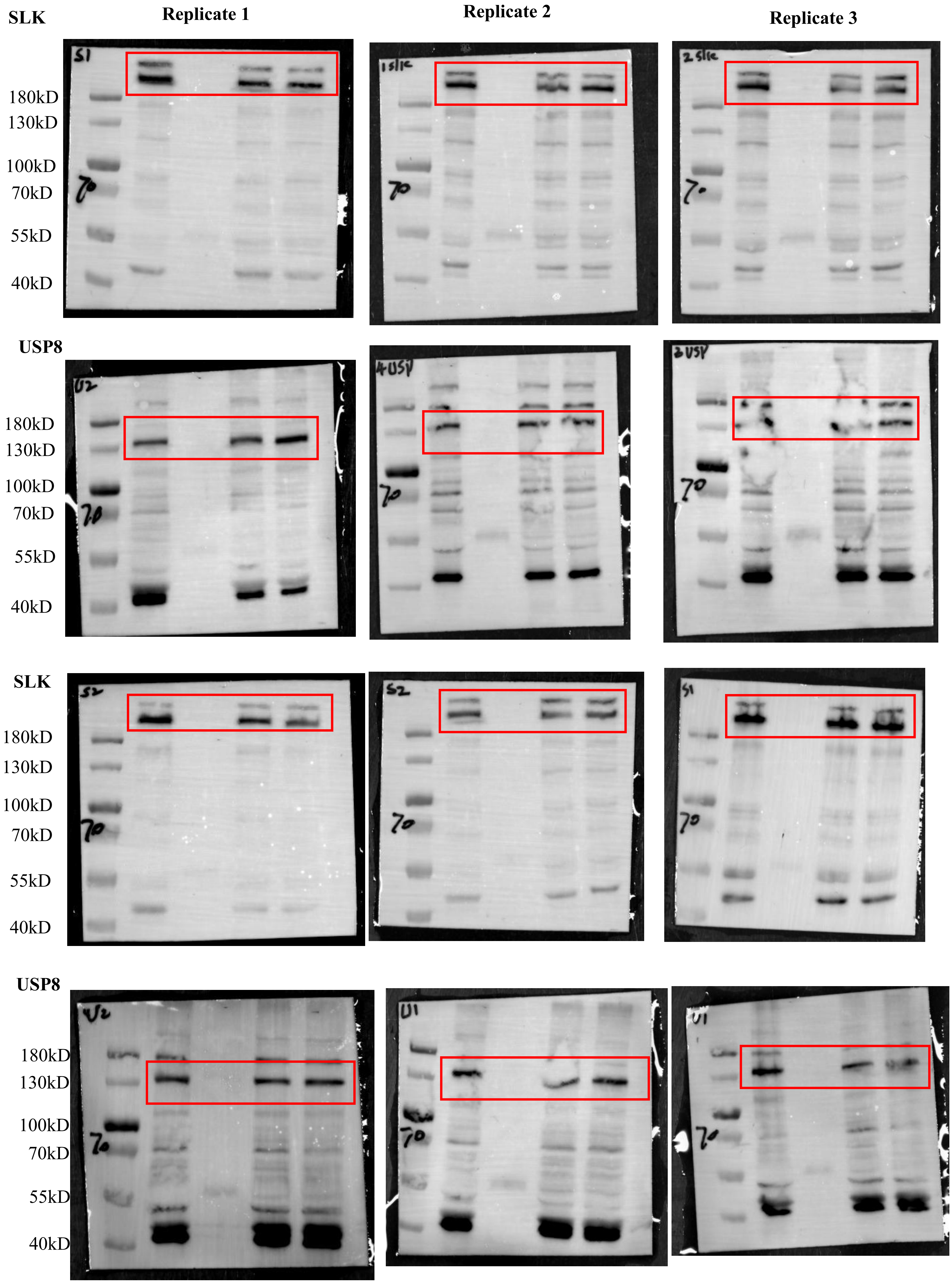

Figure 8D

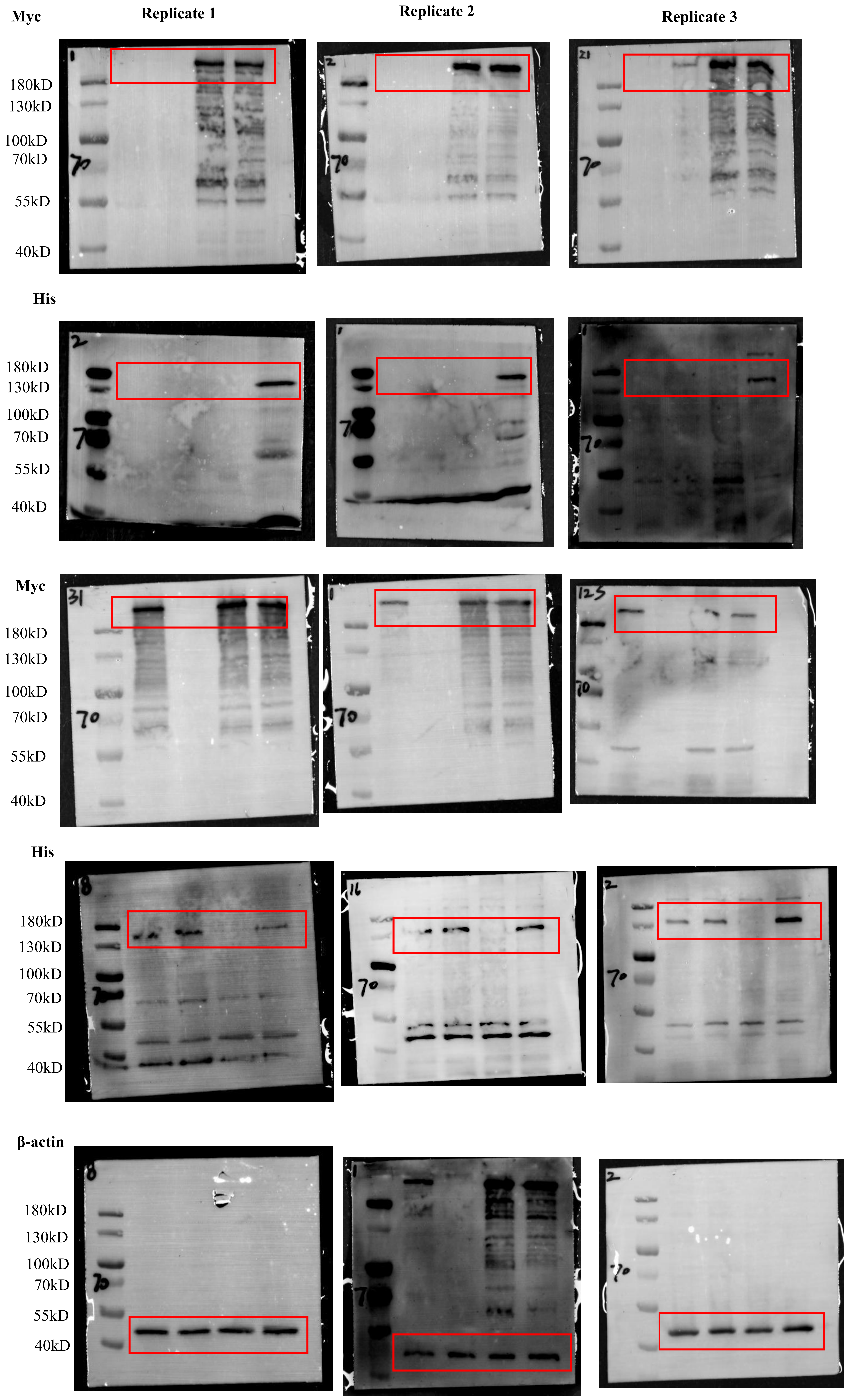
Myc
Replicate 1
Replicate 2
Replicate 3
180kD
130kD
100kD
70kD
55kD
40kD
His
180kD
130kD
100kD
70kD
55kD
40kD
Myc
180kD
130kD
100kD
70kD
55kD
40kD
His
180kD
130kD
100kD
70kD
55kD
40kD
β-actin
180kD
130kD
100kD
70kD
55kD
40kD

Figure 8E

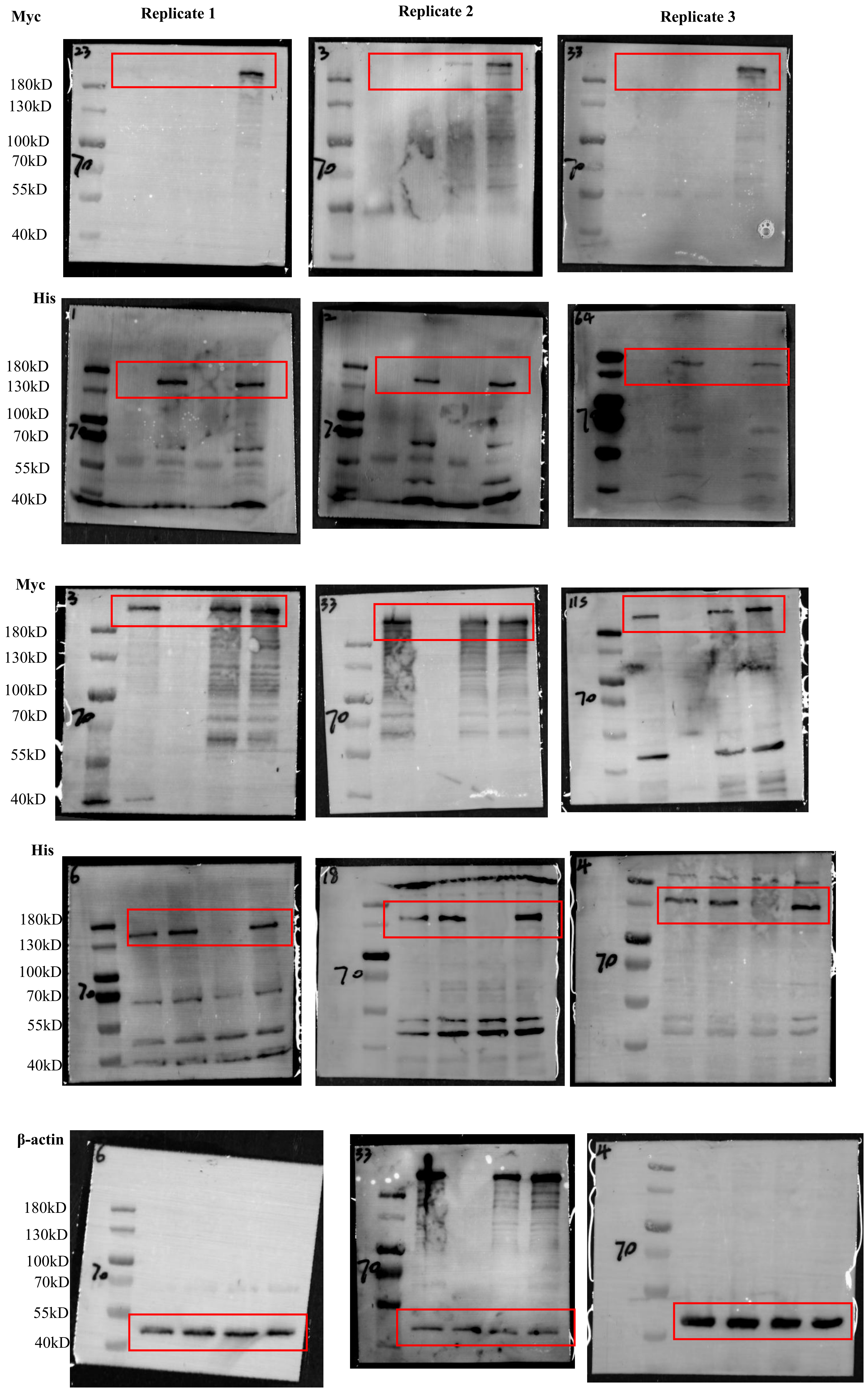

Myc
Replicate 1
Replicate 2
Replicate 3
180kD
130kD
100kD
70kD
55kD
40kD
His
180kD
130kD
100kD
70kD
55kD
40kD
Myc
180kD
130kD
100kD
70kD
55kD
40kD
His
180kD
130kD
100kD
70kD
55kD
40kD
β-actin
180kD
130kD
100kD
70kD
55kD
40kD

Figure 8G

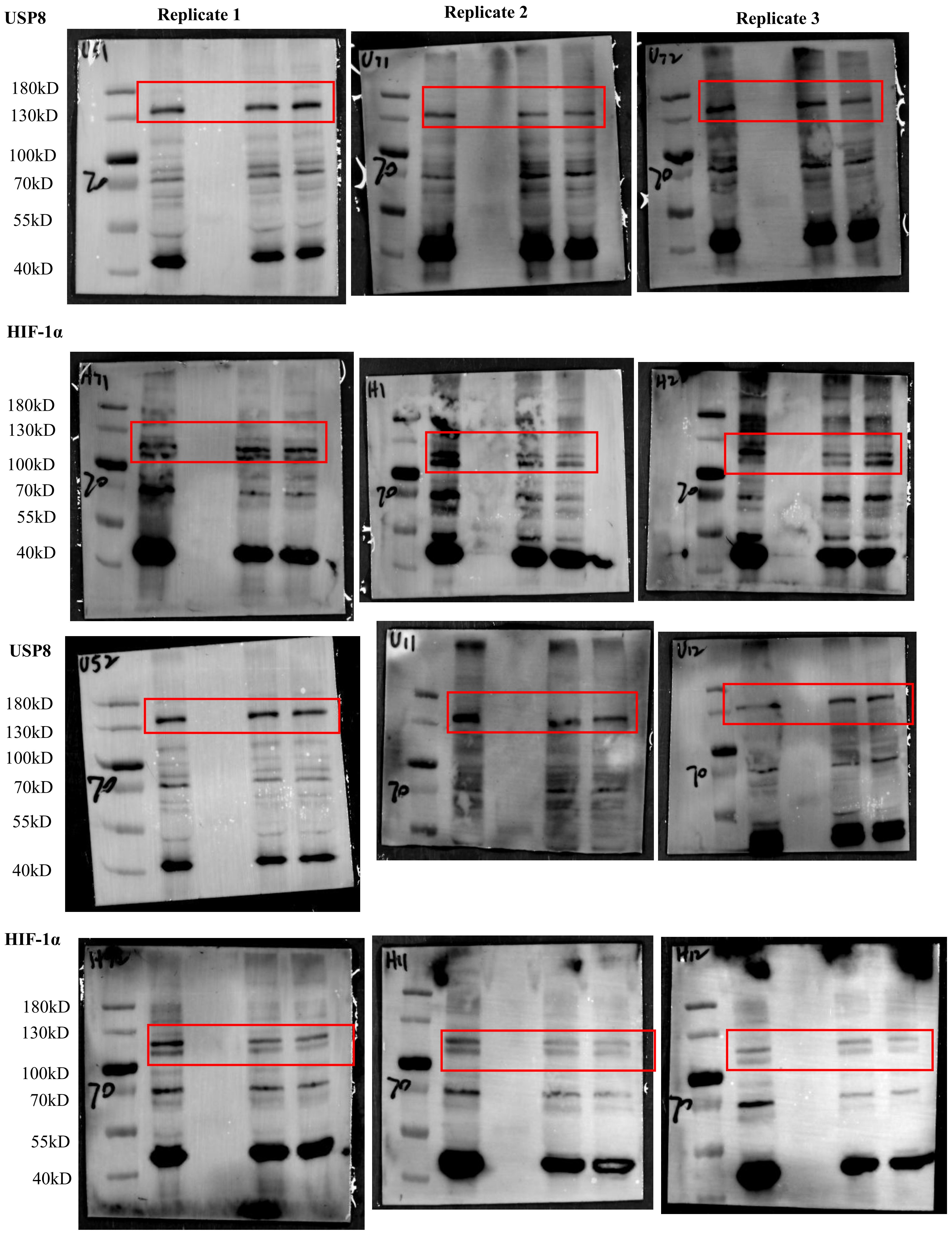
USP8
Replicate 1
Replicate 2
Replicate 3
180kD
130kD
100kD
70kD
55kD
40kD
HIF-1α
180kD
130kD
100kD
70kD
55kD
40kD
USP8
180kD
130kD
100kD
70kD
55kD
40kD
HIF-1α
180kD
130kD
100kD
70kD
55kD
40kD

Figure 8H

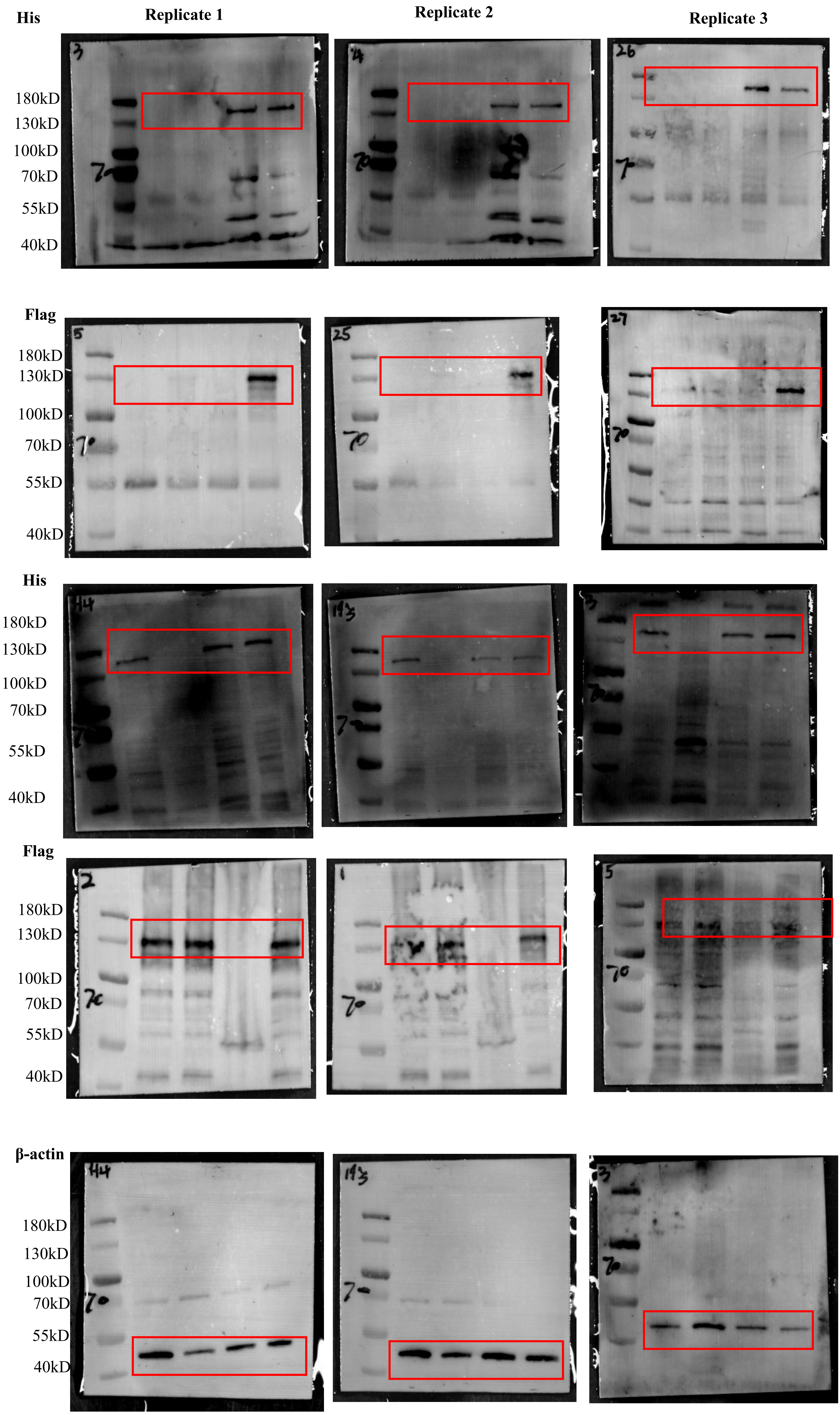
His
Replicate 1
Replicate 2
Replicate 3
180kD
130kD
100kD
70kD
55kD
40kD
Flag
180kD
130kD
100kD
70kD
55kD
40kD
His
180kD
130kD
100kD
70kD
55kD
40kD
Flag
180kD
130kD
100kD
70kD
55kD
40kD
β-actin
180kD
130kD
100kD
70kD
55kD
40kD

Figure 8I

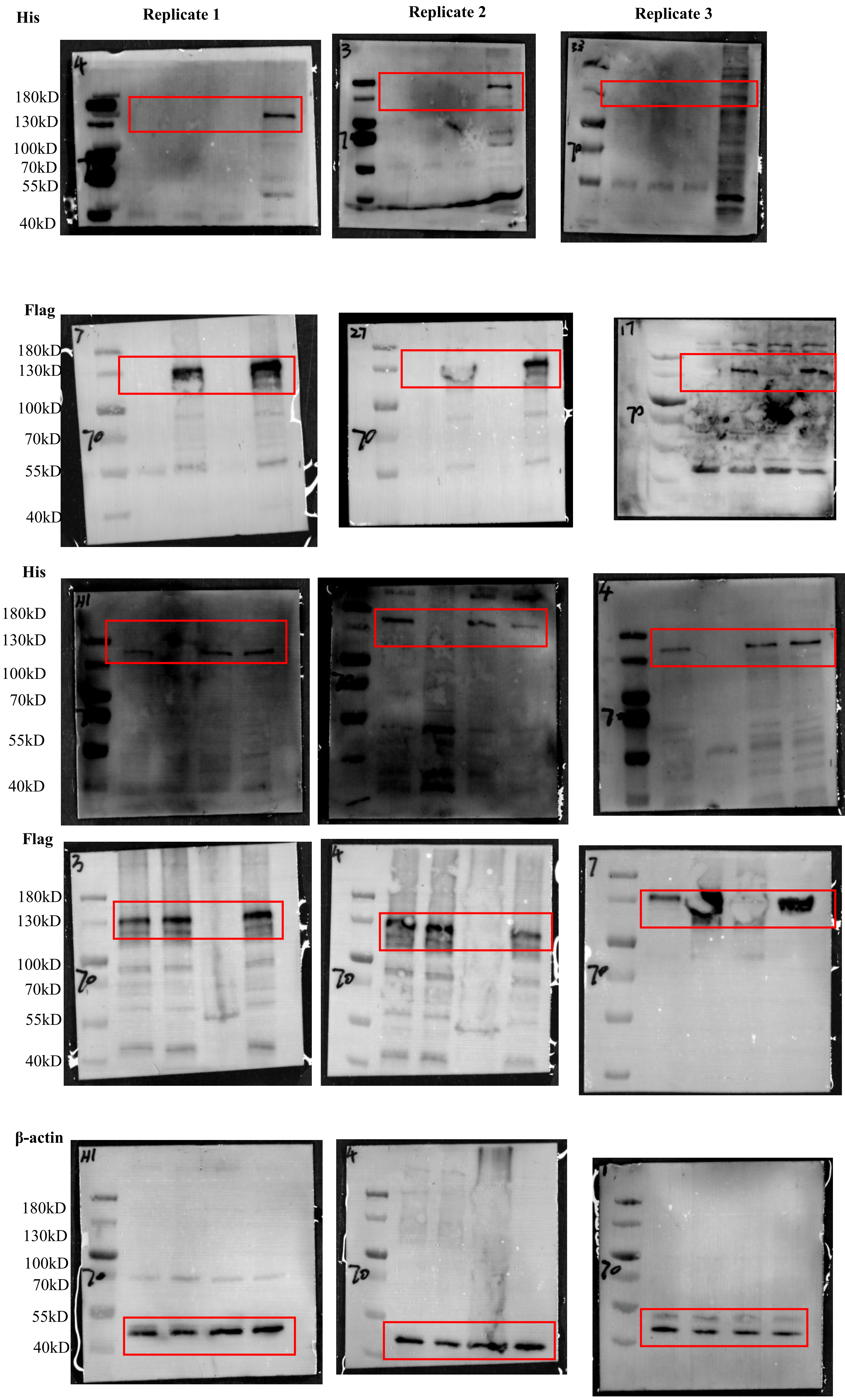

His
Replicate 1
Replicate 2
Replicate 3
180kD
130kD
100kD
70kD
55kD
40kD
Flag
180kD
130kD
100kD
70kD
55kD
40kD
His
180kD
130kD
100kD
70kD
55kD
40kD
Flag
180kD
130kD
100kD
70kD
55kD
40kD
β-actin
180kD
130kD
100kD
70kD
55kD
40kD

Figure 9

Figure 9A (left)

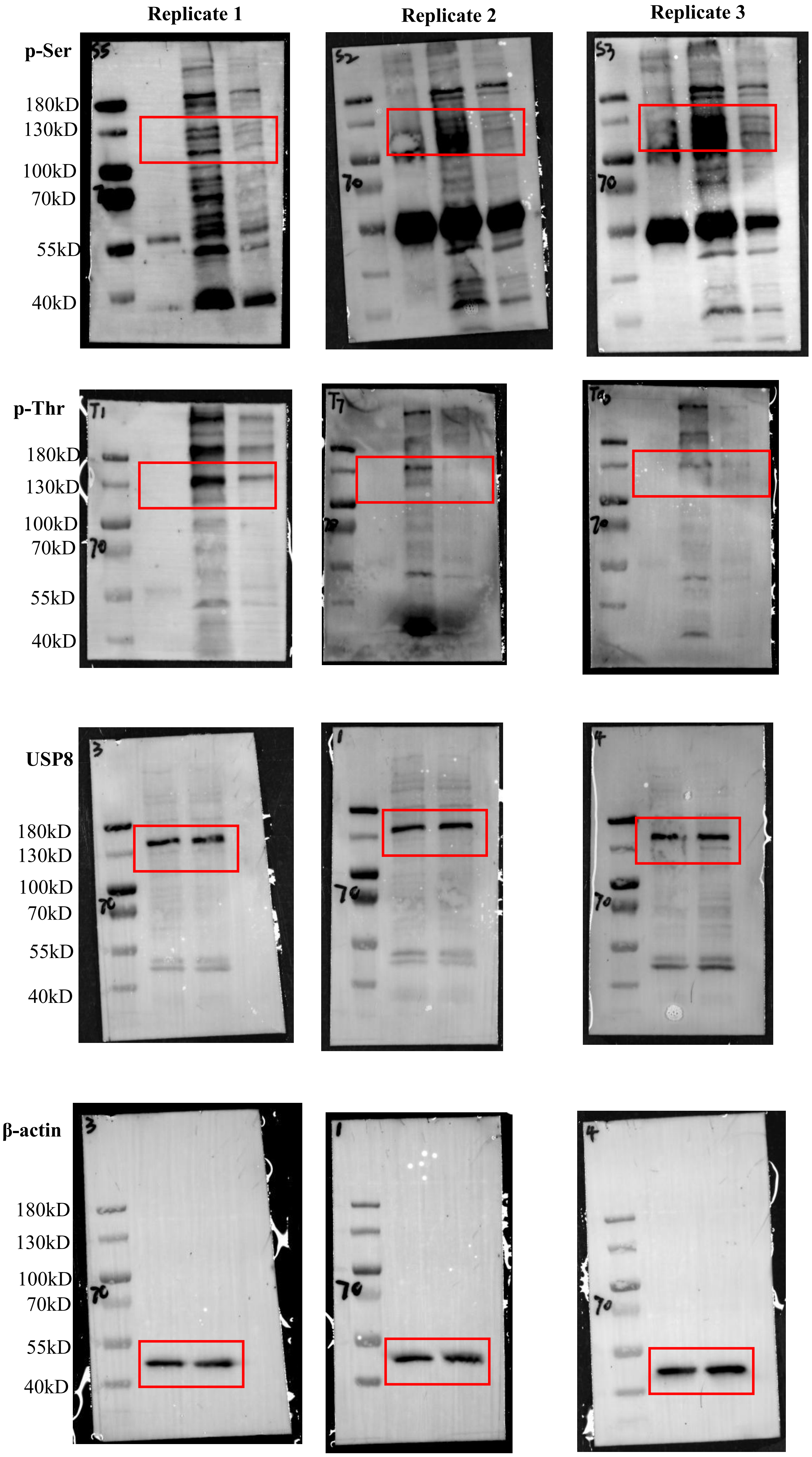

Figure 9A (right)

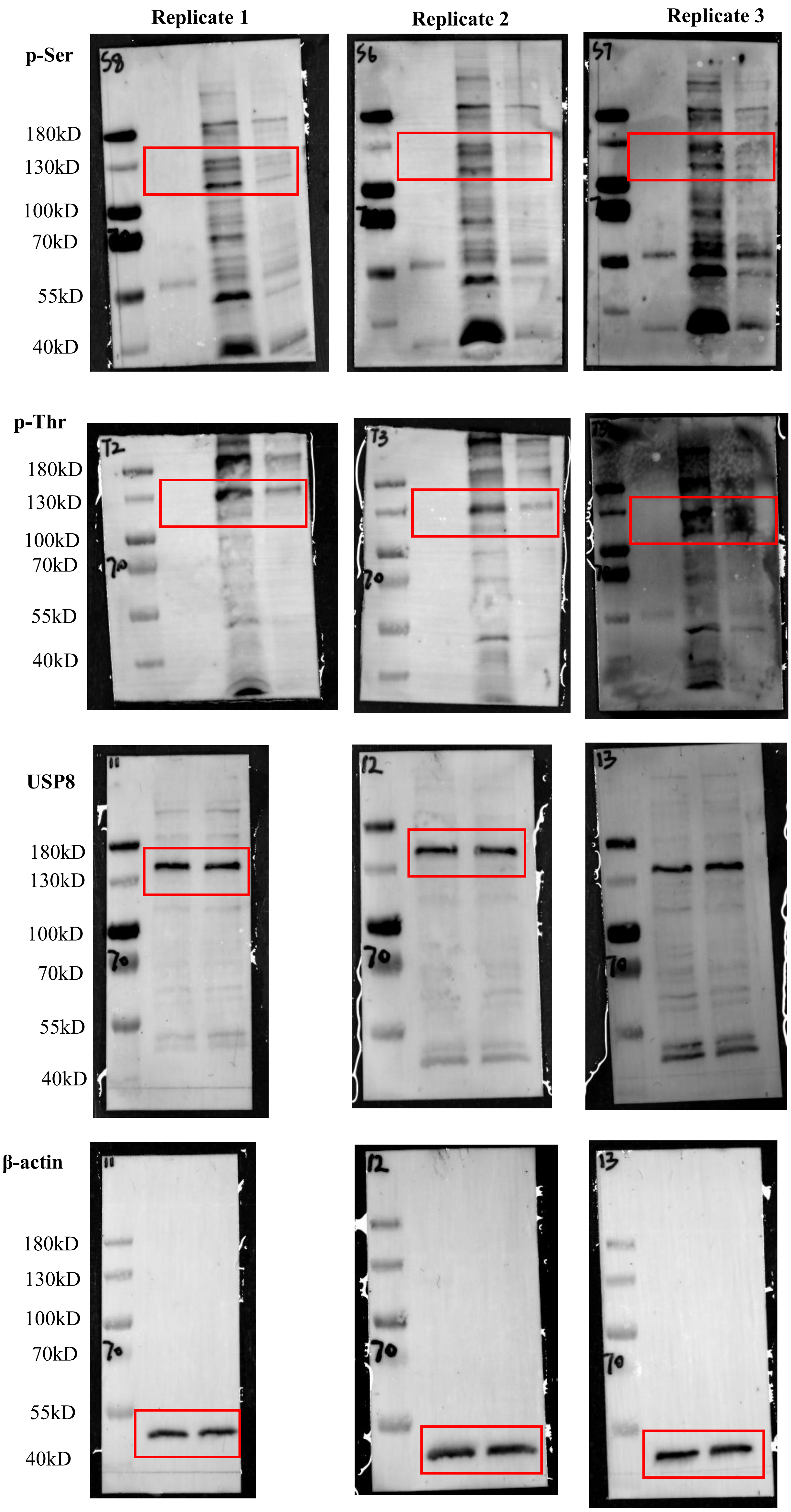

Figure 9B

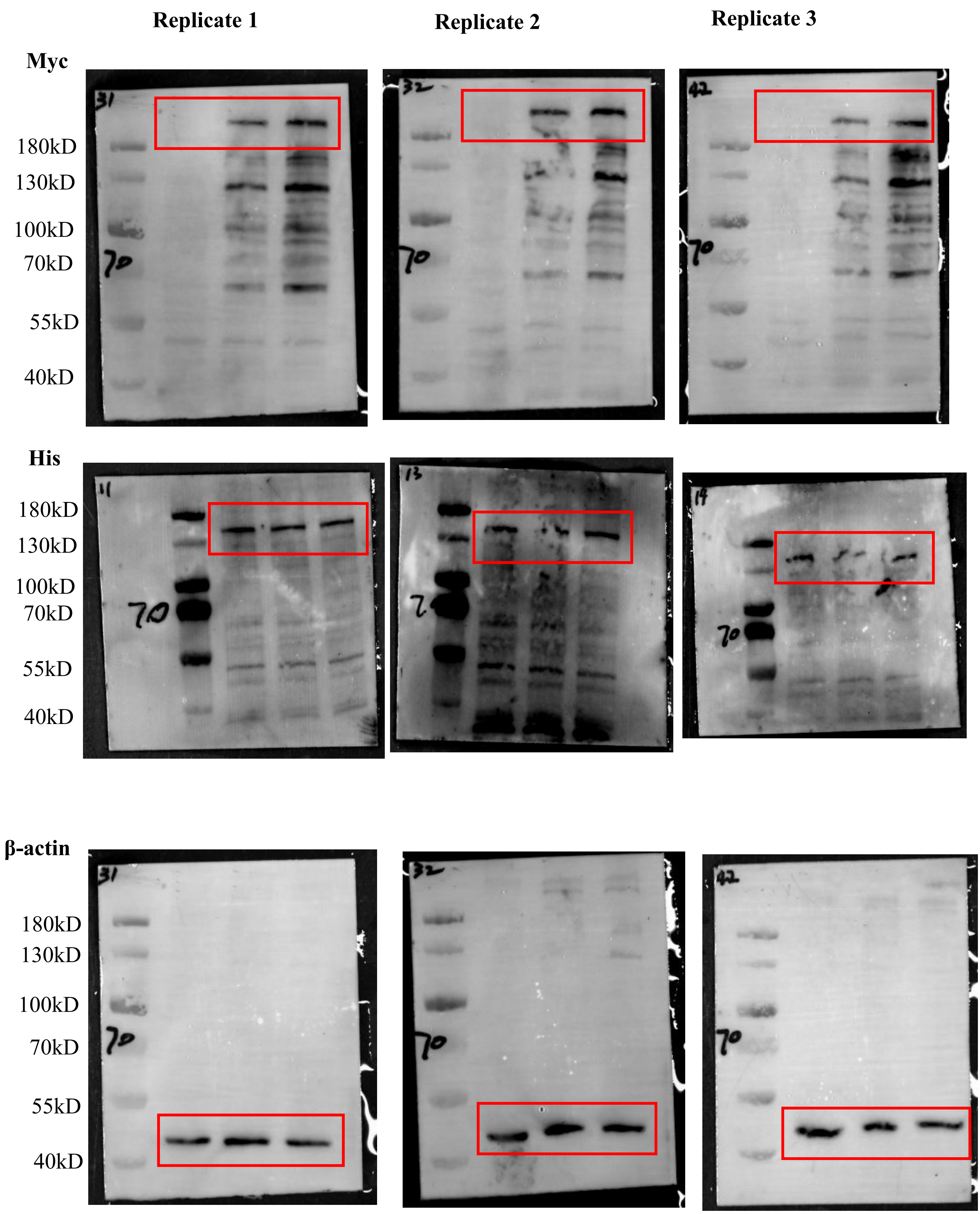
Replicate 1
Replicate 2
Replicate 3
Myc
180kD
130kD
100kD
70kD
55kD
40kD
His
180kD
130kD
100kD
70kD
55kD
40kD
β-actin
180kD
130kD
100kD
70kD
55kD
40kD

Figure 9C

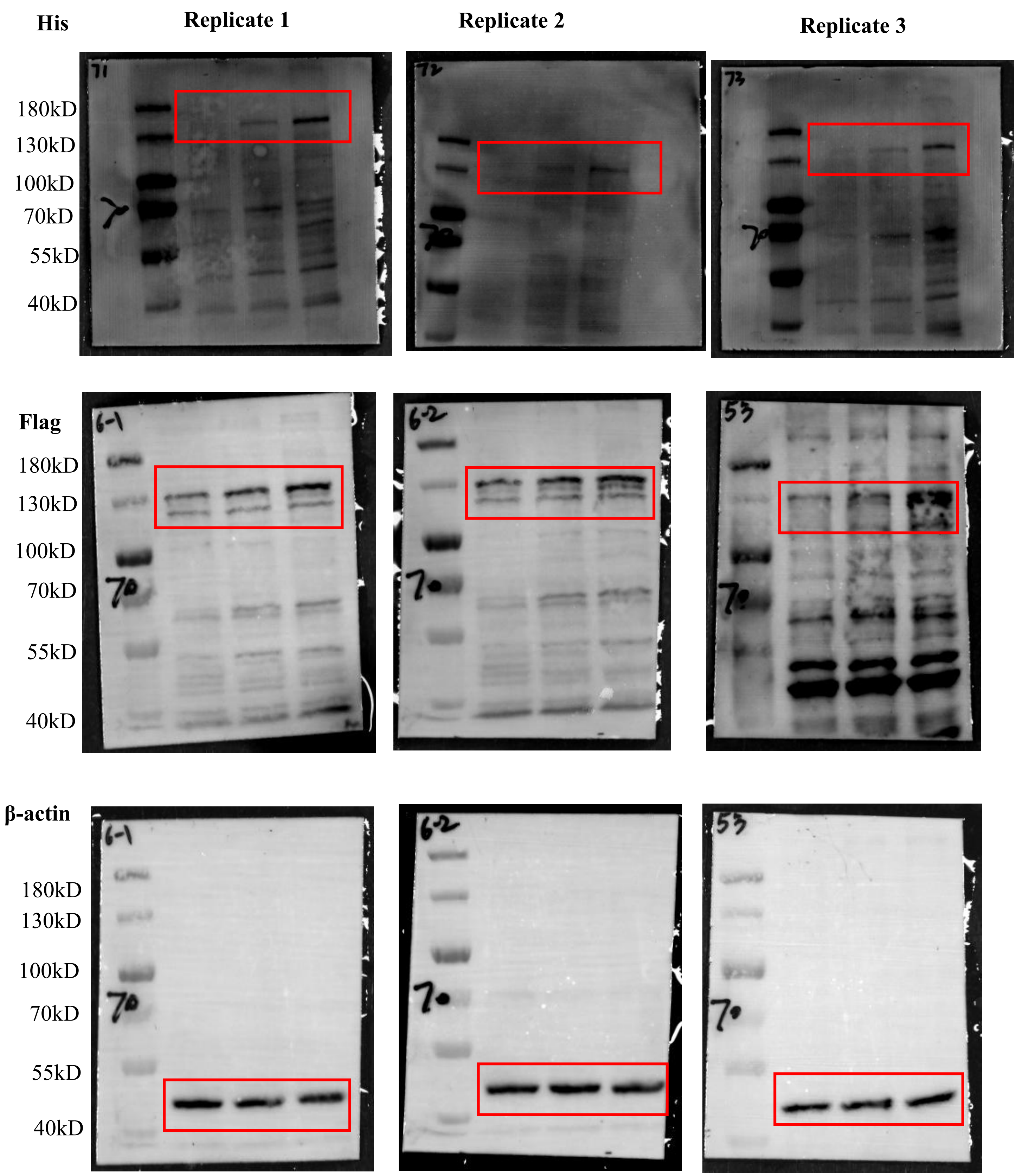

His
Replicate 1
Replicate 2
Replicate 3
180kD
130kD
100kD
70kD
55kD
40kD
Flag
180kD
130kD
100kD
70kD
55kD
40kD
β-actin
180kD
130kD
100kD
70kD
55kD
40kD

Figure 9D

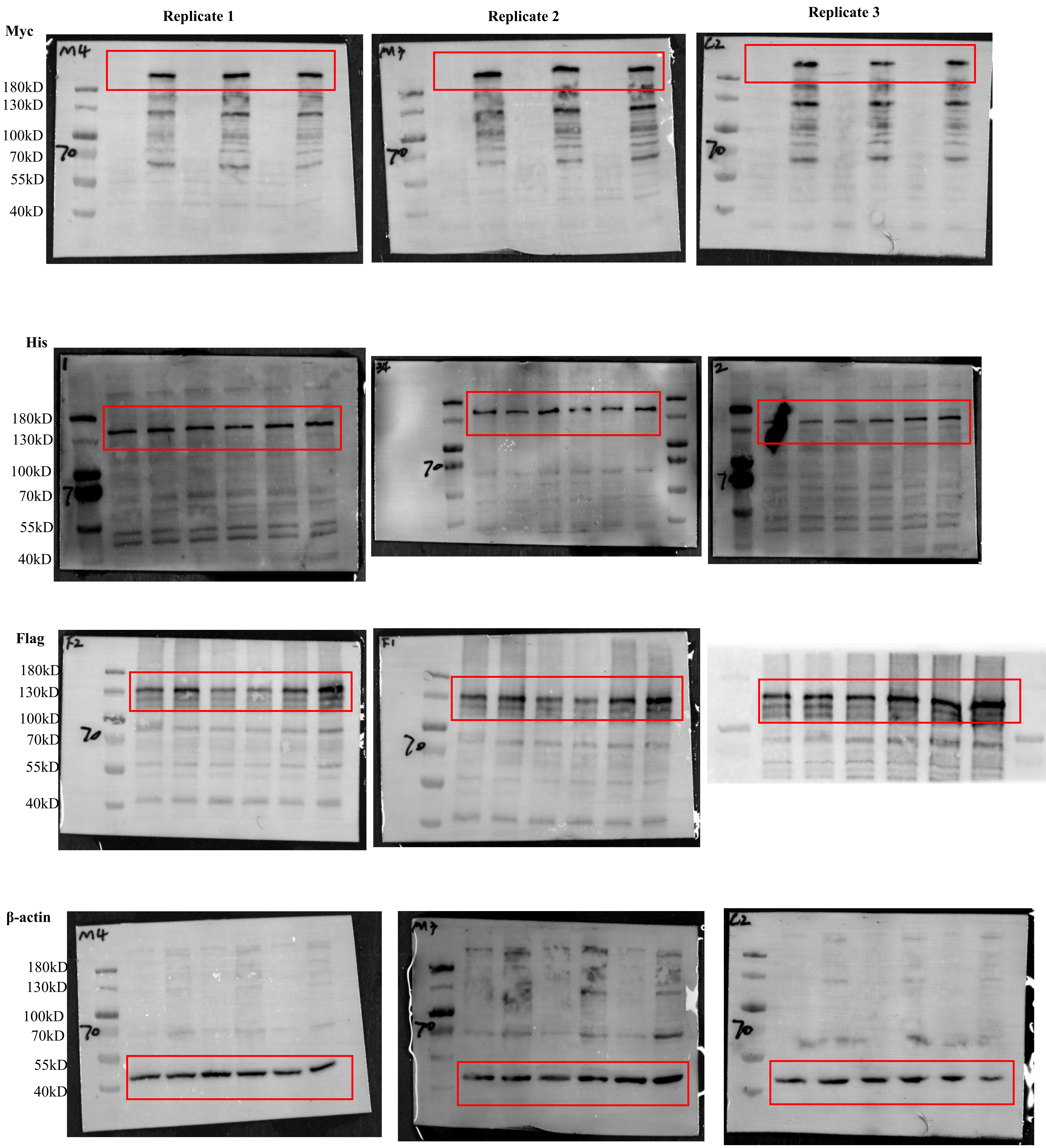
Replicate 1
Replicate 2
Replicate 3
Myc
180kD
130kD
100kD
70kD
55kD
40kD
His
180kD
130kD
100kD
70kD
55kD
40kD
Flag
180kD
130kD
100kD
70kD
55kD
40kD
β-actin
180kD
130kD
100kD
70kD
55kD
40kD

Figure 9E

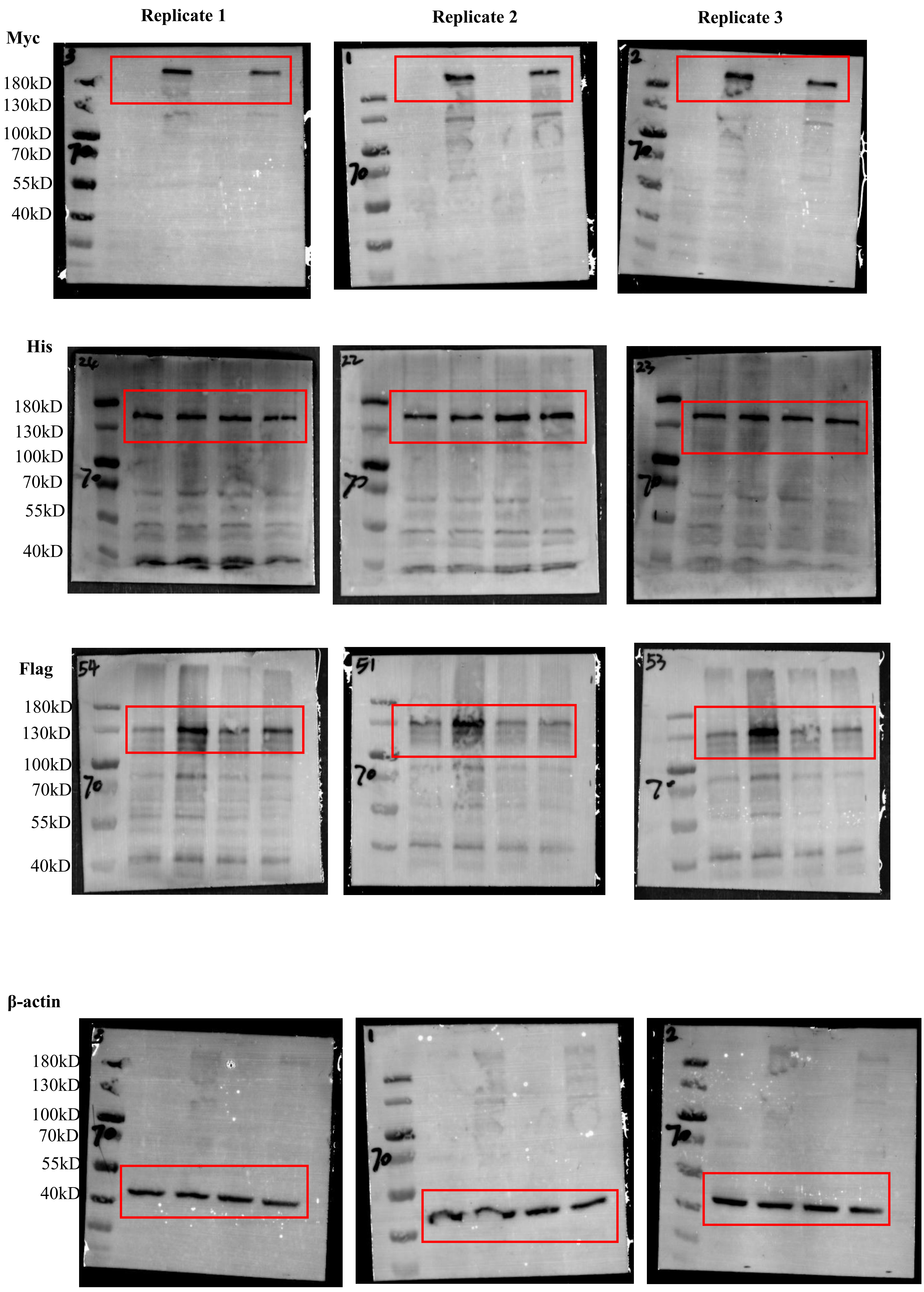
Replicate 1
Replicate 2
Replicate 3
Myc
180kD
130kD
100kD
70kD
55kD
40kD
His
180kD
130kD
100kD
70kD
55kD
40kD
Flag
180kD
130kD
100kD
70kD
55kD
40kD
β-actin
180kD
130kD
100kD
70kD
55kD
40kD

Figure 9F (left)

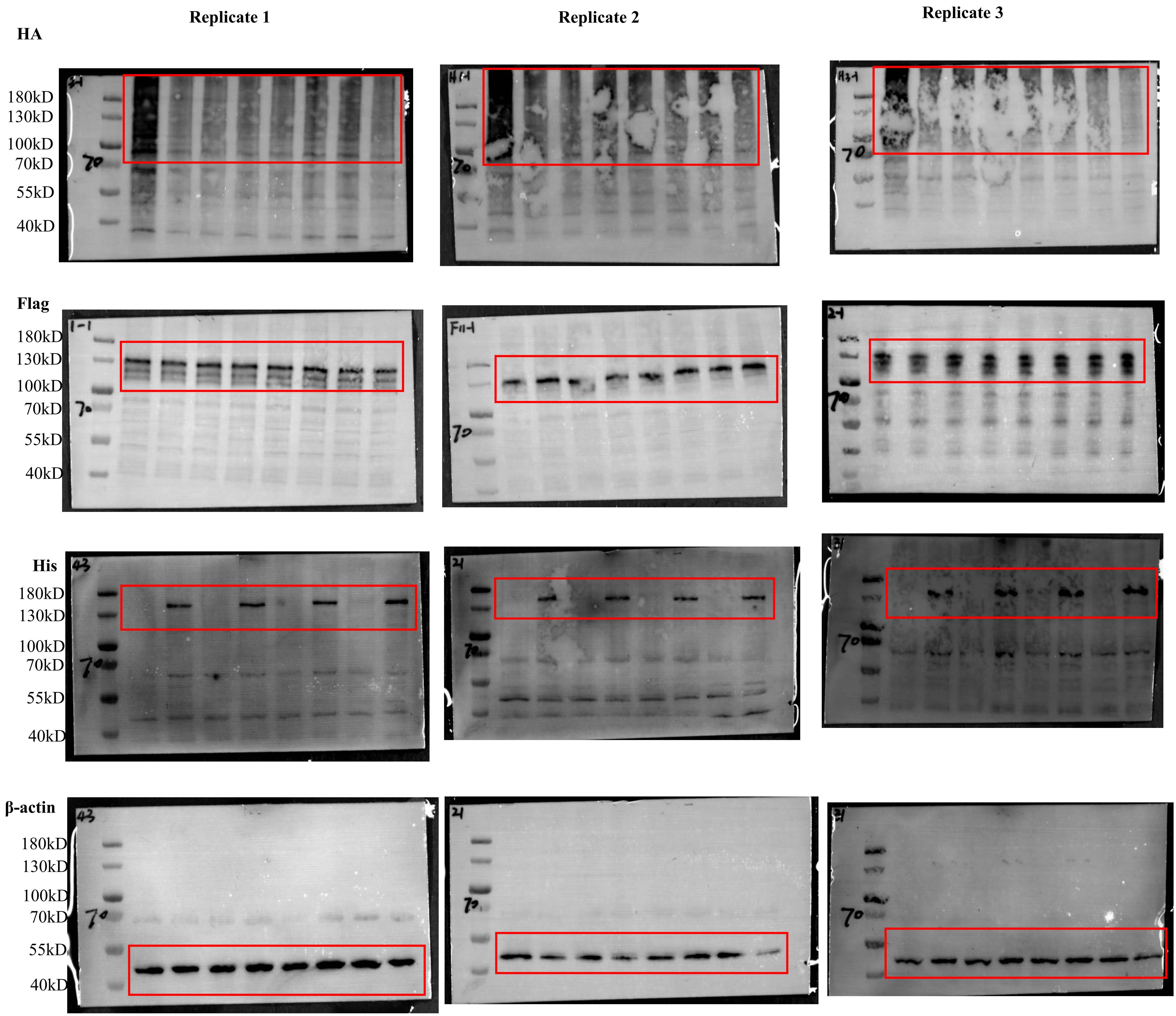

Figure 9F (right)

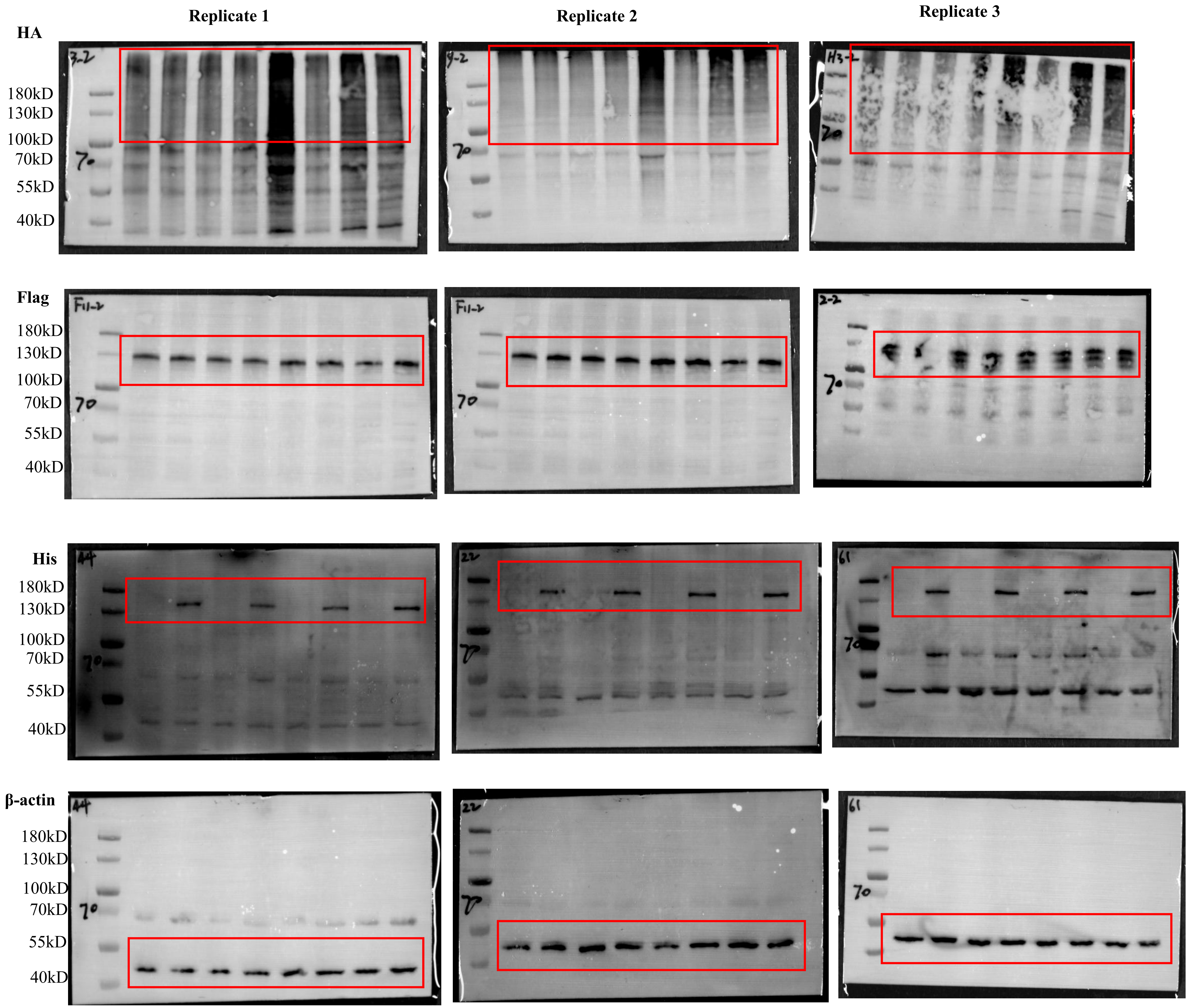

Figure 9G

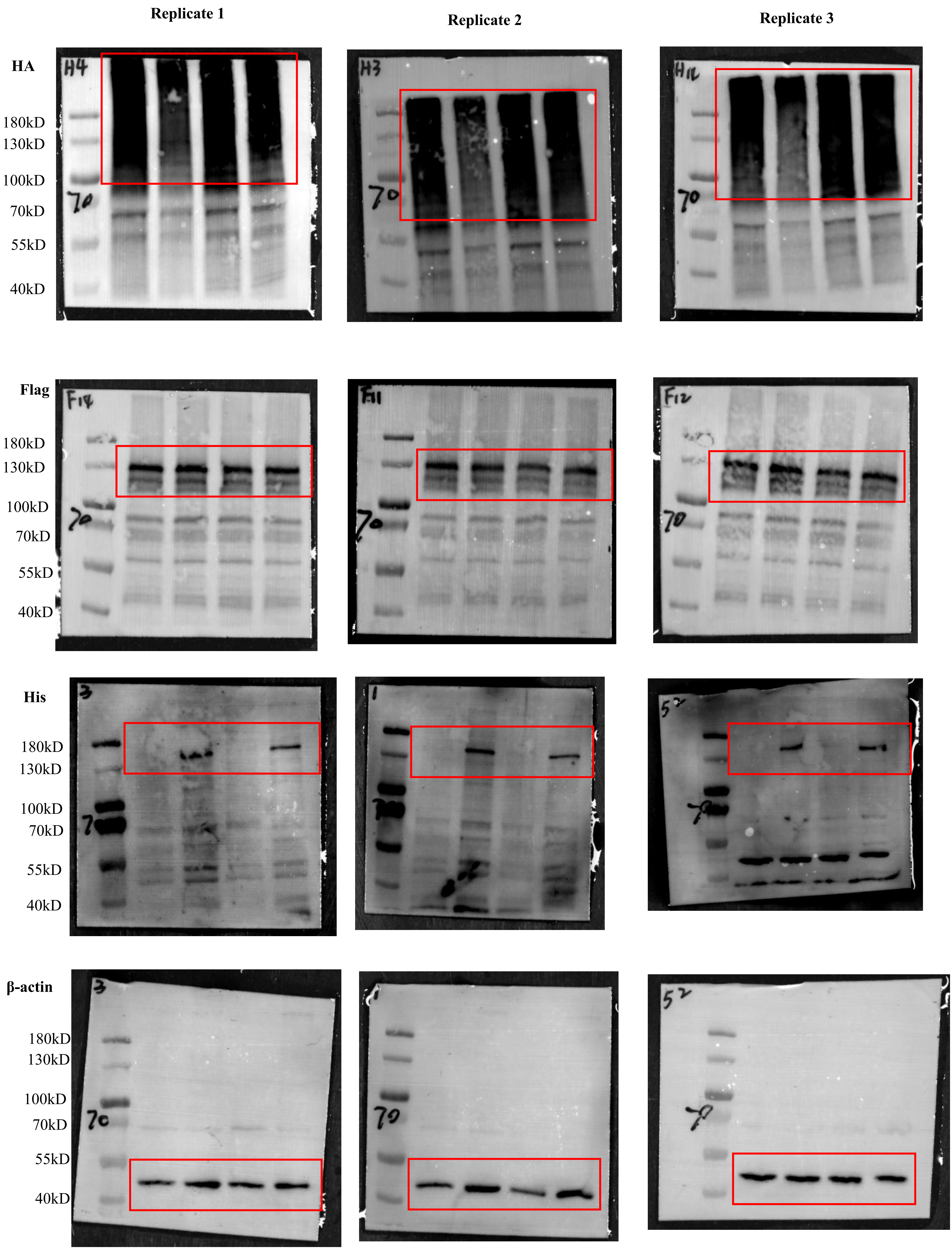
Replicate 1
Replicate 2
Replicate 3
HA
180kD
130kD
100kD
70kD
55kD
40kD
Flag
180kD
130kD
100kD
70kD
55kD
40kD
His
180kD
130kD
100kD
70kD
55kD
40kD
β-actin
180kD
130kD
100kD
70kD
55kD
40kD

Figure 9H

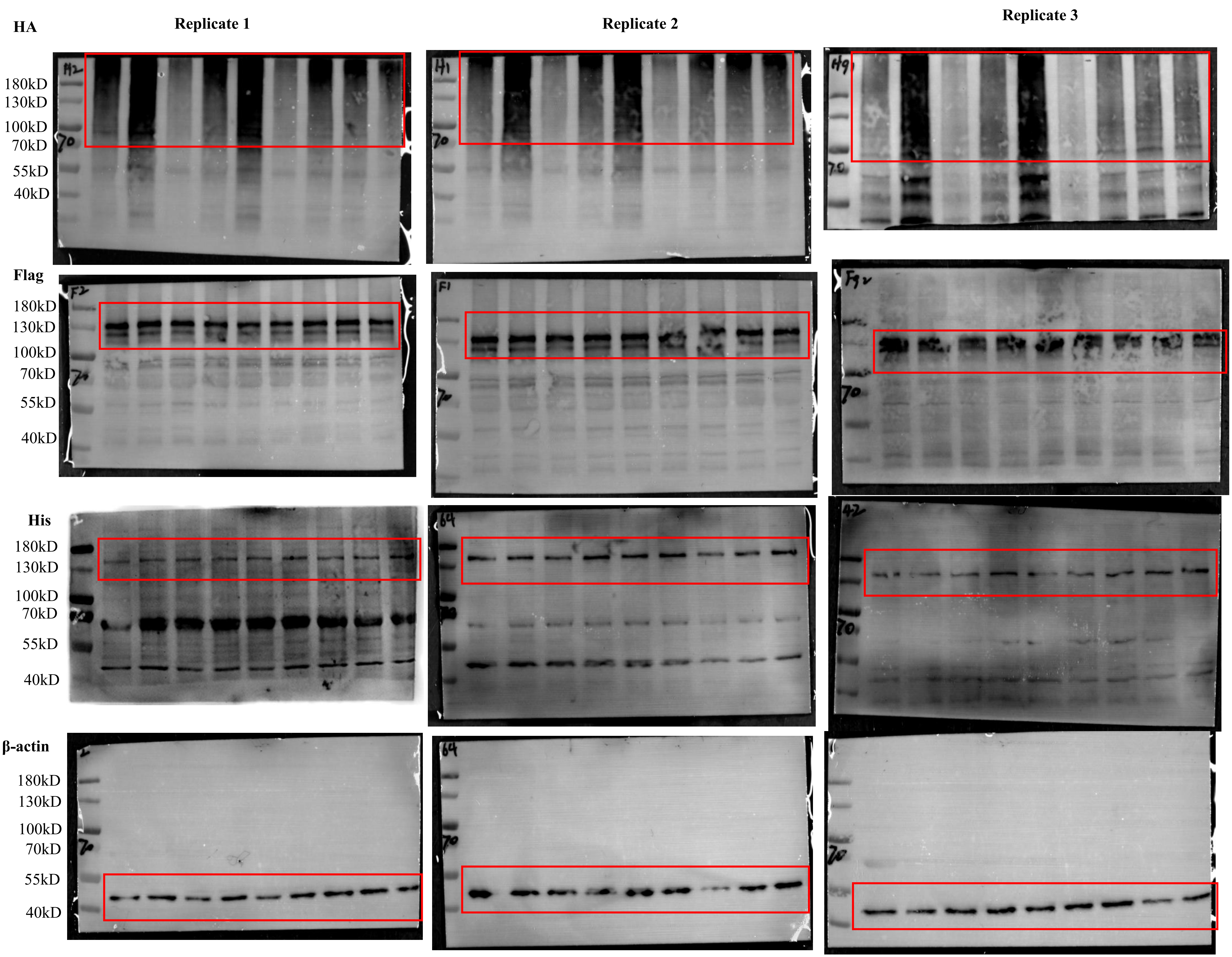

HA
Replicate 1
Replicate 2
Replicate 3
180kD
130kD
100kD
70kD
55kD
40kD
Flag
180kD
130kD
100kD
70kD
55kD
40kD
His
180kD
130kD
100kD
70kD
55kD
40kD
β-actin
180kD
130kD
100kD
70kD
55kD
40kD

Figure 10

Figure 10B

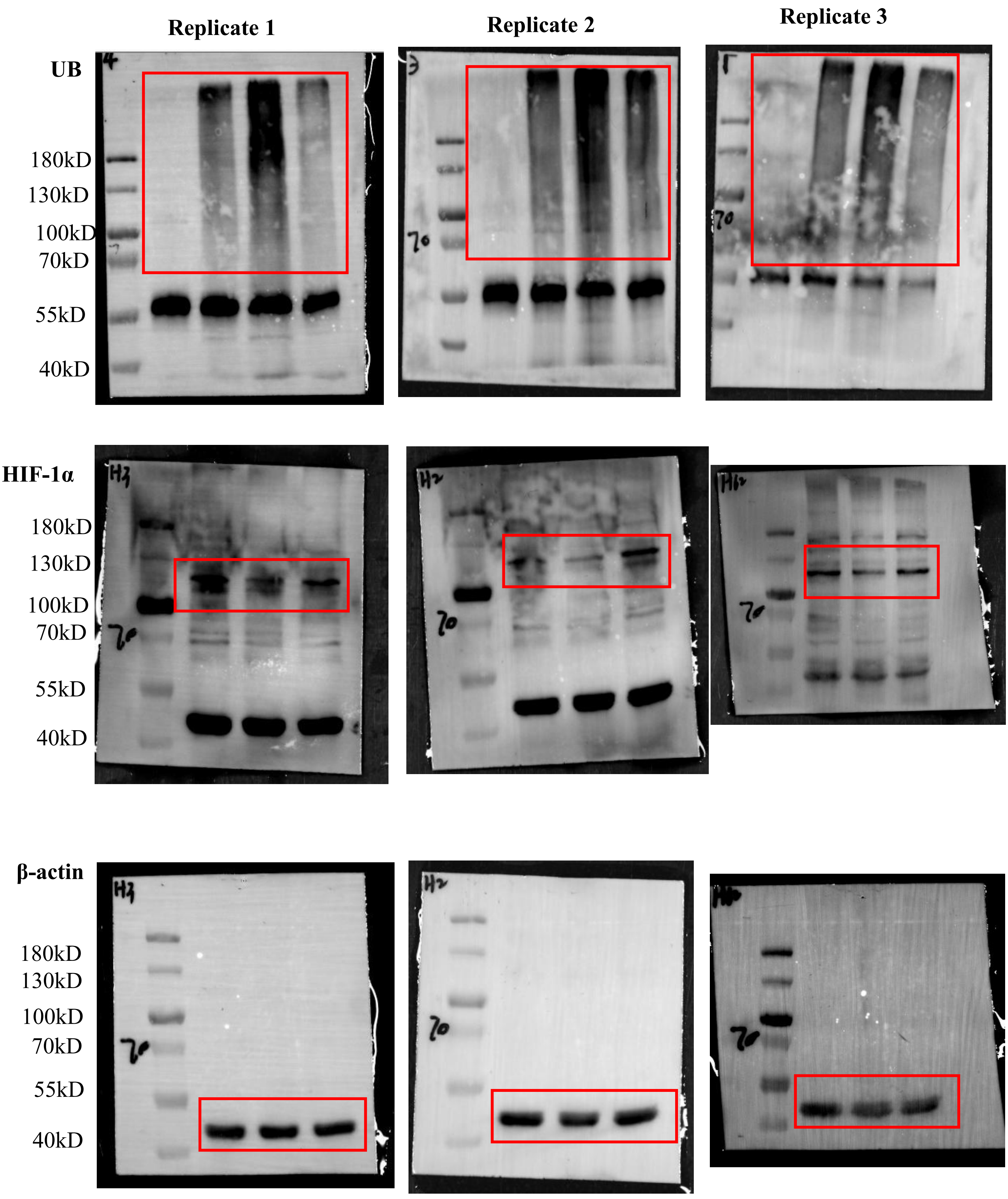

Figure 10C

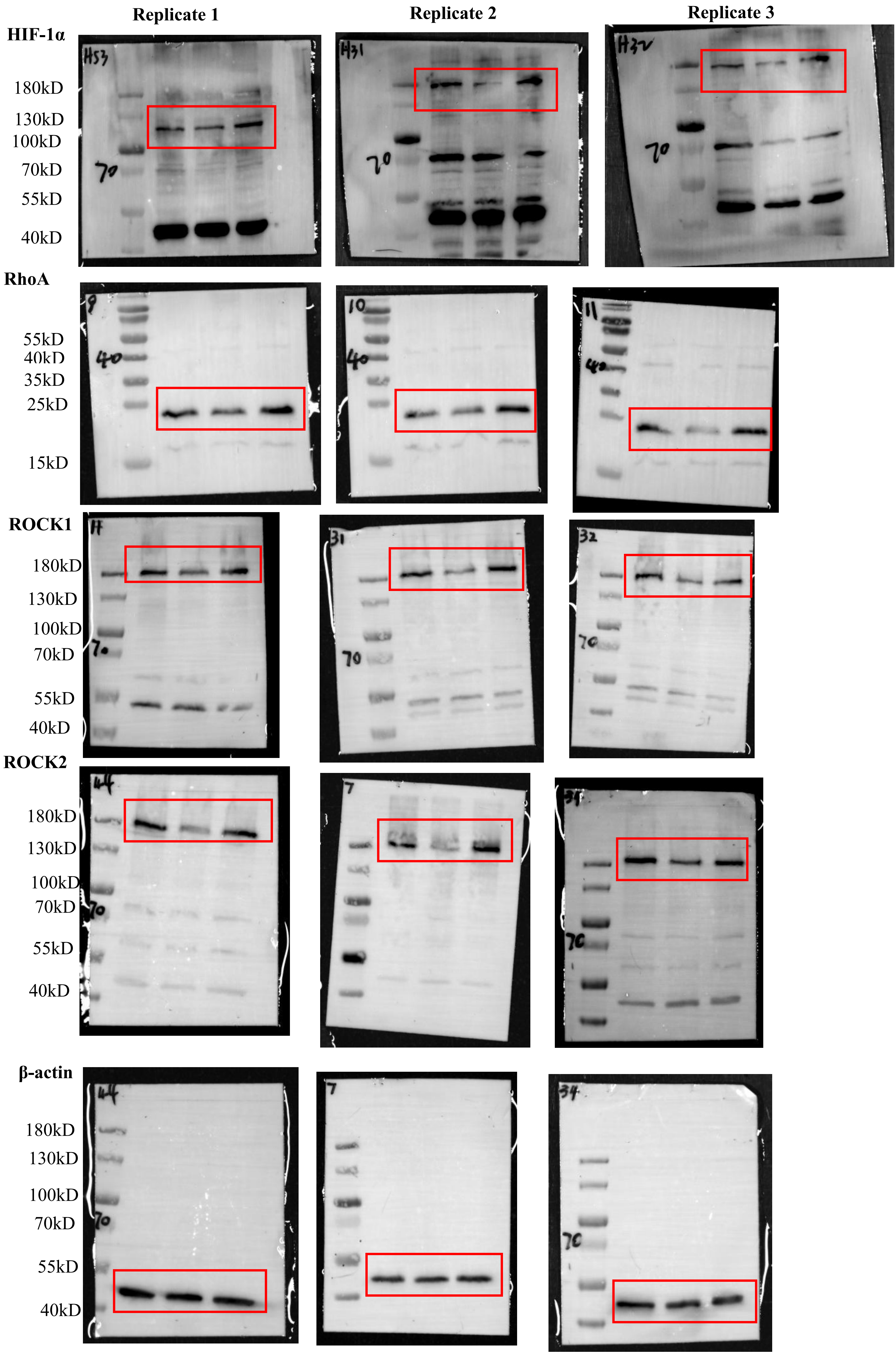

Replicate 1
Replicate 2
Replicate 3
HIF-1α
180kD
130kD
100kD
70kD
55kD
40kD
RhoA
55kD
40kD
35kD
25kD
15kD
ROCK1
180kD
130kD
100kD
70kD
55kD
40kD
ROCK2
180kD
130kD
100kD
70kD
55kD
40kD
β-actin
180kD
130kD
100kD
70kD
55kD
40kD

Figure 10E

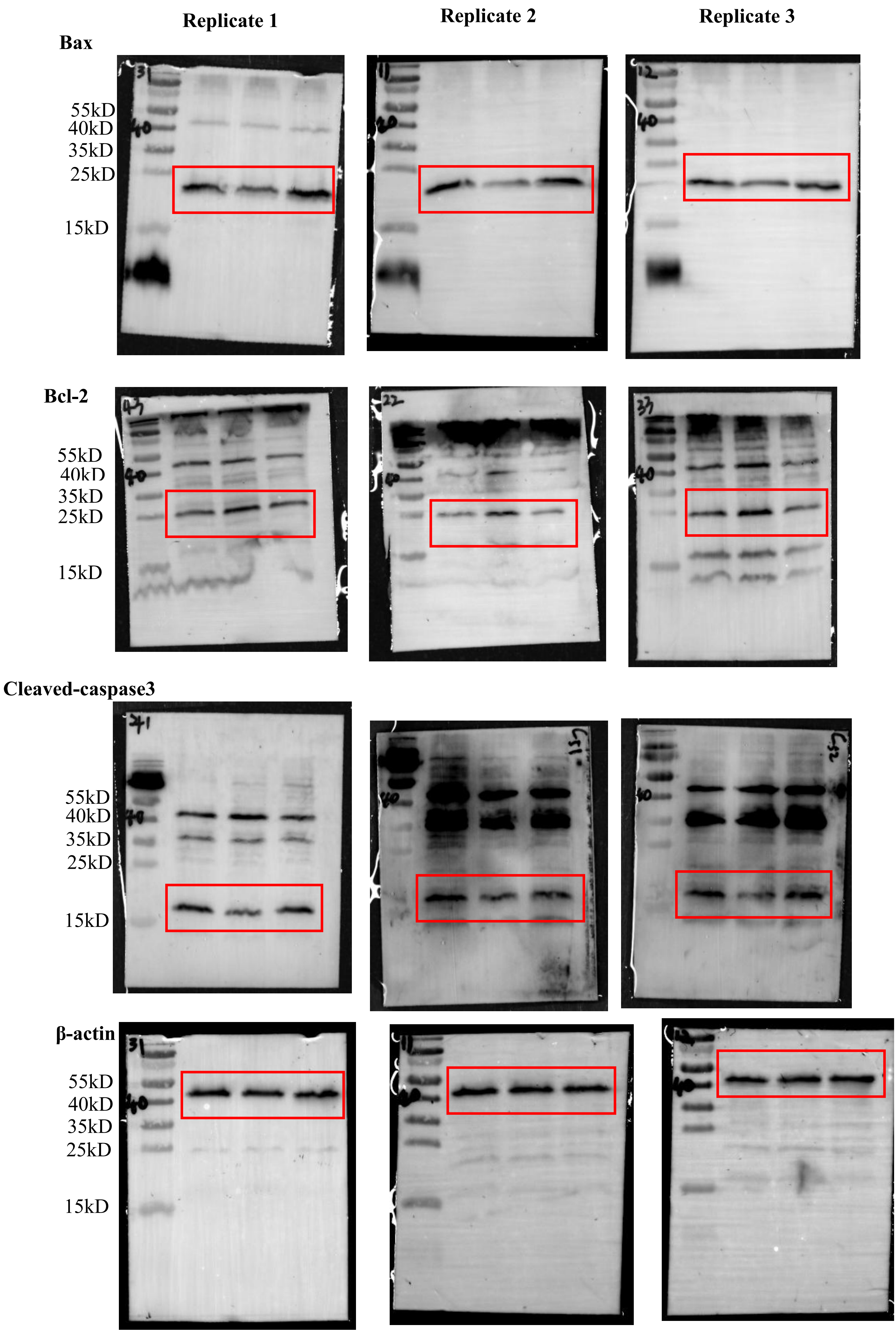

Replicate 1
Replicate 2
Replicate 3
Bax
55kD
40kD
35kD
25kD
15kD
Bcl-2
55kD
40kD
35kD
25kD
15kD
Cleaved-caspase3
55kD
40kD
35kD
25kD
15kD
β-actin
55kD
40kD
35kD
25kD
15kD

Figure 10G

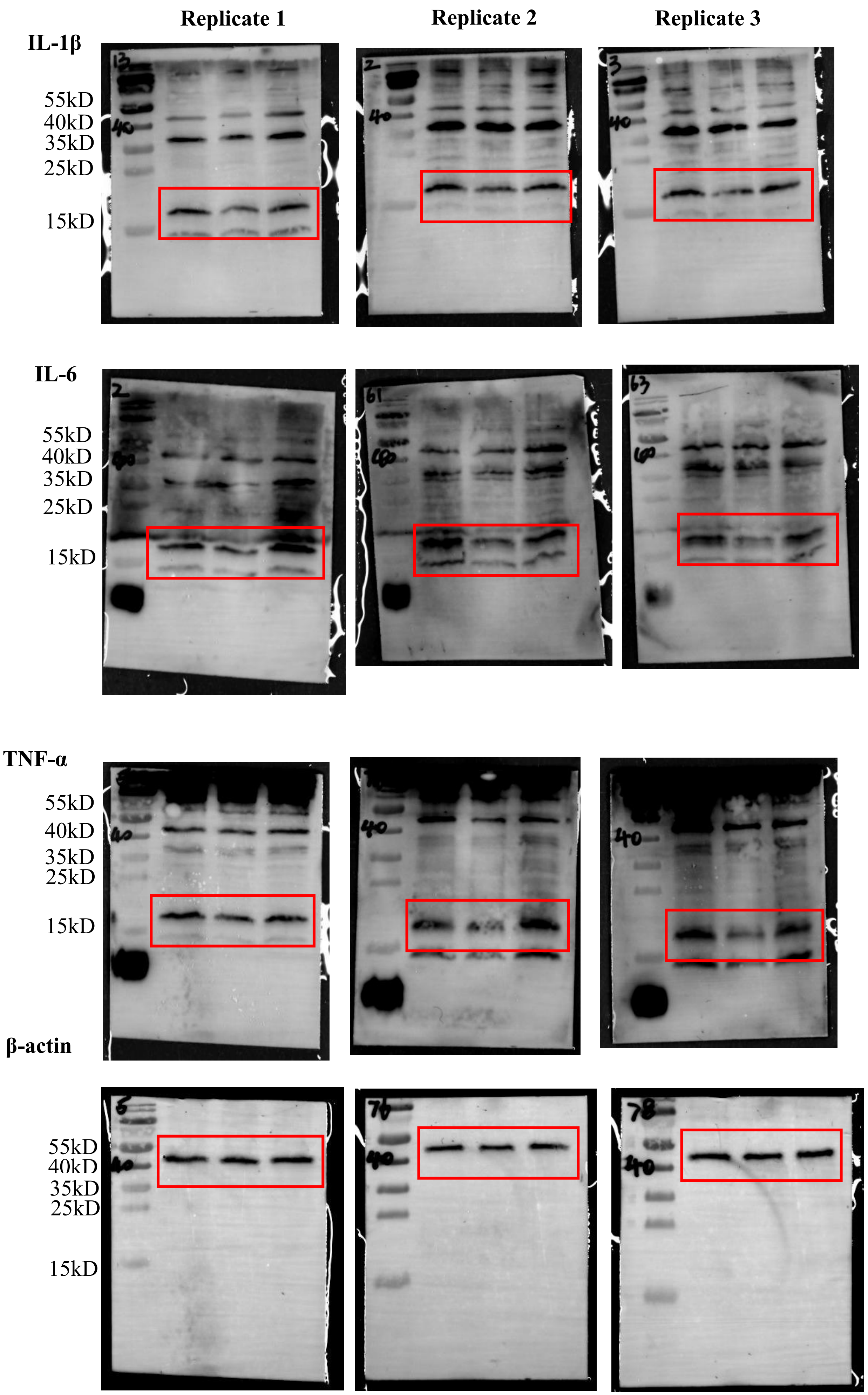

Replicate 1
Replicate 2
Replicate 3
IL-1β
55kD
40kD
35kD
25kD
15kD
IL-6
55kD
40kD
35kD
25kD
15kD
TNF-α
55kD
40kD
35kD
25kD
15kD
β-actin
55kD
40kD
35kD
25kD
15kD

Supplementary Figure 4

Supplementary Figure 4B

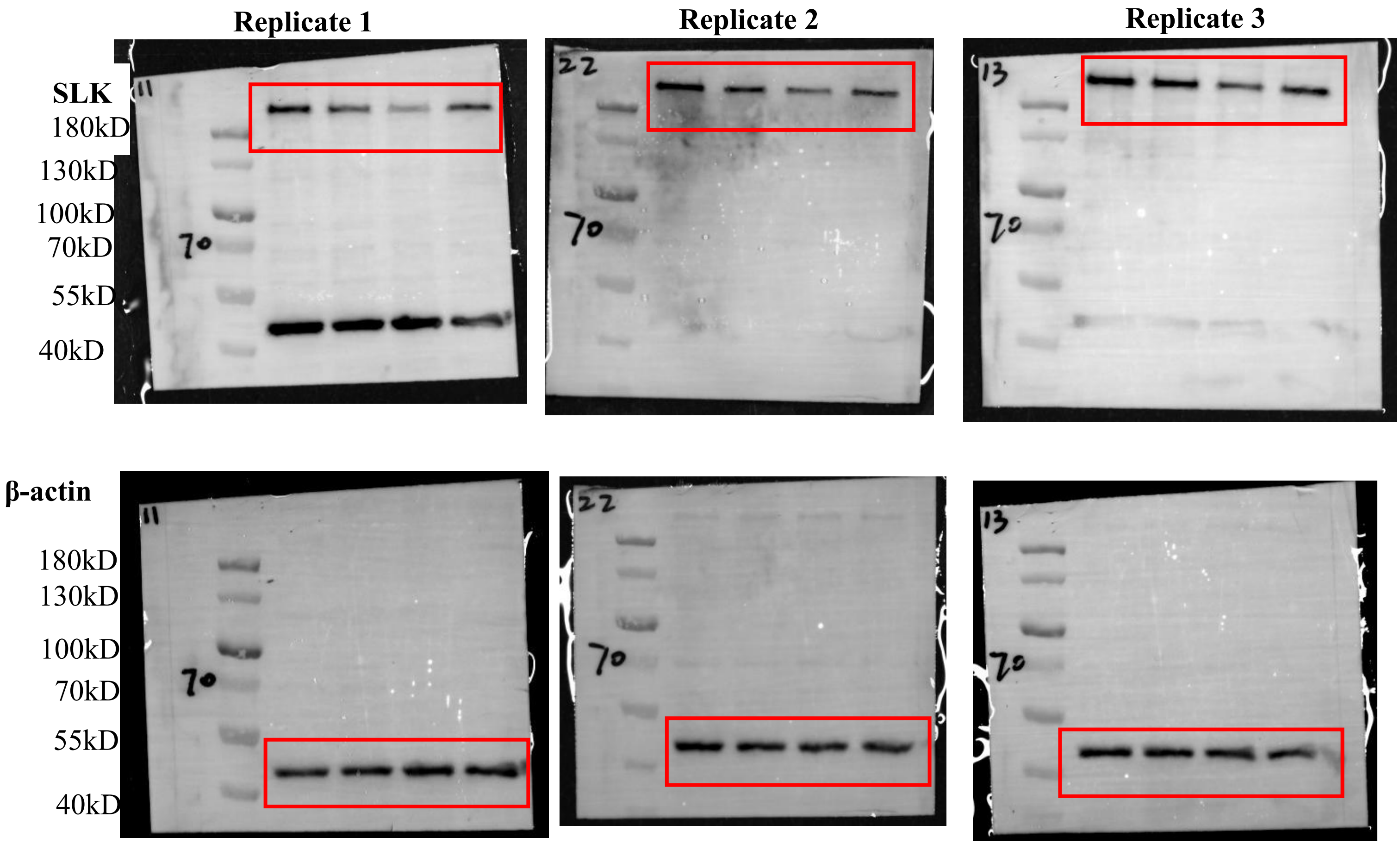

Supplementary Figure 4C

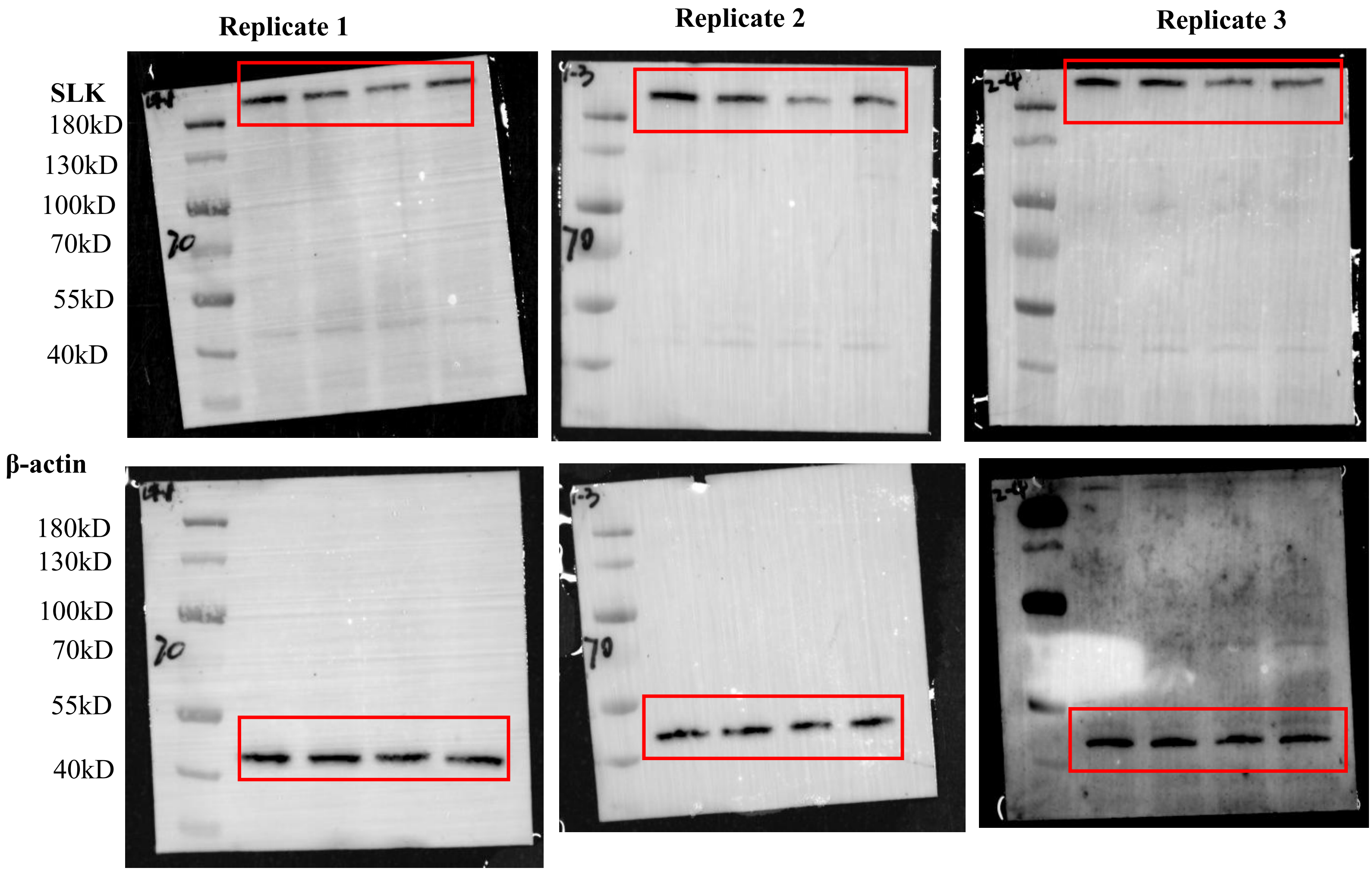

Supplementary Figure 5

left

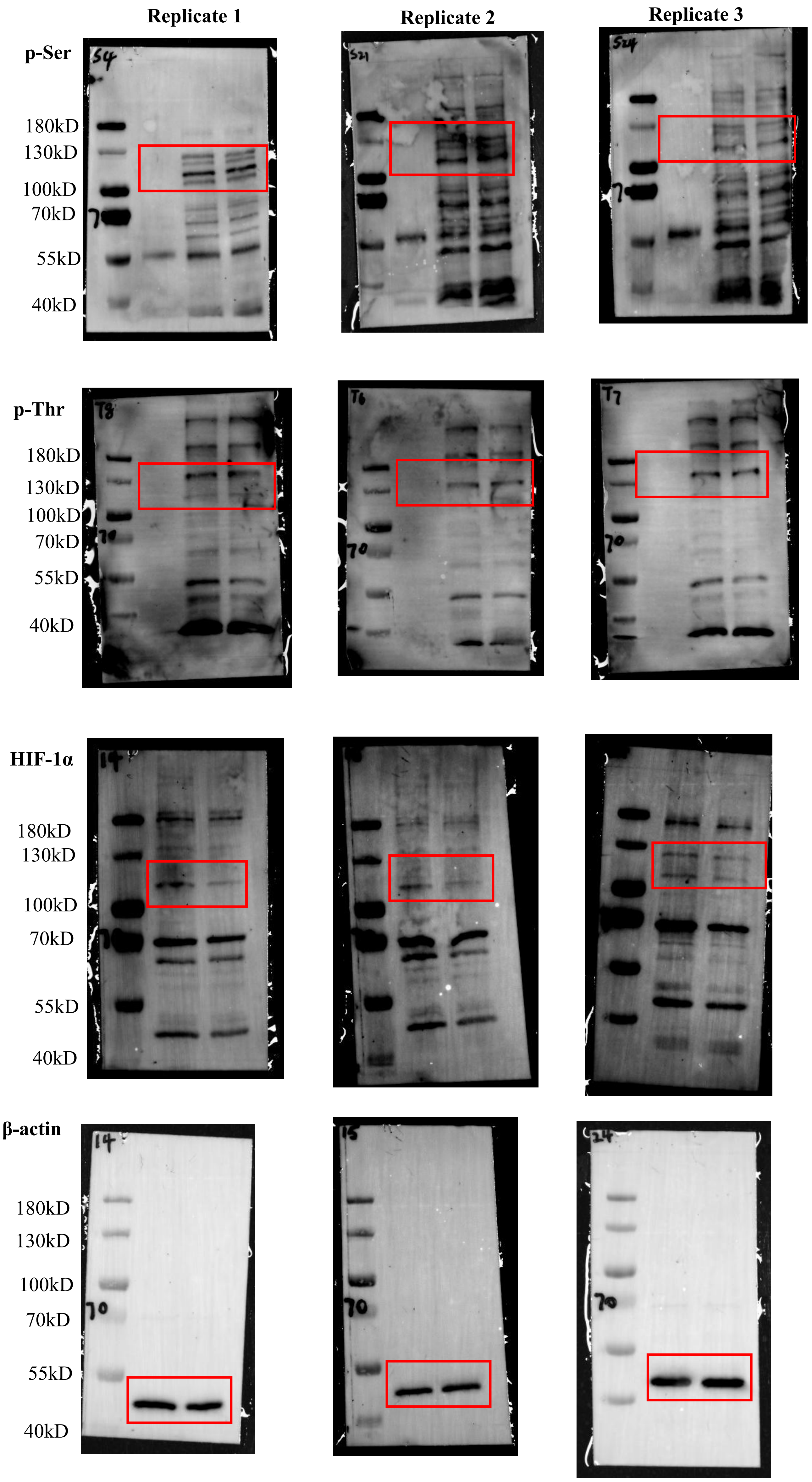

right

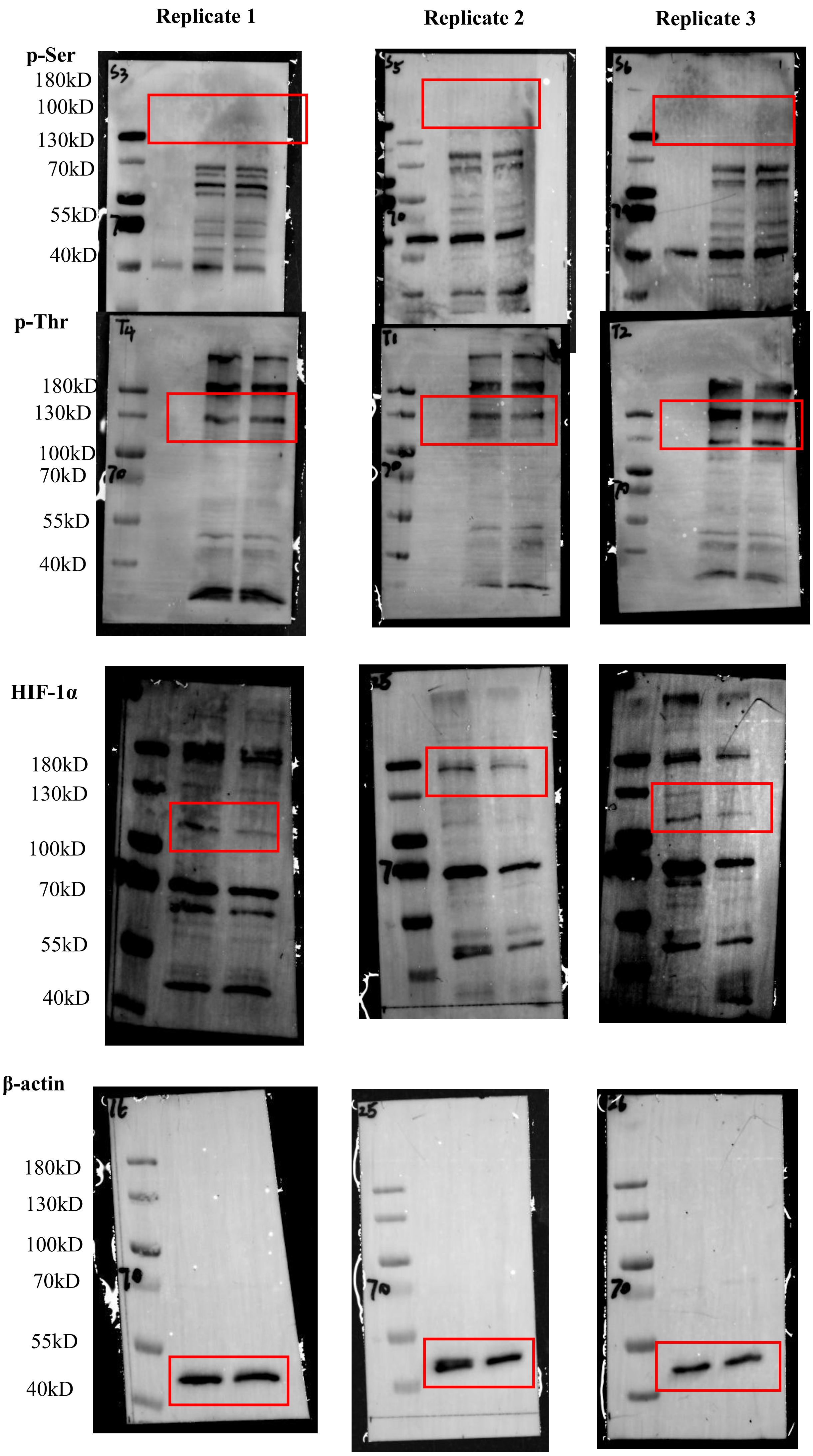
Replicate 1
Replicate 2
Replicate 3
p-Ser
180kD
100kD
130kD
70kD
55kD
40kD
p-Thr
180kD
130kD
100kD
70kD
55kD
40kD
HIF-1α
180kD
130kD
100kD
70kD
55kD
40kD
β-actin
180kD
130kD
100kD
70kD
55kD
40kD
